# Complexation and conformation of lead ion with poly-γ-glutamic acid in soluble state

**DOI:** 10.1371/journal.pone.0218742

**Published:** 2019-09-13

**Authors:** Lingling Wang, Yamin Liu, Xiulin Shu, Shunying Lu, Xiaobao Xie, Qingshan Shi

**Affiliations:** State Key Laboratory of Applied Microbiology Southern China, Guangdong Provincial Key Laboratory of Microbial Culture Collection and Application, Guangdong Open Laboratory of Applied Microbiology, Guangdong Institute of Microbiology, Guangzhou, China; University of Akron, UNITED STATES

## Abstract

Complexation of microbial polymer in soluble state could impact the solubility, mobility, and bioavailability of heavy metals in the environment. The complexation of a bacterial exopolymer, poly-γ-glutamic acid (γ-PGA), with Pb^2+^ was studied using the polarographic method and circular dichroism measurement in soluble state. The number of available binding sites was determined based on the Chau’s method and was found to be 0.04, 1.12, 3.56 and 4.51 mmol/(g dry weight of γ-PGA) at pH 3.4, 4.2, 5.0 and 6.2, respectively. Further, the number of binding sites was determined based on the Ruzic’s method and was found to be 3.60 and 4.41 mmol/(g dry weight of γ-PGA) for pH 5.0 and 6.2, respectively. The constant (expressed as log *K*) values were 5.8 and 6.0 at pH 5.0 and 6.2. Compared to biopolymers secreted by other microorganisms, such as extracellular polymeric substances extraction from activated sludge, γ-PGA was a more efficient Pb^2+^ carrier from pH 5.0 to 6.2. The secondary structure of γ-PGA varied significantly when Pb^2+^ added. Ca^2+^ or Mg^2+^ replace a portion of the adsorbed Pb^2+^. However, the portion of Pb^2+^ involved in changing the γ-PGA conformation was not easily replaced by Ca^2+^ and Mg^2+^.

## Introduction

Heavy metals are one of the most serious environmental problems in the world [1–3]. The negative effects of heavy metals on the ecosystem vary considerably and have economic and public health significance [4]. The primary sources of heavy metals are anthropogenic activities including agricultural, industrial, and sewage disposal processes [5–7]. Unlike organic contaminants, heavy metals do not undergo microbial or chemical degradation and persist for a long time to accumulate in the environment in substantial quantity. Heavy metals can be carried by stormwater run-off and transported to soil, rivers, lakes, seas, and oceans [6], causing a wide range of pollution.

Natural organic matter (NOM) dispersed in water systems and soils could bind heavy metals and transport them as a mobile "carrier" [8, 9]. The complexations of metals by NOM affects their speciation, mobility, and bioavailability. Among the NOM components, the humic substances have been studied intensively [10]. However, binding of metals by other components of NOM such as bacterial polymers is not well understood because they are secreted by various microorganisms and consist of complex organic substances [11, 12]. Nonetheless, bacterial polymers make up a significant fraction of NOM and are effective metal-binding agents for enhancing metal transport [11]. Since these carriers exist naturally in soil and water, knowledge of their effect on heavy metal mobility can greatly improve ecological risk assessment of heavy metals. In addition, the application of engineered biopolymers that can adsorb heavy metals could have potential use in the remediation of contaminated sites and wastewater treatment plants.

*Bacillus lichenformis*, which a common soil microbe, produces a poly-γ-glutamic acid (γ-PGA) exopolymer. This exopolymer is also produced by other *Bacillus* species, such as *B*. *subtilis*, *B*. *megaterium*, *B*. *pumilis*, *B*. *mojavensis*, *B*. *amyloliquefaciens*, and *B*. *anthracis* [13–15]. The molecular structure of γ-PGA contains amide linkages between the γ-amino and γ-carboxylic acid functional groups, leaving the γ-carboxylate group available for cation binding. The γ-PGA is of a particular research interest because of its potential implication in the removal of heavy metals in water treatment [16, 17]. The γ-PGA showed a great affinity to Cu(II), Al(III), Cr(III), Fe(III) and Pb(II), which induce flocculation [18–20]. While these studies have focused on flocculation ability of γ-PGA in relation to the wastewater treatments, there is no complexation of heavy metals with γ-PGA in diluted solutions, where the polymer-metal complex remains soluble and could enhance the mobility of heavy metals in the environment, has not been investigated in detail. Especially, the conformations and molecular interactions of complexation of heavy metals with *γ*-PGA in a soluble state may be different from that in a flocculent state [21].

The aim of this study was to determine the number of available binding sites, constants of complexation and related conformational changes of γ-PGA (isolated from *B*. *licheniformis*) with Pb^2+^ metal in a soluble state at different pH using the polarography method in the stripping mercury dropping electrode mode (SMDE) and circular dichroism (CD) measurement. Pb^2+^ was chosen for study as it is one of the most toxic heavy metals present in the environment and has previously been shown to have highly bound to γ-PGA [20]. In addition, the effects of divalent cations such as Ca^2+^ and Mg^2+^ on the binding amount and conformation of γ-PGA/Pb^2+^ complexation in a soluble state were also investigated in this study.

## Materials and methods

### γ-PGA purification

5% w/w γ-PGA solution produced from *B*. *licheniformis* ATCC 9945A was sourced from Guangdong Demay biotechnology Co., China. The γ-PGA solution was further concentrated and washed repeatedly with deionized water using a cross flow ultrafilter equipped with Millipore Pellicon 2 Cassette (10K Da molecular-weight cutoff polyethersulfone membrane). The resulting γ-PGA solution was then filtered through a 0.22 μm hydrophilic PTFE filter and lyophilized.

### γ-PGA characterization

The purity and glutamic acid content of γ-PGA were determined by amino acid analysis using thin layer chromatography (TLC) and colorimetric analysis after hydrolysis as described by Wang et al. [21]. Metal ions were analyzed using an inductively coupled plasma mass spectrometer (ICP-MS, 7700X, Agilent Inc., USA). The molecular weight was quantitatively determined using the gel-permeating chromatography (LC-20A, Shimadzu, Japan) equipped with a Waters Ultrahydrogel^TM^ Linear column and coupled with refractive index detectors. 0.01 M citric acid-sodium citrate buffer (pH 5.0) was used as an eluent at a flow rate of 0.5 mL/min. The column temperature was maintained at 35°C and injection volume was set at 10.0 μL, with a run time of 30 min. A set of monodisperse PEO standards (American Polymer Standards Co.) with peak molecular weights (*M*_p_) (ranging from 83,600 to 1,001,000) was used for the calibration.

### Circular dichroism (CD) measurements

CD measurements were performed based on our previously study [21] on a Chirascan spectrometer (Applied Photophysics Co., UK) using a quartz cell of 1 cm path length. CD spectra of 10 mg/L γ-PGA solutions were collected from 190 to 260 nm. Micro-quantities HCl and NaOH were used to adjust the pH. NaF was used to adjust the ionic strength, as NaCl would disrupt the signal of γ-PGA from 190 to 260 nm. CD spectra deconvolution software CDNN 2.1 (courtesy of Gerald Böhm, Martin-Luther-Universität Halle-Wittenberg, Germany) was used to analyze the secondary structure of γ-PGA.

### Polarographic measurement

The voltammetric methods are effective in determining the complexation of heavy metals with γ-PGA in a soluble state because of its intrinsic capability in distinguishing between the free and bound metal ions in solutions [22, 23]. Polarographic measurements were carried out with a 797 VA Computrace (Metrohm Co., Switzerland) fitted to a three-electrode arrangement consisting of a multi-mode electrode, an Ag/AgCl reference electrode, and a Pt auxiliary electrode. The instrumental parameters are listed in Table 1.

**Table 1:** Voltammetric parameters used for polarographic measurements.

Parameter	Setting
Mode	Differential pulse
Electrode	Stripping mercury dropping electrode
Scan rate	8 mV/s
Voltage step	4 mV
Voltage step time	0.5 s
Pulse amplitude	10 mV
Pulse time	40 ms
Potential range scanned	-0.6 to -0.2 V

The γ-PGA concentration was selected based on the total organic carbon (TOC) level of the natural water samples [24]. The solutions contained 10 mg/L γ-PGA and 10 mM NaNO_3_ as a supporting electrolyte. The initial volume of the solution in the analysis cell was 20 ml. The solution was agitated and adjusted to the desired pH by adding micro-quantities of NaOH or HNO_3_ solutions. The measurement was taken at 25°C and the responses of the system were recorded after the deaeration of the sample using 5 min of nitrogen bubbling. After the micro-additions (25 or 50 μl) of the metal ion stock solution, the solution was stirred to homogenize and readjust the pH of the sample. The response of the system was recorded after 5 min of nitrogen purge. The value of the γ-PGA blank was subtracted from the measured value of current *i*.

### Metal complexation

The theoretical study of the complexation equilibrium associated with the law of mass action can be described as shown in Eq 1, provided that only 1:1 complex can be formed:

M+L⇌ML (1)

where M is the free metal, L is the free ligand and ML is the ligand-metal complex.

The Chau’s method is widely used to estimate the number of available binding sites of the ligand [L]_0_ [25]. After each addition of the metal ion in the dilute solution of the studied polymer, the current intensity *i* corresponding to the free metal ion concentration, [M]_f_, was measured. Firstly, a part of the metal added to the solution was bound by γ-PGA. After the occupation of all the available binding sites of γ-PGA, the added metal remained in solution and the current intensity *i* showed a linear draw. The value of [L]_0_ was determined from the representation of *i* vs the metal ion total concentration introduced [M]_t_.

Another common method to determine the complexation was described by Ruzic [25]. In this approach, it is assumed that only 1:1 complex is formed once all the complexing sites of the ligand is saturated by the metal ions. The conditional stability constant (*K*) of the ML complex species formed can be expressed as follows:

$$K=\frac{\left[ML\right]}{\left[M]•[L\right]}=\frac{{\left[M\right]}_{t}‐{\left[M\right]}_{f}}{{\left[M\right]}_{f}•({\left[L\right]}_{0}‐({\left[M\right]}_{t}‐{\left[M\right]}_{f}))}$$ (2)

where [L]_0_ represents the number of available binding sites of the ligand. Further, Eq 2 can be redefined as:

$$\frac{{\left[M\right]}_{f}}{{\left[M\right]}_{t}‐{\left[M\right]}_{f}}=\frac{{\left[M\right]}_{f}}{{\left[L\right]}_{0}}+\frac{1}{K•{\left[L\right]}_{0}}$$ (3)

If the model is valid, the representation of [M]_f_ / ([M]_t_ -[M]_f_) vs [M]_f_ should be a straight line. The slope and intercept allow the calculation of [L]_0_ and *K*, respectively.

### Fourier transform infrared spectroscopy (FTIR) measurements

To obtain sufficient signal in infrared spectra, the γ-PGA/CaCl_2_ or γ-PGA/MgCl_2_ mixture solutions were conducted at 40 mM γ-PGA and 20 mM 40 mM, 80 mM CaCl_2_ or MgCl_2_. Solutions were adjusted to pH 6.5 using 0.1M NaOH and their infrared spectra were recorded on the Tensor II FTIR spectrometer equipped with RT-DLaTGS detector (Bruker Companies, Germany). A Horizontal Attenuated Total Reflectance (ATR) accessory (PIKE Technologies, Germany) was used to measure 1ml liquid sample. ATR-FTIR spectra were collected from 4000 to 400 cm^-1^ with 16 scans at a resolution of 4 cm^-1^. All final ATR-FTIR spectra were obtained by subtracting the reference spectrum of deionized water from the sample spectrum.

Precipitate was quickly appeared after the mixture of 1 mM γ-PGA and 1 mM PbCl_2_ at pH 5.0. The precipitate was filtered through 0.45 μm filter and washed with deionized water to remove residual ions. The prepared sample was then lyophilized and directly mixed into FTIR grade KBr powder for test. For comparison, 10 mM γ-PGA in deionized water was adjusted to pH 3.0, 5.0 and 7.0 using 0.1M HCl or NaOH, and lyophilized for the FTIR test. All the samples were recorded on a Tensor II FTIR instrument (Bruker Companies, Germany).

## Results and discussion

### γ-PGA characterization

The purity of γ-PGA was confirmed by the sole detection of glutamic acid in the TLC analysis. The glutamic acid content *C*_A_ was 7.00 ± 0.21 mmol/g. The metal ions in γ-PGA were Na, Mg, and K, the contents of which were 24.41 ± 0.27 mg/g, 0.23 ± 0.001 mg/g and 0.78 ± 0.02 mg/g, respectively. Other metals, including Pb, Cu, Ni, Cd, Cr, Al, Zn, and Ba, were not detected or negligible (< 0.02 mg/g) in the γ-PGA. Based on the glutamic acid and metal ion results, the γ-PGA was present primarily in the form of acids (γ-PGA-H; 5.94 mmol/g). A small proportion of the γ-PGA also existed in the form of sodium salt (γ-PGA-Na; 1.06 mmol/g). The purity of γ-PGA was determined as 93 wt % and the molecular weight (*M*_w_) was 2.59 × 10^5^ g/mol.

**Fig 1:**
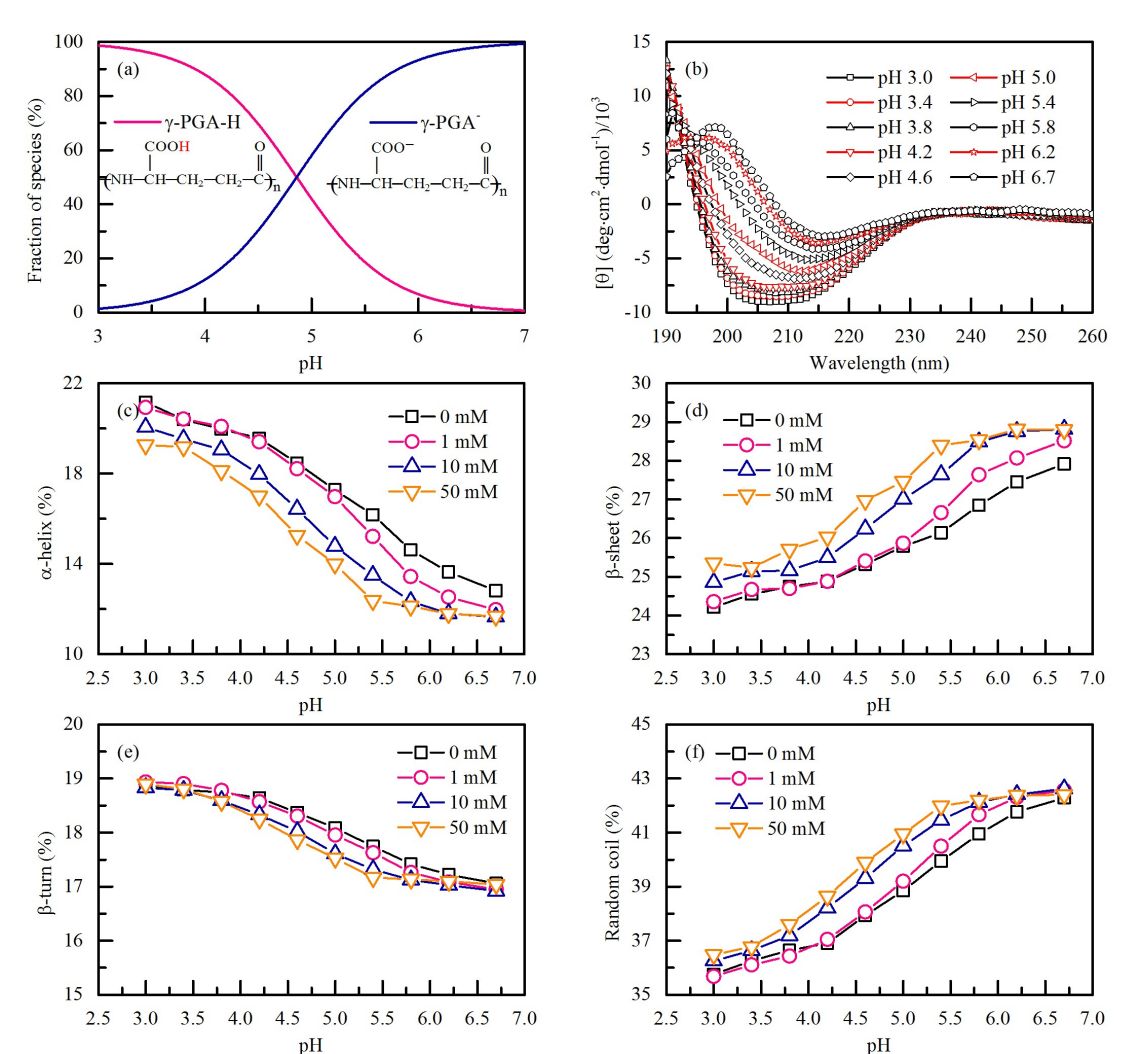
(a) Effect of pH on γ-PGA species. (b) Typical CD spectroscopy of 10 mg/L γ-PGA at different pH values (0 mM NaF). Effects of pH and ionic strength (0, 1, 10, 50 mM NaF) on the (c) α-helix, (d) β-sheet, (e) β-turn and (f) random coil structures of 10 mg/L γ-PGA.

The p*K*_a_ of γ-PGA was determined to be 4.86 [21] in our previous study and Fig 1(A) illustrates the effect of pH on γ-PGA species. The acid form γ-PGA-H rapidly ionized to form the mono-anionic γ-PGA^-^ specie as the pH increased from pH 3.0 to 7.0. Fig 1(B) shows the typical CD spectroscopy of **γ**-PGA at different pH values (0 mM NaF). The secondary structures of γ-PGA were determined using the CD spectra deconvolution software CDN 2.1 and shown in Fig 1(C)–1(F). The α-helix content decreased as pH increased from pH 3.0 to 6.7 and appeared to be highly related to the fraction of γ-PGA-H. The contents of β-sheet and random coil increased as pH increased from pH 3.0 to 6.7. The secondary structure of γ-PGA is governed by the intramolecular force [21]. In acidic condition, the γ-PGA structure is stabilized by intramolecular hydrogen bonds. There are two kinds of intramolecular hydrogen bonds, one is between the CO and the NH in the backbone, and another is between the side carboxylic oxygen and the backbone NH [26]. Theoretical models indicate that a left-handed helix with a 19-membered ring and hydrogen bonds set between the CO of the amide group *i* and the NH of amide group *i* + 3 is the most stable conformation. Weak intramolecular interactions between the side carboxylic oxygen and the NH of the backbone amide group are assumed to be responsible for the relatively high stability of the left-handed helix. The structure adopted by the γ-PGA molecule in higher pH may be attributed to two factors. Firstly, the deprotonated carboxylate groups destabilize and are therefore not available to maintain the hydrogen bonds between the side chains and the backbone. Secondly, increased negatively charged carboxylate group has a stronger electrostatic repulsion, which is resistant to the regular α-helix structure. Atomic Force Microscopy (AFM) images showed that rod-like γ-PGA acid form (γ-PGA-H) changed to sphere-like γ-PGA sodium form (γ-PGA-Na) because of the broken hydrogen bonds and increased electrostatic repulsion at higher pH [21]. Similar results were observed by others: Agresti et al. noted that the conformation of the poly(glutamic acid) changed from random coil to α-helix as the solution pH decreased from 7.2 to 3.0 [27], and γ-PGA of *B*. *licheniformis*is protonated and exhibits a helical conformation at a low pH, whereas a β-sheet-based conformation predominates at higher pH [28]. The effect of ionic strength on γγ-PGA conformation were determined at 0, 1, 10 and 50 mM NaF. Contrary to pH, ionic strength has little effect on γ-PGA conformation. The strong intramolecular hydrogen bonds seem to prevent the Na^+^ to replace the H^+^ in the COOH group.

### Determination of the number of available binding sites and the constants of γ-PGA/Pb^2+^ complexation

**Fig 2:**
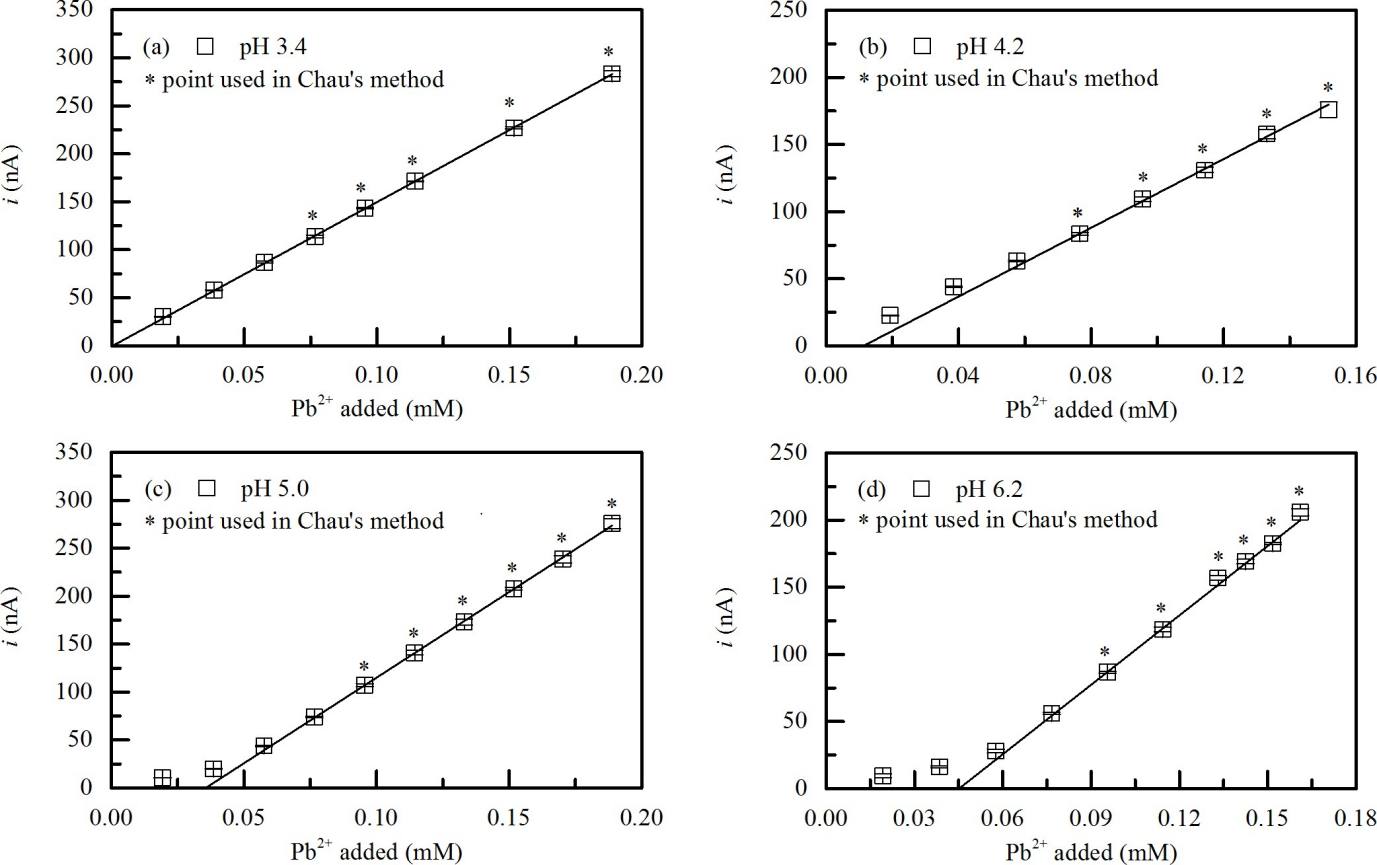
Plots of current *i* vs Pb^2+^ added for γ-PGA at (a) pH 3.4, (b) 4.2, (c) 5.0 and (d) 6.2 in 10 mM NaNO_3_.

In the γ-PGA/Pb^2+^ complexation test, ionic strength was selected at 10 mM because it had little effect on γ-PGA conformation and supporting electrolyte was necessary in the polarographic measurements. The pH values were chosen at 3.4, 4.2, 5.0 and 6.2 due to the γ-PGA conformations were distinct enough at these pH values as shown in Fig 1. In general, the curve of polarographic titration can be divided into two parts. In the first part, a portion of the metal ion added in solution is bound to the ligand, while, remaining metal ions were present in solution in the second part due to the saturation of all binding sites of ligand. Fig 2 shows the polarographic titration curves obtained for γ-PGA with Pb^2+^ at pH 3.4, 4.2, 5.0 and 6.2. At pH 3.4, the polarographic titration curve showed a linear variation for the entire concentration range because of the low adsorption of Pb^2+^ on γ-PGA. At pH 4.2, 5.0 and 6.2, the departure of plots in the first segment appeared, suggesting a complexation phenomenon before the saturation of the polymer is reached. After the saturation of the γ-PGA was achieved, the curve became linear. 5 to 6 plots in the second segment (* experimental point in Fig 2) were used for the modeling that was carried out according to Chau’s graphics method.

**Fig 3:**
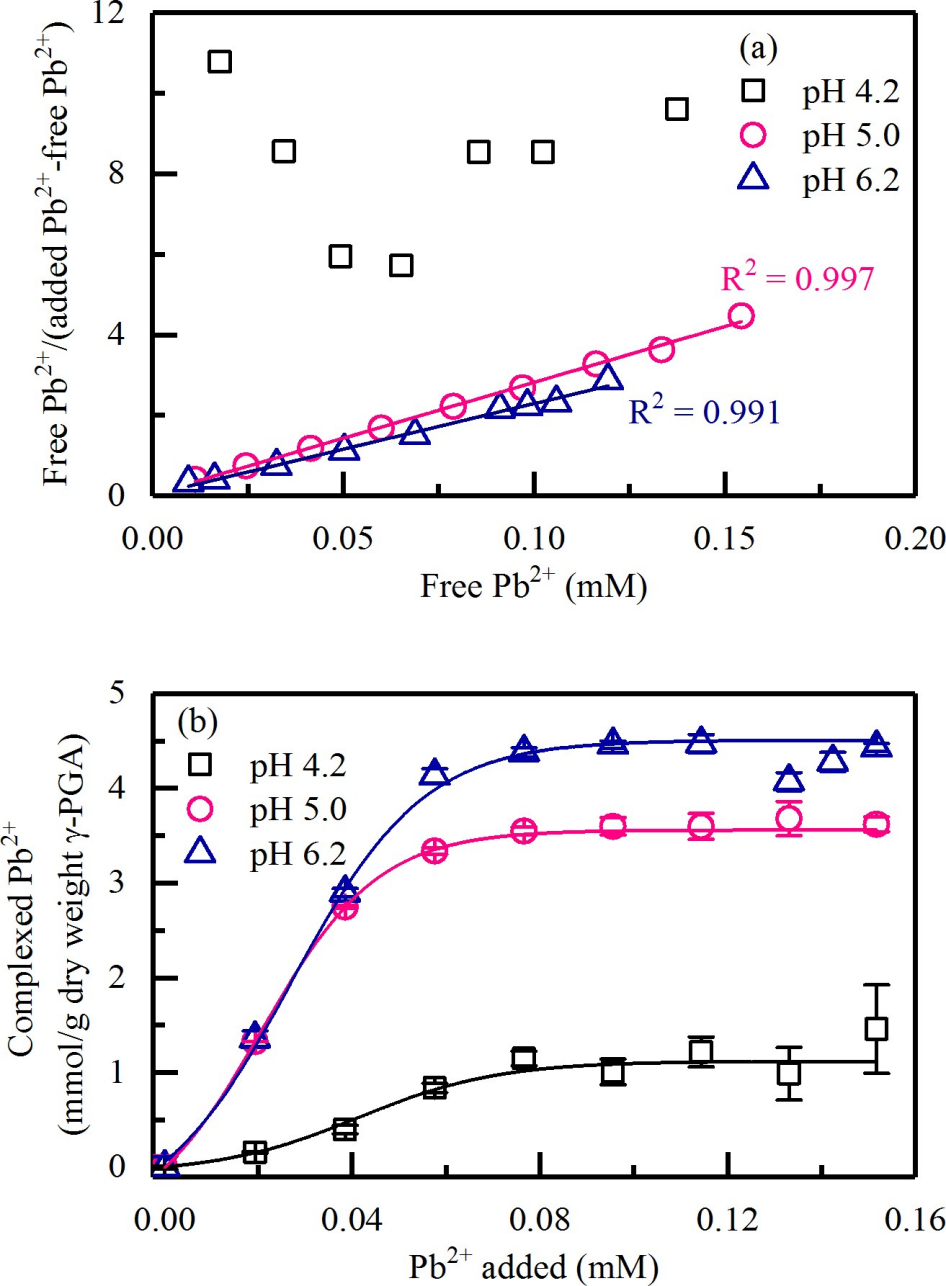
(a) Ruzic’s modeling carried on for γ-PGA/Pb^2+^ complexation at pH 4.2, 5.0 and 6.2. (b) Effects of pH on the amount of complexed Pb^2+^ with γ-PGA.

Fig 3(A) presents the Ruzic’s model for γ-PGA/Pb^2+^ complexation at pH 4.2, 5.0 and 6.2. Good determination coefficients (*>*0.99) were obtained at pH 5.0 and 6.2 from polarographic curves for the Ruzic’s method. The values of [L]_0_ and constant (expressed as log *K*) of γ-PGA/Pb^2+^ complexation were determined from the slope and intercept of the l line. The data points at pH 3.4 were not presented because the adsorbed metal ion ([M]_t_-[M]_f_) was near zero and the [M]_f_/([M]_t_-[M]_f_) value was very large and random (see S1 Table). The linearity of [M]_f_/([M]_t_-[M]_f_) vs [M]_f_ curve was poor at pH 4.2 for the initial several points. Similar problem was observed by Perret et al. in the polarographic study of polymer polyacrylic acid (PAA) complexed with lead ion [25]. The values calculated for [M]_f_/([M]_t_-[M]_f_) were questionable because of the low complexation of Pb^2+^ with γ-PGA, i.e., the very low value of adsorbed metal ion ([M]_t_-[M]_f_) for the initial several points. The amounts of Pb^2+^ complexed with γ-PGA at pH 4.2, 5.0 and 6.2 were present in Fig 3(B). They increased until the adsorption reached saturation due to the increase in the pH. For 10 mg/L γ-PGA, the Pb^2+^ adsorption was near saturated after the Pb^2+^ concentration reached 76 μM at pH 4.2, 5.0 and 6.2.

Table 2 shows the [L]_0_ values determined using the Chau’s and Ruzic’s methods and log *K* of γ-PGA/Pb^2+^ complexation determined using the Ruzic’s method. [L]_0_ increased with increased pH. The [L]_0_ values determined using the Chau’s method were 0.04, 1.12, 3.56 and 4.51 mmol/(g of dry weight of γ-PGA) for pH 3.4, 4.2, 5.0 and 6.2, respectively. The number of binding sites determined using the Ruzic’s method was 3.60 and 4.41 mmol/(g of dry weight of γ-PGA) for pH 5.0 and 6.2, respectively. [L]_0_ determined using the Chau’s method and the Ruzic’s method were consistent. The values of log *K* were 5.8 and 6.0 for pH 5.0 and 6.2, respectively.

**Table 2:** Determination of the number of available binding sites in mmol (g of dry weight of γ-PGA)^−1^ and the constant of γ-PGA/Pb^2+^ complexation.

pH	[L]_0_ (Chau’s method)	[L]_0_ (Ruzic’s method)	log*K* (Ruzic’s method)
3.4	0.04	-	-
4.2	1.12	-	-
5.0	3.56	3.60	5.8
6.2	4.51	4.41	6.0

The complexation of Pb^2+^ with γ-PGA was attributed to the negatively charged carboxylate groups in the polymer side chains. The acid form of γ-PGA was ionized to form the mono-anionic γ-PGA^-^ species as the pH increased, resulting in the releasing of more carboxylate groups to bind Pb^2+^. Consequently, [L]_0_ increased with the increase in pH. Carboxylate group content was equal to the glutamic acid content *C*_A_ (7.00 mmol/g). The occupied Pb^2+^ per carboxylate site were 0.16, 0.51 and 0.64 for pH 4.2, 5.0 and 6.2, respectively.

The values for [L]_0_ and log *K* of γ-PGA/Pb^2+^ complexation were compared with other polymer/Pb^2+^ systems obtained using the voltammetric methods presented in Table 3. A carboxylate group rich synthetic polymer PAA could fix up to 6.3 mmol Pb^2+^ (log *K* = 5.3) at pH 6.0 [25]. Occupied Pb^2+^ per carboxylate site for PAA was 0.45. Although the γ-PGA had a lower [L]_0_ than the PAA, occupied Pb^2+^ per carboxylate site for γ-PGA was greater than that for PAA, suggesting that γ-PGA was an efficient adsorbent for Pb^2+^. The values for [L]_0_ of extracellular polymeric substances (EPS) extracted from activated sludge or bacteria varied between 0.1 and 3.1 mmol/(g of dry weight EPS), while the values of log *K* varied between 3.2 and 5.7 [22, 29–31]. The values for [L]_0_ and log *K* of γ-PGA were greater than that of EPS found in the literature. This may due to carboxylate group has a high affinity to Pb^2+^ [25] and γ-PGA has a higher carboxylic site density (a carboxylate group per repeating unit of the polymer) compared to EPS. The composition of EPS are mainly polysaccharides and proteins, with along humic substances, nucleic acid, mineral compounds [32]. The presence of a substantial proportion of neutral residues in EPS reduce the site density of EPS. For example, the binding site densities of p*K*_a_ 2.44 (carboxylic acid), 6.36 (phosphoric groups), 7.57 (sulfinic acid, sulfonic acid or thiols), 10.16 (thiols, amino) were estimated to be 1.70, 0.14, 0.17, and 1.45 mmol g^-1^ dry weight EPS, respectively [33]. Accordingly, γ-PGA is a more efficient carrier for Pb^2+^ compared to biopolymers secreted by other microorganisms.

**Table 3:** Number of available binding sites for polymer/Pb^2+^ systems determined by polarographic method.

	pH	[L]_0_ mmol (g of dry weight) ^−1^	log*K*	Reference
PAA	6.0	6.3	5.3	[25]
EPS-S	7.0	2.1	4.9	[29]
EPS-B	7.0	0.4–1.8	3.2–5.0	[29]
Bound EPS	7.0	2.0–2.5	4.0–4.3	[30]
Soluble EPS	7.0	2.4–3.1	3.5–4.3	[30]
EPS-A	6.0	1.3–1.8	5.5–5.7	[31]
EPS-AF	5.0	0.1	4.9	[23]
γ-PGA	5.0	3.60	5.8	This study
γ-PGA	6.2	4.41	6.0	This study

PAA: polyacrylic acid, molecular weight, 3×10^6^ g/mol; EPS-S: extracellular polymeric substances (EPS) extraction from activated sludge; EPS-B: EPS extraction from 8 strains of bacteria; Bound EPS: bound EPS extraction from two types of activated sludges; Soluble EPS: soluble EPS extraction from two types of activated sludges; EPS-A: EPS extraction from two activated sludges obtained from two wastewater treatment plants; EPS-AF: EPS extraction from *Aspergillus fumigatus*.

### Effects of pH and Pb^2+^ concentrations on conformation of Pb^2+^ with γ-PGA

**Fig 4:**
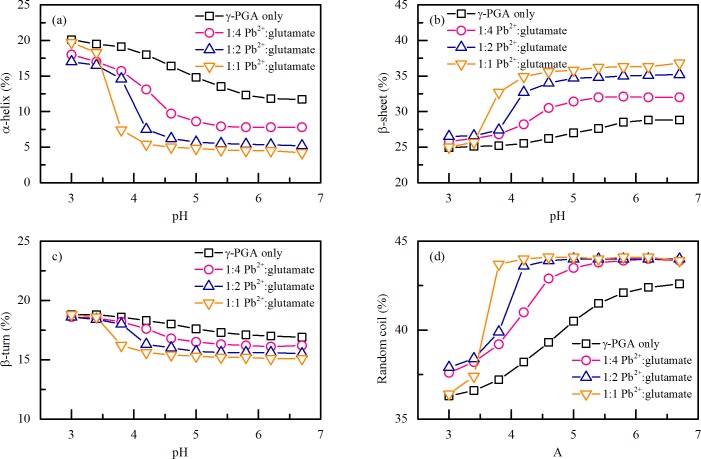
**Effects of pH and Pb**^**2+**^ **concentrations on the (a) α-helix, (b) β-sheet, (c) β-turn and (d) random coil structures of complexed Pb**^**2+**^ **with** γ**-PGA.** All experiments were conducted with 10 mg/L γ-PGA in 10 mM NaF. The concentrations of Pb^2+^ were 19, 38, 76 μM, respectively.

Fig 4 illustrate the effects of pH and Pb^2+^ concentrations on the secondary structure of Pb^2+^ complexed with γ-PGA. The ratio of Pb^2+^ to γ-PGA was normalized to moles of Pb^2+^ to moles of glutamate monomer and expressed as Pb^2+^:glutamate monomeric unit. The secondary structures of γ-PGA/Pb^2+^ complexation were significant different from that of γ-PGA. The secondary structure of γ-PGA at pH 3.0 consisted of 20.1% α-helix, 24.9% β-sheet, 18.8% β-turn, and 36.3% random coil. With the increase of pH, there was a decrease in the α-helix from 20.1% to 11.7% and an increase in the β-sheet and random coil from 24.9% to 28.8% and 36.3% to 42.6%, respectively. When Pb^2+^ were added, the content of α-helix and β-turn decreased, and the content of β-sheet and random coil increased. As discussed before, α-helix is stabilized by the intramolecular hydrogen bonds and γ-PGA is in acid form (γ-PGA-H) at pH 3.0. There was no COO^-^ for Pb^2+^ to complexed at pH 3.0. As the pH increased, the ionization of COOH groups made COO^-^ available for Pb^2+^ complexation. In addition, the structure evolution from rod-like to sphere-like at higher pH may reduce the steric force for Pb^2+^ to complex easily.

Complexation of Pb^2+^ with γ-PGA changed the secondary structure more significantly as pH increased from pH 3.0 to 6.7. As mentioned above, there are two kinds of intramolecular hydrogen bonds to stabilize the γ-PGA structure in acidic condition. The intramolecular hydrogen bond between the side carboxylic oxygen and the backbone NH destroyed due to the deprotonation of carboxylate groups as pH increased. However, the hydrogen bond between the CO and the NH in the backbone might be still stable. Therefore, the α-helix content of γ-PGA decreased from 20.1% to 11.7% as pH increased from 3.0 to 6.7. After 76 μM Pb^2+^ addition, the large hydrated Pb^2+^ complexed to carboxylate groups may disrupt the hydrogen bond between the CO and the NH in the backbone because of steric hindrance effect. The improved complexation of Pb^2+^ with γ-PGA at higher pH lead to decrease the α-helix content of γ-PGA rapidly from 19.7% to 5.4% as pH increased from 3.0 to 4.2.

It is worth noting that when the pH is higher than 5.0 (approximate to the p*K*_a_ value 4.86) and Pb^2+^: glutamate ratios were 1:1 and 2:1, the secondary structure of γ-PGA/Pb^2+^ complexation were similar and nearly independent of pH. For example, at pH 6.2, the contents of α-helix, β-sheet, β-turn and random coil were 5.3%, 35.1%, 15.6% and 44.0% at 1:1 Pb^2+^: glutamate ratio while these results were 4.5%, 36.3%, 15.1% and 44.1% at 2:1 Pb^2+^: glutamate ratio. As indicated in Fig 3(B), the Pb^2+^ adsorption was near saturated when the Pb^2+^ concentration reached 76 μM (2:1 Pb^2+^: glutamate ratio) at pH 6.2. The amounts of Pb^2+^ complexed with γ-PGA were 2.9 and 4.4 mmol/(g of dry weight of γ-PGA) (Fig 3(B)) at 1:1 and 2:1 Pb^2+^: glutamate ratios at pH 6.2. The similar conformations and significantly different amounts of Pb^2+^ complexed with γ-PGA at 1:1 and 2:1 Pb^2+^: glutamate ratios indicates that a part of the adsorbed Pb^2+^ involved in changing the γ-PGA configuration when the Pb^2+^ adsorption was saturated from pH 5.0 to 6.7.

### Effects of divalent cation on complexation and conformation of Pb^2+^ with γ-PGA

**Fig 5:**
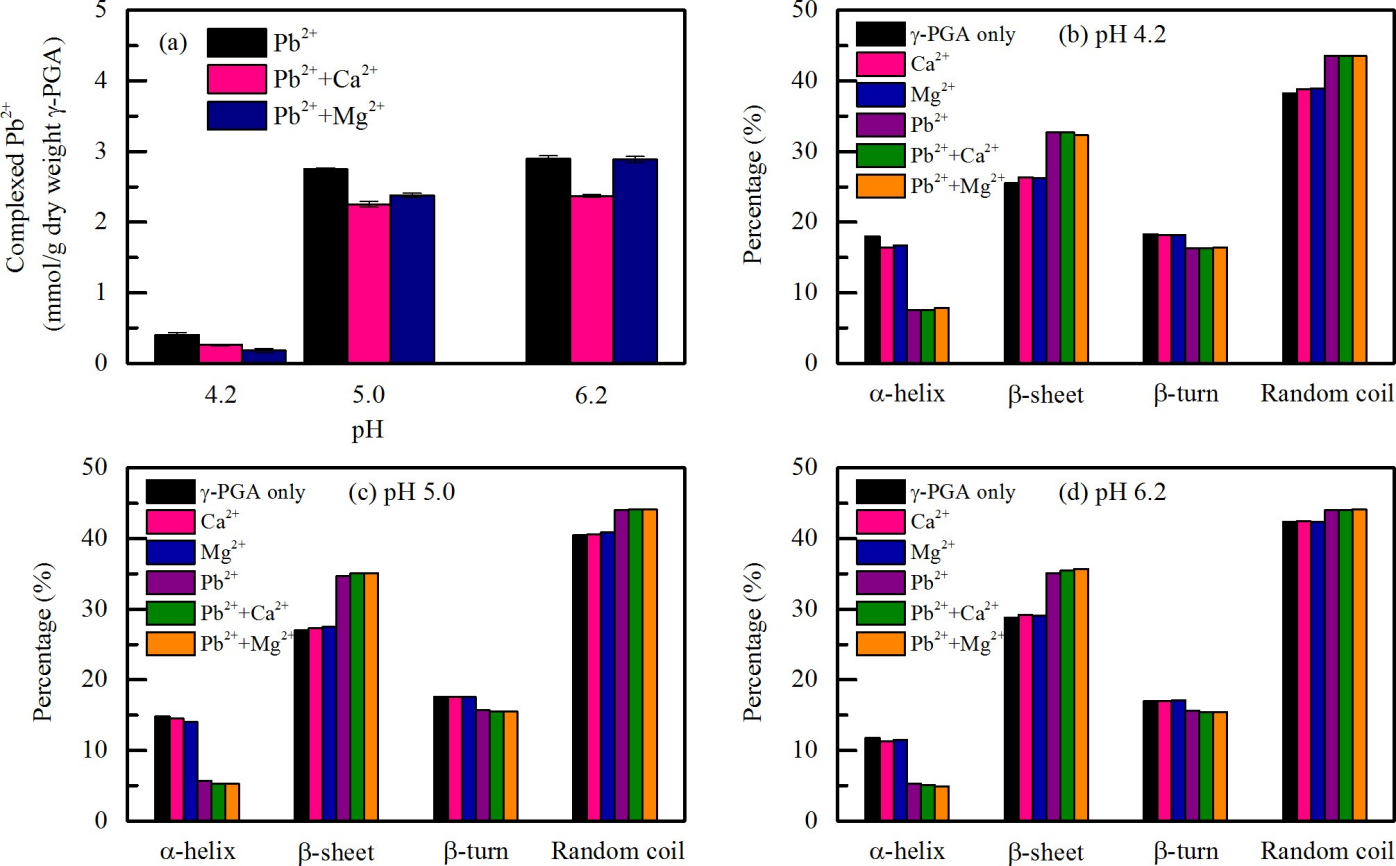
**(a) Effects of divalent cations (Ca**^**2+**^ **and Mg**^**2+**^**) on amount of complexed Pb**^**2+**^ **with γ-PGA at pH 4.2, 5.0 and 6.2; effects of divalent cations (Ca**^**2+**^ **and Mg**^**2+**^**) on α-helix, β-sheet, β-turn and random coil structures of γ-PGA and γ-PGA/Pb**^**2+**^ **complex at (b) pH 4.2; (c) pH 5.0 and (d) pH 6.2.** All experiments were conducted with 10 mg/L γ-PGA in 10 mM NaNO_3_ (polarographic measurement) or 10 mM NaF (CD measurement). The concentrations of Pb^2+^, Ca^2+^ and Mg^2+^ were 38 μM.

The effects of divalent cations such as Ca^2+^ and Mg^2+^ on complexation of Pb^2+^ with γ-PGA were determined at 38 μM Pb^2+^ under different pH (4.2, 5.0 and 6.2). The molar ratios of Pb^2+^ to Ca^2+^ and Pb^2+^ to Mg^2+^ were maintained at 1:1. As shown in Fig 5, the amounts of Pb^2+^ complexed with γ-PGA increased as the pH increased from 4.2 to 6.2 despite of Ca^2+^ or Mg^2+^ added to the solution. The amounts of Pb^2+^ complexed with γ-PGA were about 0.40, 2.75 and 2.90 mmol/(g of dry weight of γ-PGA) at pH 4.2, 5.0 and 6.2, respectively. The amounts of Pb^2+^ complexed with γ-PGA slightly decreased to 0.26, 2.25 and 2.37 mmol/(g of dry weight of γ-PGA) when Ca^2+^ was added. Similarly, the amounts of Pb^2+^ complexed with γ-PGA had a small decrease to 0.18, 2.39 and 2.89 mmol/(g of dry weight of γ-PGA) when Mg^2+^ was added.

As shown in Fig 5(B)–5(D), Ca^2+^ and Mg^2+^ have no significant effect on the conformations of the γ-PGA. Also, the secondary structure of γ-PGA/Pb^2+^ complex was not affected by Ca^2+^ or Mg^2+^. At pH 4.2, 5.0 and 6.2, the contents of α-helix, β-sheet, β-turn and random coil (Fig 5(B)–5(D)) did not change with the addition of Ca^2+^ or Mg^2+^. However, the amounts of Pb^2+^ complexed with γ-PGA slightly decreased when Ca^2+^ or Mg^2+^added (Fig 5(A)). This indicates that although Ca^2+^ and Mg^2+^ have an effect on the amount of Pb^2+^ complexed with γ-PGA, they have no effect on the conformation of γ-PGA/Pb^2+^ complex. The binding characteristics of γ-PGA with Ca^2+^and Mg^2+^ and the selectivity of lead by γ-PGA in the presence of Ca^2+^ and Mg^2+^ have been reported previously [20, 34]. The incorporation of Ca^2+^ or Mg^2+^ showed only a minor influence on lead binding, which was similar to our present study. The effect of cations on γ-PGA structure depend on the individual properties of ions, such as size, charge density, hydrated affinity and electronegativity [35]. Among the three ions, only Pb^2+^ has the ability to change the γ-PGA conformation. Such a difference might be associated with the following reasons: Firstly, Pb^2+^ has a higher electronegativity to attract the COO^-^ group [20]. Secondly, Pb^2+^ is found to be coordinated more H_2_O molecules [36]. Such a larger hydrated size attracted may disrupt the hydrogen bond between the CO and the NH in the backbone.

### FTIR spectra

**Fig 6:**
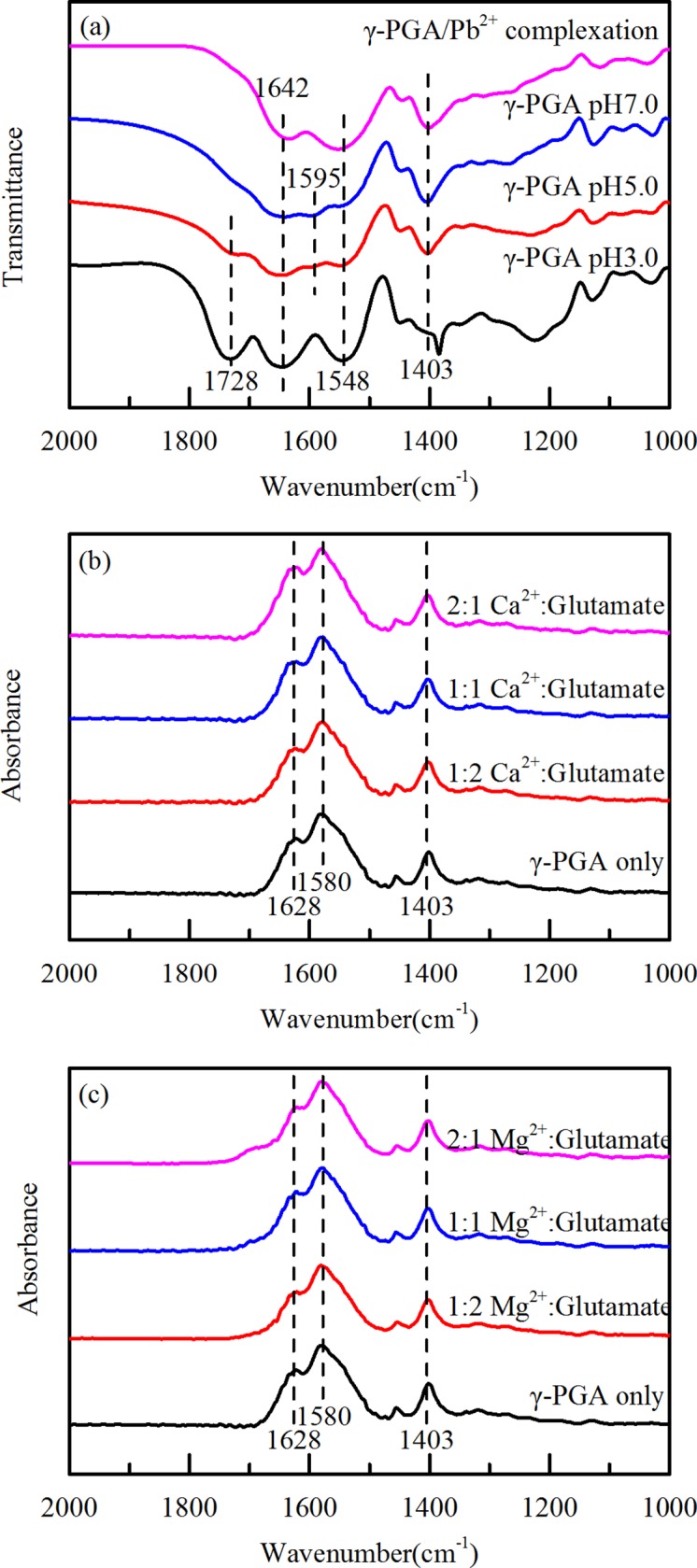
(a) FTIR spectra of γ-PGA/Pb^2+^ complexation, γ-PGA at pH 3.0, 5.0 and 7.0. (b) ATR-FTIR spectra of γ-PGA/Ca^2+^ mixture at varying Ca^2+^:glutamate ratios. (c) ATR-FTIR spectra of γ-PGA/Ca^2+^ mixture at varying Mg^2+^:glutamate ratios.

Metal ions complexed by the carboxylate of γ-PGA in a few different way, with the manner of complexation determining the symmetry, bond strength, and bond angle [28]. FTIR spectra were used to identify these frequency differences of the vibrational modes of the carboxylate group before and after the metal complexed to γ-PGA. As illustrated by Fig 6(A), the distinct peak around 1728 cm^-1^ in γ-PGA at pH 3.0, which was gradually disappeared at pH 5.0 and 7.0, was attributed to the C = O stretching in COOH. With the deprotonation of COOH, the C = O in the zwitterionic form COO^-^ shifted to about 1595 cm^-1^ (asymmetric stretching) and 1403 cm^-1^ (symmetric stretching) [37]. The intensity at 1595 cm^-1^ even overlapped the amide I (1643 cm^-1^) and amide II (1548 cm^-1^) bands at pH 5.0 and 7.0. After Pb^2+^ complexed, the COO^-^ asymmetric stretching shifted to about 1550 cm^-1^, overlapping the amide II band. This suggests that the Pb^2+^ strongly complexed with COO^-^ of γ-PGA and changed the vibrational modes of the carboxylate group.

Fig 6(B) and 6(C) depict the infrared spectra of Ca^2+^ and Mg^2+^ complexed with γ-PGA at pH 6.5 with different Ca^2+^:glutamate and Mg^2+^:glutamate ratios. The 1580 cm^-1^ and 1403 cm^-1^ bands correspond to the asymmetric stretching and symmetric stretching of COO^-^ in γ-PGA at pH 6.5. These two bands did not change with Ca^2+^:glutamate and Mg^2+^:glutamate range from 0.5 to 2. The unchanged ATR-FTIR bands are consistent with CD spectra and confirm that complex of Ca^2+^ or Mg^2+^ with γ-PGA have no effect on the γ-PGA conformation.

## Conclusions

The presence of microbial polymer has an impact on the solubility, mobility, and bioavailability of heavy metals in the environment. Polarographic study and CD measurement were conducted on the complexation of lead with a bacterial polymer (poly-γ-glutamic) acid at the TOC level of the natural water samples (10 mg/L). The models were developed using the Chau’s method and Ruzic’s method. The number of available binding sites [L]_0_ and log *K* of γ-PGA/Pb^2+^ complexation determined by the Chau’s method and Ruzic’s method were compared with other polymer/Pb^2+^ systems. The secondary structure of γ-PGA varied significantly when Pb^2+^ added. Divalent cations (Mg^2+^ and Ca^2+^) can replace a portion of the adsorbed Pb^2+^. However, the portion of Pb^2+^ involved in changing the γ-PGA conformation is not easily replaced by Ca^2+^ and Mg^2+^. γ-PGA has shown a high affinity to Pb^2+^ from pH 5.0 to 6.2, which should offer concern in carrying heavy metals in the water systems and in soils.

## Supporting information

S1 Table The raw data in Figs 1–6. (DOCX)
