# Supplementary material for: Complexation and conformation of lead ion with poly-γ-glutamic acid in soluble state
Source: PLoS One. 2019 Sep 13;14(9):e0218742. doi: 10.1371/journal.pone.0218742 (PMC6743753; doi:10.1371/journal.pone.0218742)
Supplement: S1 Table — (DOCX) [file pone.0218742.s001.docx]

1. Fig 1 datasets

(a) Fraction of γ-PGA species (%) datasets.

| pH | γ-PGA-H (%) | γ-PGA^-^ (%) |
| --- | --- | --- |
| 3 | 98.638 | 1.362 |
| 3.01 | 98.607 | 1.393 |
| 3.02 | 98.575 | 1.425 |
| 3.03 | 98.542 | 1.458 |
| 3.04 | 98.509 | 1.491 |
| 3.05 | 98.475 | 1.525 |
| 3.06 | 98.44 | 1.56 |
| 3.07 | 98.404 | 1.596 |
| 3.08 | 98.368 | 1.632 |
| 3.09 | 98.33 | 1.67 |
| 3.1 | 98.292 | 1.708 |
| 3.11 | 98.253 | 1.747 |
| 3.12 | 98.213 | 1.787 |
| 3.13 | 98.172 | 1.828 |
| 3.14 | 98.13 | 1.87 |
| 3.15 | 98.087 | 1.913 |
| 3.16 | 98.044 | 1.956 |
| 3.17 | 97.999 | 2.001 |
| 3.18 | 97.953 | 2.047 |
| 3.19 | 97.907 | 2.093 |
| 3.2 | 97.859 | 2.141 |
| 3.21 | 97.81 | 2.19 |
| 3.22 | 97.76 | 2.24 |
| 3.23 | 97.709 | 2.291 |
| 3.24 | 97.657 | 2.343 |
| 3.25 | 97.604 | 2.396 |
| 3.26 | 97.55 | 2.45 |
| 3.27 | 97.494 | 2.506 |
| 3.28 | 97.437 | 2.563 |
| 3.29 | 97.379 | 2.621 |
| 3.3 | 97.32 | 2.68 |
| 3.31 | 97.259 | 2.741 |
| 3.32 | 97.197 | 2.803 |
| 3.33 | 97.133 | 2.867 |
| 3.34 | 97.069 | 2.931 |
| 3.35 | 97.002 | 2.998 |
| 3.36 | 96.935 | 3.065 |
| 3.37 | 96.865 | 3.135 |
| 3.38 | 96.795 | 3.205 |
| 3.39 | 96.723 | 3.277 |
| 3.4 | 96.649 | 3.351 |
| 3.41 | 96.573 | 3.427 |
| 3.42 | 96.496 | 3.504 |
| 3.43 | 96.418 | 3.582 |
| 3.44 | 96.337 | 3.663 |
| 3.45 | 96.255 | 3.745 |
| 3.46 | 96.171 | 3.829 |
| 3.47 | 96.086 | 3.914 |
| 3.48 | 95.998 | 4.002 |
| 3.49 | 95.909 | 4.091 |
| 3.5 | 95.817 | 4.183 |
| 3.51 | 95.724 | 4.276 |
| 3.52 | 95.629 | 4.371 |
| 3.53 | 95.532 | 4.468 |
| 3.54 | 95.432 | 4.568 |
| 3.55 | 95.331 | 4.669 |
| 3.56 | 95.227 | 4.773 |
| 3.57 | 95.122 | 4.878 |
| 3.58 | 95.014 | 4.986 |
| 3.59 | 94.903 | 5.097 |
| 3.6 | 94.791 | 5.209 |
| 3.61 | 94.676 | 5.324 |
| 3.62 | 94.559 | 5.441 |
| 3.63 | 94.439 | 5.561 |
| 3.64 | 94.317 | 5.683 |
| 3.65 | 94.192 | 5.808 |
| 3.66 | 94.065 | 5.935 |
| 3.67 | 93.935 | 6.065 |
| 3.68 | 93.803 | 6.197 |
| 3.69 | 93.667 | 6.333 |
| 3.7 | 93.529 | 6.471 |
| 3.71 | 93.389 | 6.611 |
| 3.72 | 93.245 | 6.755 |
| 3.73 | 93.099 | 6.901 |
| 3.74 | 92.949 | 7.051 |
| 3.75 | 92.797 | 7.203 |
| 3.76 | 92.641 | 7.359 |
| 3.77 | 92.483 | 7.517 |
| 3.78 | 92.321 | 7.679 |
| 3.79 | 92.156 | 7.844 |
| 3.8 | 91.988 | 8.012 |
| 3.81 | 91.817 | 8.183 |
| 3.82 | 91.642 | 8.358 |
| 3.83 | 91.464 | 8.536 |
| 3.84 | 91.283 | 8.717 |
| 3.85 | 91.098 | 8.902 |
| 3.86 | 90.909 | 9.091 |
| 3.87 | 90.717 | 9.283 |
| 3.88 | 90.521 | 9.479 |
| 3.89 | 90.322 | 9.678 |
| 3.9 | 90.119 | 9.881 |
| 3.91 | 89.912 | 10.088 |
| 3.92 | 89.701 | 10.299 |
| 3.93 | 89.486 | 10.514 |
| 3.94 | 89.268 | 10.732 |
| 3.95 | 89.045 | 10.955 |
| 3.96 | 88.818 | 11.182 |
| 3.97 | 88.588 | 11.412 |
| 3.98 | 88.353 | 11.647 |
| 3.99 | 88.114 | 11.886 |
| 4 | 87.871 | 12.13 |
| 4.01 | 87.623 | 12.377 |
| 4.02 | 87.371 | 12.629 |
| 4.03 | 87.115 | 12.885 |
| 4.04 | 86.854 | 13.146 |
| 4.05 | 86.589 | 13.411 |
| 4.06 | 86.319 | 13.681 |
| 4.07 | 86.045 | 13.955 |
| 4.08 | 85.766 | 14.234 |
| 4.09 | 85.483 | 14.517 |
| 4.1 | 85.195 | 14.805 |
| 4.11 | 84.902 | 15.098 |
| 4.12 | 84.605 | 15.395 |
| 4.13 | 84.302 | 15.698 |
| 4.14 | 83.995 | 16.005 |
| 4.15 | 83.683 | 16.317 |
| 4.16 | 83.366 | 16.634 |
| 4.17 | 83.044 | 16.956 |
| 4.18 | 82.718 | 17.282 |
| 4.19 | 82.386 | 17.614 |
| 4.2 | 82.05 | 17.95 |
| 4.21 | 81.708 | 18.292 |
| 4.22 | 81.361 | 18.639 |
| 4.23 | 81.01 | 18.99 |
| 4.24 | 80.653 | 19.347 |
| 4.25 | 80.291 | 19.709 |
| 4.26 | 79.924 | 20.076 |
| 4.27 | 79.552 | 20.448 |
| 4.28 | 79.175 | 20.825 |
| 4.29 | 78.793 | 21.207 |
| 4.3 | 78.405 | 21.595 |
| 4.31 | 78.013 | 21.987 |
| 4.32 | 77.615 | 22.385 |
| 4.33 | 77.213 | 22.787 |
| 4.34 | 76.805 | 23.195 |
| 4.35 | 76.392 | 23.608 |
| 4.36 | 75.975 | 24.025 |
| 4.37 | 75.552 | 24.448 |
| 4.38 | 75.124 | 24.876 |
| 4.39 | 74.691 | 25.309 |
| 4.4 | 74.254 | 25.746 |
| 4.41 | 73.811 | 26.189 |
| 4.42 | 73.363 | 26.637 |
| 4.43 | 72.911 | 27.089 |
| 4.44 | 72.454 | 27.546 |
| 4.45 | 71.992 | 28.008 |
| 4.46 | 71.525 | 28.475 |
| 4.47 | 71.054 | 28.946 |
| 4.48 | 70.578 | 29.422 |
| 4.49 | 70.098 | 29.902 |
| 4.5 | 69.613 | 30.387 |
| 4.51 | 69.124 | 30.876 |
| 4.52 | 68.63 | 31.37 |
| 4.53 | 68.132 | 31.868 |
| 4.54 | 67.63 | 32.37 |
| 4.55 | 67.124 | 32.876 |
| 4.56 | 66.614 | 33.386 |
| 4.57 | 66.1 | 33.9 |
| 4.58 | 65.582 | 34.418 |
| 4.59 | 65.06 | 34.94 |
| 4.6 | 64.535 | 35.465 |
| 4.61 | 64.007 | 35.994 |
| 4.62 | 63.474 | 36.526 |
| 4.63 | 62.939 | 37.061 |
| 4.64 | 62.4 | 37.6 |
| 4.65 | 61.858 | 38.142 |
| 4.66 | 61.314 | 38.686 |
| 4.67 | 60.766 | 39.234 |
| 4.68 | 60.216 | 39.784 |
| 4.69 | 59.663 | 40.337 |
| 4.7 | 59.108 | 40.892 |
| 4.71 | 58.55 | 41.45 |
| 4.72 | 57.99 | 42.01 |
| 4.73 | 57.428 | 42.572 |
| 4.74 | 56.864 | 43.136 |
| 4.75 | 56.298 | 43.702 |
| 4.76 | 55.731 | 44.269 |
| 4.77 | 55.162 | 44.838 |
| 4.78 | 54.592 | 45.408 |
| 4.79 | 54.021 | 45.979 |
| 4.8 | 53.448 | 46.552 |
| 4.81 | 52.875 | 47.125 |
| 4.82 | 52.301 | 47.699 |
| 4.83 | 51.726 | 48.274 |
| 4.84 | 51.151 | 48.849 |
| 4.85 | 50.576 | 49.424 |
| 4.86 | 50 | 50 |
| 4.87 | 49.424 | 50.576 |
| 4.88 | 48.849 | 51.151 |
| 4.89 | 48.274 | 51.726 |
| 4.9 | 47.699 | 52.301 |
| 4.91 | 47.125 | 52.875 |
| 4.92 | 46.552 | 53.448 |
| 4.93 | 45.979 | 54.021 |
| 4.94 | 45.408 | 54.592 |
| 4.95 | 44.838 | 55.162 |
| 4.96 | 44.269 | 55.731 |
| 4.97 | 43.702 | 56.298 |
| 4.98 | 43.136 | 56.864 |
| 4.99 | 42.572 | 57.428 |
| 5 | 42.01 | 57.99 |
| 5.01 | 41.45 | 58.55 |
| 5.02 | 40.892 | 59.108 |
| 5.03 | 40.337 | 59.663 |
| 5.04 | 39.784 | 60.216 |
| 5.05 | 39.234 | 60.766 |
| 5.06 | 38.686 | 61.314 |
| 5.07 | 38.142 | 61.858 |
| 5.08 | 37.6 | 62.4 |
| 5.09 | 37.061 | 62.939 |
| 5.1 | 36.526 | 63.474 |
| 5.11 | 35.994 | 64.007 |
| 5.12 | 35.465 | 64.535 |
| 5.13 | 34.94 | 65.06 |
| 5.14 | 34.418 | 65.582 |
| 5.15 | 33.9 | 66.1 |
| 5.16 | 33.386 | 66.614 |
| 5.17 | 32.876 | 67.124 |
| 5.18 | 32.37 | 67.63 |
| 5.19 | 31.868 | 68.132 |
| 5.2 | 31.37 | 68.63 |
| 5.21 | 30.876 | 69.124 |
| 5.22 | 30.387 | 69.613 |
| 5.23 | 29.902 | 70.098 |
| 5.24 | 29.422 | 70.578 |
| 5.25 | 28.946 | 71.054 |
| 5.26 | 28.475 | 71.525 |
| 5.27 | 28.008 | 71.992 |
| 5.28 | 27.546 | 72.454 |
| 5.29 | 27.089 | 72.911 |
| 5.3 | 26.637 | 73.363 |
| 5.31 | 26.189 | 73.811 |
| 5.32 | 25.746 | 74.254 |
| 5.33 | 25.309 | 74.691 |
| 5.34 | 24.876 | 75.124 |
| 5.35 | 24.448 | 75.552 |
| 5.36 | 24.025 | 75.975 |
| 5.37 | 23.608 | 76.392 |
| 5.38 | 23.195 | 76.805 |
| 5.39 | 22.787 | 77.213 |
| 5.4 | 22.385 | 77.615 |
| 5.41 | 21.987 | 78.013 |
| 5.42 | 21.595 | 78.405 |
| 5.43 | 21.207 | 78.793 |
| 5.44 | 20.825 | 79.175 |
| 5.45 | 20.448 | 79.552 |
| 5.46 | 20.076 | 79.924 |
| 5.47 | 19.709 | 80.291 |
| 5.48 | 19.347 | 80.653 |
| 5.49 | 18.99 | 81.01 |
| 5.5 | 18.639 | 81.361 |
| 5.51 | 18.292 | 81.708 |
| 5.52 | 17.95 | 82.05 |
| 5.53 | 17.614 | 82.386 |
| 5.54 | 17.282 | 82.718 |
| 5.55 | 16.956 | 83.044 |
| 5.56 | 16.634 | 83.366 |
| 5.57 | 16.317 | 83.683 |
| 5.58 | 16.005 | 83.995 |
| 5.59 | 15.698 | 84.302 |
| 5.6 | 15.395 | 84.605 |
| 5.61 | 15.098 | 84.902 |
| 5.62 | 14.805 | 85.195 |
| 5.63 | 14.517 | 85.483 |
| 5.64 | 14.234 | 85.766 |
| 5.65 | 13.955 | 86.045 |
| 5.66 | 13.681 | 86.319 |
| 5.67 | 13.411 | 86.589 |
| 5.68 | 13.146 | 86.854 |
| 5.69 | 12.885 | 87.115 |
| 5.7 | 12.629 | 87.371 |
| 5.71 | 12.377 | 87.623 |
| 5.72 | 12.13 | 87.871 |
| 5.73 | 11.886 | 88.114 |
| 5.74 | 11.647 | 88.353 |
| 5.75 | 11.412 | 88.588 |
| 5.76 | 11.182 | 88.818 |
| 5.77 | 10.955 | 89.045 |
| 5.78 | 10.732 | 89.268 |
| 5.79 | 10.514 | 89.486 |
| 5.8 | 10.299 | 89.701 |
| 5.81 | 10.088 | 89.912 |
| 5.82 | 9.881 | 90.119 |
| 5.83 | 9.678 | 90.322 |
| 5.84 | 9.479 | 90.521 |
| 5.85 | 9.283 | 90.717 |
| 5.86 | 9.091 | 90.909 |
| 5.87 | 8.902 | 91.098 |
| 5.88 | 8.717 | 91.283 |
| 5.89 | 8.536 | 91.464 |
| 5.9 | 8.358 | 91.642 |
| 5.91 | 8.183 | 91.817 |
| 5.92 | 8.012 | 91.988 |
| 5.93 | 7.844 | 92.156 |
| 5.94 | 7.679 | 92.321 |
| 5.95 | 7.517 | 92.483 |
| 5.96 | 7.359 | 92.641 |
| 5.97 | 7.203 | 92.797 |
| 5.98 | 7.051 | 92.949 |
| 5.99 | 6.901 | 93.099 |
| 6 | 6.755 | 93.245 |
| 6.01 | 6.611 | 93.389 |
| 6.02 | 6.471 | 93.529 |
| 6.03 | 6.333 | 93.667 |
| 6.04 | 6.197 | 93.803 |
| 6.05 | 6.065 | 93.935 |
| 6.06 | 5.935 | 94.065 |
| 6.07 | 5.808 | 94.192 |
| 6.08 | 5.683 | 94.317 |
| 6.09 | 5.561 | 94.439 |
| 6.1 | 5.441 | 94.559 |
| 6.11 | 5.324 | 94.676 |
| 6.12 | 5.209 | 94.791 |
| 6.13 | 5.097 | 94.903 |
| 6.14 | 4.986 | 95.014 |
| 6.15 | 4.878 | 95.122 |
| 6.16 | 4.773 | 95.227 |
| 6.17 | 4.669 | 95.331 |
| 6.18 | 4.568 | 95.432 |
| 6.19 | 4.468 | 95.532 |
| 6.2 | 4.371 | 95.629 |
| 6.21 | 4.276 | 95.724 |
| 6.22 | 4.183 | 95.817 |
| 6.23 | 4.091 | 95.909 |
| 6.24 | 4.002 | 95.998 |
| 6.25 | 3.914 | 96.086 |
| 6.26 | 3.829 | 96.171 |
| 6.27 | 3.745 | 96.255 |
| 6.28 | 3.663 | 96.337 |
| 6.29 | 3.582 | 96.418 |
| 6.3 | 3.504 | 96.496 |
| 6.31 | 3.427 | 96.573 |
| 6.32 | 3.351 | 96.649 |
| 6.33 | 3.277 | 96.723 |
| 6.34 | 3.205 | 96.795 |
| 6.35 | 3.135 | 96.865 |
| 6.36 | 3.065 | 96.935 |
| 6.37 | 2.998 | 97.002 |
| 6.38 | 2.931 | 97.069 |
| 6.39 | 2.867 | 97.133 |
| 6.4 | 2.803 | 97.197 |
| 6.41 | 2.741 | 97.259 |
| 6.42 | 2.68 | 97.32 |
| 6.43 | 2.621 | 97.379 |
| 6.44 | 2.563 | 97.437 |
| 6.45 | 2.506 | 97.494 |
| 6.46 | 2.45 | 97.55 |
| 6.47 | 2.396 | 97.604 |
| 6.48 | 2.343 | 97.657 |
| 6.49 | 2.291 | 97.709 |
| 6.5 | 2.24 | 97.76 |
| 6.51 | 2.19 | 97.81 |
| 6.52 | 2.141 | 97.859 |
| 6.53 | 2.093 | 97.907 |
| 6.54 | 2.047 | 97.953 |
| 6.55 | 2.001 | 97.999 |
| 6.56 | 1.956 | 98.044 |
| 6.57 | 1.913 | 98.087 |
| 6.58 | 1.87 | 98.13 |
| 6.59 | 1.828 | 98.172 |
| 6.6 | 1.787 | 98.213 |
| 6.61 | 1.747 | 98.253 |
| 6.62 | 1.708 | 98.292 |
| 6.63 | 1.67 | 98.33 |
| 6.64 | 1.632 | 98.368 |
| 6.65 | 1.596 | 98.404 |
| 6.66 | 1.56 | 98.44 |
| 6.67 | 1.525 | 98.475 |
| 6.68 | 1.491 | 98.509 |
| 6.69 | 1.458 | 98.542 |
| 6.7 | 1.425 | 98.575 |
| 6.71 | 1.393 | 98.607 |
| 6.72 | 1.362 | 98.638 |
| 6.73 | 1.331 | 98.669 |
| 6.74 | 1.301 | 98.699 |
| 6.75 | 1.272 | 98.728 |
| 6.76 | 1.243 | 98.757 |
| 6.77 | 1.215 | 98.785 |
| 6.78 | 1.188 | 98.812 |
| 6.79 | 1.161 | 98.839 |
| 6.8 | 1.135 | 98.865 |
| 6.81 | 1.11 | 98.89 |
| 6.82 | 1.085 | 98.915 |
| 6.83 | 1.06 | 98.94 |
| 6.84 | 1.036 | 98.964 |
| 6.85 | 1.013 | 98.987 |
| 6.86 | 0.99 | 99.01 |
| 6.87 | 0.968 | 99.032 |
| 6.88 | 0.946 | 99.054 |
| 6.89 | 0.925 | 99.075 |
| 6.9 | 0.904 | 99.096 |
| 6.91 | 0.883 | 99.117 |
| 6.92 | 0.863 | 99.137 |
| 6.93 | 0.844 | 99.156 |
| 6.94 | 0.825 | 99.175 |
| 6.95 | 0.806 | 99.194 |
| 6.96 | 0.788 | 99.212 |
| 6.97 | 0.77 | 99.23 |
| 6.98 | 0.753 | 99.247 |
| 6.99 | 0.736 | 99.264 |
| 7 | 0.719 | 99.281 |

(b) CD spectroscopy of 10 mg/L γ-PGA at different pH values (0 mM NaF) datasets.

| Wavelength (nm) | pH 3.0 | pH 3.4 | pH 3.8 | pH 4.2 | pH 4.6 | pH 5.0 | pH 5.4 | pH 5.8 | pH 6.2 | pH 6.7 |
| --- | --- | --- | --- | --- | --- | --- | --- | --- | --- | --- |
| 190 | 11.224 | 12.942 | 13.294 | 12.568 | 12.058 | 10.895 | 8.32 | 6.013 | 4.913 | 2.544 |
| 191 | 10.092 | 9.847 | 10.753 | 10.932 | 10.199 | 9.917 | 9.248 | 8.429 | 5.596 | 3.801 |
| 192 | 7.033 | 7.319 | 8.07 | 8.701 | 8.364 | 8.887 | 7.996 | 7.088 | 5.736 | 4.18 |
| 193 | 4.324 | 4.945 | 5.662 | 6.532 | 6.81 | 7.519 | 7.235 | 6.733 | 6.113 | 4.867 |
| 194 | 1.934 | 2.595 | 3.393 | 4.423 | 5.116 | 6.111 | 6.466 | 6.4 | 6.161 | 5.66 |
| 195 | -0.251 | 0.444 | 1.188 | 2.372 | 3.444 | 4.628 | 5.55 | 5.781 | 6.123 | 6.129 |
| 196 | -2.141 | -1.522 | -0.752 | 0.492 | 1.918 | 3.153 | 4.965 | 5.673 | 6.178 | 6.636 |
| 197 | -3.763 | -3.3 | -2.585 | -1.249 | 0.49 | 1.986 | 4.166 | 5.317 | 6.118 | 7.069 |
| 198 | -5.312 | -4.746 | -4.158 | -2.834 | -0.733 | 0.61 | 3.458 | 4.696 | 5.898 | 7.109 |
| 199 | -6.462 | -5.906 | -5.322 | -4.107 | -1.834 | -0.429 | 2.605 | 4.065 | 5.519 | 7.029 |
| 200 | -7.327 | -6.838 | -6.177 | -5.199 | -2.785 | -1.43 | 1.741 | 3.45 | 5.197 | 6.458 |
| 201 | -8.007 | -7.476 | -6.711 | -6.011 | -3.63 | -2.172 | 0.833 | 2.661 | 4.53 | 5.683 |
| 202 | -8.463 | -7.946 | -7.075 | -6.598 | -4.245 | -2.796 | 0.007 | 1.847 | 3.707 | 4.849 |
| 203 | -8.759 | -8.224 | -7.412 | -6.965 | -4.948 | -3.308 | -0.795 | 1.055 | 2.83 | 3.851 |
| 204 | -8.914 | -8.341 | -7.58 | -7.272 | -5.434 | -3.743 | -1.572 | 0.365 | 1.987 | 3.121 |
| 205 | -8.98 | -8.428 | -7.825 | -7.559 | -5.755 | -4.069 | -2.262 | -0.397 | 1.172 | 2.33 |
| 206 | -8.983 | -8.437 | -8.001 | -7.664 | -5.986 | -4.581 | -2.852 | -1.146 | 0.254 | 1.537 |
| 207 | -9.03 | -8.482 | -8.135 | -7.667 | -6.333 | -4.93 | -3.428 | -1.837 | -0.497 | 0.742 |
| 208 | -8.97 | -8.519 | -8.206 | -7.644 | -6.512 | -5.341 | -3.876 | -2.34 | -1.117 | -0.132 |
| 209 | -8.972 | -8.532 | -8.132 | -7.606 | -6.681 | -5.664 | -4.252 | -2.874 | -1.721 | -0.918 |
| 210 | -8.91 | -8.467 | -8.141 | -7.663 | -6.738 | -5.92 | -4.554 | -3.221 | -2.345 | -1.556 |
| 211 | -8.864 | -8.341 | -8.109 | -7.578 | -6.865 | -6.073 | -4.756 | -3.492 | -2.751 | -1.983 |
| 212 | -8.794 | -8.219 | -7.951 | -7.533 | -6.878 | -6.194 | -5.005 | -3.688 | -3.109 | -2.247 |
| 213 | -8.646 | -8.081 | -7.812 | -7.573 | -6.793 | -6.131 | -5.114 | -3.939 | -3.358 | -2.638 |
| 214 | -8.402 | -7.972 | -7.643 | -7.427 | -6.705 | -6.039 | -5.102 | -4.124 | -3.574 | -2.8 |
| 215 | -8.113 | -7.786 | -7.469 | -7.215 | -6.596 | -5.853 | -5.048 | -4.14 | -3.577 | -2.966 |
| 216 | -7.763 | -7.579 | -7.087 | -6.91 | -6.383 | -5.653 | -4.977 | -4.095 | -3.478 | -2.986 |
| 217 | -7.363 | -7.286 | -6.726 | -6.6 | -6.128 | -5.447 | -4.833 | -4.034 | -3.359 | -2.925 |
| 218 | -6.937 | -6.817 | -6.403 | -6.262 | -5.793 | -5.26 | -4.717 | -3.947 | -3.279 | -2.904 |
| 219 | -6.501 | -6.361 | -5.993 | -5.829 | -5.393 | -5.024 | -4.459 | -3.795 | -3.083 | -2.774 |
| 220 | -6.022 | -5.851 | -5.569 | -5.429 | -5.03 | -4.758 | -4.199 | -3.608 | -2.953 | -2.61 |
| 221 | -5.575 | -5.319 | -5.146 | -5.1 | -4.619 | -4.399 | -3.901 | -3.466 | -2.87 | -2.469 |
| 222 | -5.05 | -4.825 | -4.775 | -4.647 | -4.187 | -4.074 | -3.527 | -3.257 | -2.756 | -2.271 |
| 223 | -4.534 | -4.27 | -4.302 | -4.194 | -3.741 | -3.589 | -3.172 | -2.947 | -2.612 | -2.111 |
| 224 | -4.027 | -3.826 | -3.825 | -3.76 | -3.358 | -3.239 | -2.87 | -2.649 | -2.358 | -1.9 |
| 225 | -3.565 | -3.396 | -3.418 | -3.332 | -2.937 | -2.886 | -2.564 | -2.29 | -2.148 | -1.806 |
| 226 | -3.115 | -3.016 | -3.02 | -2.945 | -2.583 | -2.566 | -2.285 | -2.02 | -1.949 | -1.69 |
| 227 | -2.69 | -2.675 | -2.565 | -2.518 | -2.225 | -2.194 | -1.982 | -1.796 | -1.664 | -1.556 |
| 228 | -2.315 | -2.307 | -2.188 | -2.169 | -1.95 | -1.915 | -1.75 | -1.534 | -1.488 | -1.394 |
| 229 | -1.988 | -1.99 | -1.816 | -1.874 | -1.693 | -1.655 | -1.6 | -1.332 | -1.281 | -1.206 |
| 230 | -1.665 | -1.691 | -1.598 | -1.602 | -1.42 | -1.385 | -1.356 | -1.182 | -1.155 | -1.11 |
| 231 | -1.427 | -1.415 | -1.353 | -1.298 | -1.19 | -1.145 | -1.185 | -1.07 | -1.028 | -0.975 |
| 232 | -1.23 | -1.226 | -1.226 | -1.15 | -1.074 | -1.046 | -1.022 | -0.917 | -0.929 | -0.881 |
| 233 | -1.106 | -1.095 | -1.137 | -1.07 | -0.973 | -0.974 | -0.859 | -0.821 | -0.868 | -0.83 |
| 234 | -0.979 | -0.952 | -1.059 | -1 | -0.938 | -0.896 | -0.756 | -0.802 | -0.839 | -0.734 |
| 235 | -0.892 | -0.821 | -0.974 | -0.842 | -0.848 | -0.812 | -0.727 | -0.753 | -0.805 | -0.713 |
| 236 | -0.799 | -0.779 | -0.854 | -0.725 | -0.779 | -0.776 | -0.729 | -0.709 | -0.827 | -0.734 |
| 237 | -0.766 | -0.764 | -0.765 | -0.703 | -0.772 | -0.731 | -0.785 | -0.634 | -0.83 | -0.683 |
| 238 | -0.78 | -0.812 | -0.771 | -0.7 | -0.755 | -0.647 | -0.697 | -0.672 | -0.917 | -0.695 |
| 239 | -0.8 | -0.833 | -0.814 | -0.705 | -0.724 | -0.675 | -0.687 | -0.675 | -0.877 | -0.636 |
| 240 | -0.811 | -0.854 | -0.877 | -0.739 | -0.767 | -0.686 | -0.588 | -0.637 | -0.799 | -0.606 |
| 241 | -0.825 | -0.91 | -0.951 | -0.746 | -0.793 | -0.688 | -0.552 | -0.669 | -0.669 | -0.586 |
| 242 | -0.854 | -0.866 | -0.963 | -0.79 | -0.797 | -0.665 | -0.578 | -0.66 | -0.615 | -0.643 |
| 243 | -0.877 | -0.843 | -0.998 | -0.808 | -0.789 | -0.616 | -0.61 | -0.607 | -0.573 | -0.714 |
| 244 | -0.873 | -0.863 | -0.959 | -0.774 | -0.83 | -0.609 | -0.613 | -0.619 | -0.62 | -0.724 |
| 245 | -0.909 | -0.875 | -0.934 | -0.787 | -0.847 | -0.664 | -0.666 | -0.603 | -0.715 | -0.726 |
| 246 | -0.937 | -0.91 | -0.92 | -0.823 | -0.916 | -0.666 | -0.62 | -0.664 | -0.795 | -0.622 |
| 247 | -0.993 | -0.961 | -0.946 | -0.9 | -0.944 | -0.76 | -0.633 | -0.701 | -0.781 | -0.545 |
| 248 | -1.034 | -1.017 | -0.971 | -0.898 | -1.011 | -0.78 | -0.612 | -0.679 | -0.772 | -0.47 |
| 249 | -1.08 | -1.117 | -1.034 | -0.876 | -1.029 | -0.782 | -0.656 | -0.699 | -0.752 | -0.485 |
| 250 | -1.114 | -1.174 | -1.056 | -0.945 | -0.995 | -0.77 | -0.668 | -0.684 | -0.736 | -0.571 |
| 251 | -1.169 | -1.223 | -1.122 | -1.017 | -1.019 | -0.77 | -0.696 | -0.716 | -0.727 | -0.635 |
| 252 | -1.224 | -1.287 | -1.174 | -1.039 | -1.058 | -0.846 | -0.719 | -0.792 | -0.778 | -0.701 |
| 253 | -1.259 | -1.303 | -1.178 | -1.084 | -1.01 | -0.851 | -0.786 | -0.838 | -0.797 | -0.782 |
| 254 | -1.304 | -1.299 | -1.184 | -1.113 | -0.995 | -0.884 | -0.843 | -0.88 | -0.837 | -0.743 |
| 255 | -1.36 | -1.315 | -1.218 | -1.175 | -1.025 | -0.949 | -0.931 | -0.938 | -0.872 | -0.797 |
| 256 | -1.414 | -1.311 | -1.271 | -1.205 | -1.057 | -0.972 | -0.978 | -0.922 | -0.956 | -0.759 |
| 257 | -1.425 | -1.36 | -1.396 | -1.228 | -1.097 | -1.02 | -1.051 | -0.984 | -1.01 | -0.795 |
| 258 | -1.453 | -1.4 | -1.426 | -1.282 | -1.173 | -1.086 | -1.102 | -1.004 | -1.04 | -0.799 |
| 259 | -1.491 | -1.414 | -1.456 | -1.307 | -1.262 | -1.171 | -1.093 | -1.04 | -1.083 | -0.852 |
| 260 | -1.505 | -1.484 | -1.453 | -1.365 | -1.34 | -1.23 | -1.111 | -1.004 | -1.085 | -0.912 |

(c)-(f) α-helix, β-sheet, β-turn and random coil structures of 10 mg/L γ-PGA at 0, 1, 10, 50 mM NaF ionic strength datasets.

| pH | α-helix (%) | | | |
| --- | --- | --- | --- | --- |
|  | 0 mM NaF | 1 mM NaF | 10 mM NaF | 50 mM NaF |
| 3 | 21.2 | 20.9 | 20.1 | 19.3 |
| 3.4 | 20.4 | 20.4 | 19.5 | 19.2 |
| 3.8 | 20 | 20.1 | 19.1 | 18.1 |
| 4.2 | 19.6 | 19.4 | 18 | 17 |
| 4.6 | 18.5 | 18.2 | 16.4 | 15.3 |
| 5 | 17.3 | 17 | 14.8 | 14 |
| 5.4 | 16.2 | 15.2 | 13.5 | 12.4 |
| 5.8 | 14.6 | 13.4 | 12.3 | 12.1 |
| 6.2 | 13.6 | 12.5 | 11.8 | 11.8 |
| 6.7 | 12.8 | 12 | 11.7 | 11.7 |

| pH | β-sheet (%) | | | |
| --- | --- | --- | --- | --- |
|  | 0 mM NaF | 1 mM NaF | 10 mM NaF | 50 mM NaF |
| 3 | 24.2 | 24.4 | 24.9 | 25.4 |
| 3.4 | 24.6 | 24.7 | 25.1 | 25.2 |
| 3.8 | 24.7 | 24.7 | 25.2 | 25.7 |
| 4.2 | 24.9 | 24.9 | 25.5 | 26 |
| 4.6 | 25.3 | 25.4 | 26.2 | 27 |
| 5 | 25.8 | 25.9 | 27 | 27.5 |
| 5.4 | 26.1 | 26.7 | 27.6 | 28.4 |
| 5.8 | 26.8 | 27.6 | 28.5 | 28.5 |
| 6.2 | 27.5 | 28.1 | 28.8 | 28.8 |
| 6.7 | 27.9 | 28.5 | 28.8 | 28.8 |

| pH | β-turn (%) | | | |
| --- | --- | --- | --- | --- |
|  | 0 mM NaF | 1 mM NaF | 10 mM NaF | 50 mM NaF |
| 3 | 18.9 | 18.9 | 18.8 | 18.9 |
| 3.4 | 18.8 | 18.9 | 18.8 | 18.8 |
| 3.8 | 18.7 | 18.8 | 18.6 | 18.6 |
| 4.2 | 18.6 | 18.6 | 18.3 | 18.2 |
| 4.6 | 18.4 | 18.3 | 18 | 17.9 |
| 5 | 18.1 | 18 | 17.6 | 17.5 |
| 5.4 | 17.7 | 17.6 | 17.3 | 17.2 |
| 5.8 | 17.4 | 17.3 | 17.1 | 17.1 |
| 6.2 | 17.2 | 17.1 | 17 | 17.1 |
| 6.7 | 17.1 | 16.9 | 16.9 | 17 |

| pH | Random coil (%) | | | |
| --- | --- | --- | --- | --- |
|  | 0 mM NaF | 1 mM NaF | 10 mM NaF | 50 mM NaF |
| 3 | 35.7 | 35.7 | 36.3 | 36.5 |
| 3.4 | 36.3 | 36.1 | 36.6 | 36.8 |
| 3.8 | 36.6 | 36.4 | 37.2 | 37.6 |
| 4.2 | 36.9 | 37 | 38.2 | 38.6 |
| 4.6 | 37.9 | 38.1 | 39.3 | 39.9 |
| 5 | 38.9 | 39.2 | 40.5 | 40.9 |
| 5.4 | 39.9 | 40.5 | 41.5 | 42 |
| 5.8 | 41 | 41.7 | 42.1 | 42.2 |
| 6.2 | 41.8 | 42.3 | 42.4 | 42.4 |
| 6.7 | 42.3 | 42.6 | 42.6 | 42.4 |

2. Fig 2 datasets

Current *i* vs Pb^2+^ added for γ-PGA at (a) pH 3.4, (b) 4.2, (c) 5.0 and (d) 6.2 in 10 mM NaNO_3_ datasets.

| Pb^2+^ added (mM) | pH 3.4 | |
| --- | --- | --- |
|  | *i* (nA) | StdDev |
| 0.019 | 30.122 | 0.386 |
| 0.039 | 57.966 | 0.443 |
| 0.058 | 86.816 | 1.031 |
| 0.077 | 113.872 | 1.419 |
| 0.096 | 143.256 | 0.826 |
| 0.114 | 171.166 | 0.387 |
| 0.152 | 227.066 | 1.233 |
| 0.189 | 283.409 | 2.921 |

| Pb^2+^ added (mM) | pH 4.2 | |
| --- | --- | --- |
|  | *i* (nA) | StdDev |
| 0.019 | 22.593 | 0.11 |
| 0.039 | 44.1 | 0.572 |
| 0.058 | 63.09 | 0.637 |
| 0.077 | 83.427 | 0.984 |
| 0.096 | 109.41 | 1.708 |
| 0.114 | 130.97 | 1.995 |
| 0.133 | 157.863 | 3.48 |
| 0.152 | 175.823 | 5.828 |

| Pb^2+^ added (mM) | pH 5.0 | |
| --- | --- | --- |
|  | *i* (nA) | StdDev |
| 0.019 | 10.7 | 0.064 |
| 0.039 | 19.953 | 0.3 |
| 0.058 | 43.757 | 0.626 |
| 0.077 | 74.097 | 0.727 |
| 0.096 | 107.11 | 1.619 |
| 0.114 | 140.94 | 2.445 |
| 0.133 | 173.217 | 3.183 |
| 0.152 | 207.733 | 1.397 |
| 0.17 | 238.46 | 3.746 |
| 0.189 | 275.61 | 4.947 |

| Pb^2+^ added (mM) | pH 6.2 | |
| --- | --- | --- |
|  | *i* (nA) | StdDev |
| 0.019 | 9.633 | 1.334 |
| 0.039 | 16.267 | 0.733 |
| 0.058 | 28.073 | 1.315 |
| 0.077 | 56.02 | 0.825 |
| 0.096 | 86.76 | 0.66 |
| 0.114 | 118.573 | 1.633 |
| 0.133 | 156.96 | 1.59 |
| 0.142 | 169.313 | 1.678 |
| 0.152 | 182.537 | 0.694 |
| 0.161 | 205.863 | 2.641 |

3. Fig 3 datasets

(a) Free Pb^2+^/(added Pb^2+^-free Pb^2+^) vs Free Pb^2+^ datasets.

| pH | Free Pb^2+^ (mM) | Free Pb^2+^/(added Pb^2+^-free Pb^2+^) |
| --- | --- | --- |
| 3.4 | 0.076 | 93.738 |
|  | 0.095 | 577.569 |
|  | 0.114 | 278.489 |
|  | 0.151 | 266.518 |
|  | 0.189 | 2882.893 |
| 4.2 | 0.018 | 10.783 |
|  | 0.034 | 8.556 |
|  | 0.049 | 5.947 |
|  | 0.065 | 5.716 |
|  | 0.086 | 8.538 |
|  | 0.102 | 8.539 |
|  | 0.123 | 12.722 |
|  | 0.137 | 9.609 |
| 5.0 | 0.011 | 0.409 |
|  | 0.024 | 0.74 |
|  | 0.041 | 1.18 |
|  | 0.06 | 1.685 |
|  | 0.079 | 2.223 |
|  | 0.097 | 2.682 |
|  | 0.116 | 3.278 |
|  | 0.133 | 3.625 |
|  | 0.154 | 4.473 |
| 6.2 | 0.009 | 0.325 |
|  | 0.016 | 0.394 |
|  | 0.032 | 0.736 |
|  | 0.05 | 1.113 |
|  | 0.069 | 1.508 |
|  | 0.091 | 2.164 |
|  | 0.098 | 2.22 |
|  | 0.106 | 2.307 |
|  | 0.119 | 2.867 |

(b) Complexed Pb^2+^ (mmol/g dry weight g-PGA) vs Pb^2+^ added (mM) datasets.

| pH | Pb^2+^ added (mM) | Complexed Pb^2+^  (mmol/g dry weight g-PGA) | StdDev |
| --- | --- | --- | --- |
| 4.2 | 0 | 0 | 0.017 |
|  | 0.019 | 0.16 | 0.009 |
|  | 0.039 | 0.4 | 0.045 |
|  | 0.058 | 0.84 | 0.05 |
|  | 0.077 | 1.15 | 0.078 |
|  | 0.096 | 1.01 | 0.135 |
|  | 0.114 | 1.22 | 0.158 |
|  | 0.133 | 0.99 | 0.277 |
|  | 0.152 | 1.46 | 0.465 |
| 5.0 | 0 | 0 | 0.123 |
|  | 0.019 | 1.33 | 0.004 |
|  | 0.039 | 2.75 | 0.017 |
|  | 0.058 | 3.34 | 0.035 |
|  | 0.077 | 3.55 | 0.041 |
|  | 0.096 | 3.6 | 0.092 |
|  | 0.114 | 3.6 | 0.139 |
|  | 0.133 | 3.68 | 0.181 |
|  | 0.152 | 3.62 | 0.08 |
| 6.2 | 0 | 0 | 0.115 |
|  | 0.019 | 1.36 | 0.079 |
|  | 0.039 | 2.9 | 0.044 |
|  | 0.058 | 4.13 | 0.079 |
|  | 0.077 | 4.38 | 0.049 |
|  | 0.096 | 4.46 | 0.04 |
|  | 0.114 | 4.47 | 0.098 |
|  | 0.133 | 4.07 | 0.096 |
|  | 0.142 | 4.28 | 0.101 |
|  | 0.152 | 4.43 | 0.042 |

4. Fig 4 dataset

(a)-(d) α-helix, β-sheet, β-turn and random coil structures of 10 mg/L γ-PGA at 0, 19, 38, 76 μM Pb^2+^ concentrations (10 mM NaF ionic strength) datasets.

| pH | α-helix (%) | | | |
| --- | --- | --- | --- | --- |
|  | 0 μM Pb^2+^ | 19 μM Pb^2+^ | 38 μM Pb^2+^ | 76 μM Pb^2+^ |
| 3 | 20.1 | 18 | 17 | 19.7 |
| 3.4 | 19.5 | 17 | 16.5 | 18.3 |
| 3.8 | 19.1 | 15.7 | 14.6 | 7.4 |
| 4.2 | 18 | 13.1 | 7.5 | 5.4 |
| 4.6 | 16.4 | 9.7 | 6.2 | 5 |
| 5 | 14.8 | 8.6 | 5.7 | 4.8 |
| 5.4 | 13.5 | 7.9 | 5.5 | 4.6 |
| 5.8 | 12.3 | 7.8 | 5.4 | 4.5 |
| 6.2 | 11.8 | 7.8 | 5.3 | 4.5 |
| 6.7 | 11.7 | 7.8 | 5.2 | 4.2 |

| pH | β-sheet (%) | | | |
| --- | --- | --- | --- | --- |
|  | 0 μM Pb^2+^ | 19 μM Pb^2+^ | 38 μM Pb^2+^ | 76 μM Pb^2+^ |
| 3 | 24.9 | 25.8 | 26.5 | 25 |
| 3.4 | 25.1 | 26.2 | 26.6 | 25.7 |
| 3.8 | 25.2 | 26.8 | 27.4 | 32.7 |
| 4.2 | 25.5 | 28.2 | 32.7 | 34.9 |
| 4.6 | 26.2 | 30.5 | 34 | 35.6 |
| 5 | 27 | 31.4 | 34.7 | 35.8 |
| 5.4 | 27.6 | 32 | 34.8 | 36.2 |
| 5.8 | 28.5 | 32.1 | 35 | 36.3 |
| 6.2 | 28.8 | 32 | 35.1 | 36.3 |
| 6.7 | 28.8 | 32 | 35.2 | 36.8 |

| pH | β-turn (%) | | | |
| --- | --- | --- | --- | --- |
|  | 0 μM Pb^2+^ | 19 μM Pb^2+^ | 38 μM Pb^2+^ | 76 μM Pb^2+^ |
| 3 | 18.8 | 18.6 | 18.6 | 18.8 |
| 3.4 | 18.8 | 18.5 | 18.4 | 18.6 |
| 3.8 | 18.6 | 18.2 | 18 | 16.2 |
| 4.2 | 18.3 | 17.6 | 16.3 | 15.6 |
| 4.6 | 18 | 16.8 | 16 | 15.4 |
| 5 | 17.6 | 16.5 | 15.7 | 15.3 |
| 5.4 | 17.3 | 16.3 | 15.6 | 15.2 |
| 5.8 | 17.1 | 16.2 | 15.6 | 15.2 |
| 6.2 | 17 | 16.1 | 15.6 | 15.1 |
| 6.7 | 16.9 | 16.2 | 15.5 | 15.1 |

| pH | Random coil (%) | | | |
| --- | --- | --- | --- | --- |
|  | 0 μM Pb^2+^ | 19 μM Pb^2+^ | 38 μM Pb^2+^ | 76 μM Pb^2+^ |
| 3 | 36.3 | 37.6 | 37.9 | 36.4 |
| 3.4 | 36.6 | 38.2 | 38.4 | 37.4 |
| 3.8 | 37.2 | 39.2 | 39.9 | 43.7 |
| 4.2 | 38.2 | 41 | 43.6 | 44 |
| 4.6 | 39.3 | 42.9 | 43.9 | 44.1 |
| 5 | 40.5 | 43.5 | 44 | 44.1 |
| 5.4 | 41.5 | 43.8 | 44 | 44 |
| 5.8 | 42.1 | 43.9 | 44 | 44.1 |
| 6.2 | 42.4 | 44 | 44 | 44.1 |
| 6.7 | 42.6 | 43.9 | 44 | 43.9 |

5. Fig 5 datasets

(a) Amount of complexed Pb^2+^ with 10 mg/L γ-PGA at pH 4.2, 5.0 and 6.2 (concentrations of Pb^2+^, Ca^2+^ and Mg^2+^ were 38 μM) datasets.

| pH | Complexed Pb^2+^ (mmol/g dry weight γ-PGA) | | | | | |
| --- | --- | --- | --- | --- | --- | --- |
|  | Pb^2+^ | StdDev | Pb^2+^+Ca^2+^ | StdDev | Pb^2+^+Mg^2+^ | StdDev |
| 4.2 | 0.4 | 0.04 | 0.26 | 0.01 | 0.18 | 0.03 |
| 5 | 2.75 | 0.02 | 2.25 | 0.04 | 2.38 | 0.03 |
| 6.2 | 2.9 | 0.04 | 2.37 | 0.02 | 2.89 | 0.04 |

(b)-(d) α-helix, β-sheet, β-turn and random coil structures datasets.

| Structure | pH 4.2 | | | | | |
| --- | --- | --- | --- | --- | --- | --- |
|  | γ-PGA only | Ca^2+^ | Mg^+^ | Pb^2+^ | Pb^2+^+Ca^2+^ | Pb^2+^+Mg^2+^ |
| α-helix | 18 | 16.4 | 16.7 | 7.5 | 7.5 | 7.8 |
| β-sheet | 25.5 | 26.4 | 26.2 | 32.7 | 32.7 | 32.3 |
| β-turn | 18.3 | 18.2 | 18.2 | 16.3 | 16.3 | 16.4 |
| Random coil | 38.2 | 38.8 | 38.9 | 43.6 | 43.6 | 43.5 |

| Structure | pH 5.0 | | | | | |
| --- | --- | --- | --- | --- | --- | --- |
|  | γ-PGA only | Ca^2+^ | Mg^+^ | Pb^2+^ | Pb^2+^+Ca^2+^ | Pb^2+^+Mg^2+^ |
| α-helix | 14.8 | 14.5 | 14 | 5.7 | 5.3 | 5.3 |
| β-sheet | 27 | 27.3 | 27.5 | 34.7 | 35.1 | 35.1 |
| β-turn | 17.6 | 17.6 | 17.6 | 15.7 | 15.5 | 15.5 |
| Random coil | 40.5 | 40.6 | 40.9 | 44 | 44.1 | 44.1 |

| Structure | pH 6.2 | | | | | |
| --- | --- | --- | --- | --- | --- | --- |
|  | γ-PGA only | Ca^2+^ | Mg^+^ | Pb^2+^ | Pb^2+^+Ca^2+^ | Pb^2+^+Mg^2+^ |
| α-helix | 11.8 | 11.3 | 11.5 | 5.3 | 5.1 | 4.9 |
| β-sheet | 28.8 | 29.2 | 29.1 | 35.1 | 35.5 | 35.7 |
| β-turn | 17 | 17 | 17.1 | 15.6 | 15.4 | 15.4 |
| Random coil | 42.4 | 42.5 | 42.4 | 44 | 44 | 44.1 |

6. Fig 6 datasets

(a) FTIR transmittance of γ-PGA/Pb^2+^ complexation, γ-PGA at pH 3.0, 5.0 and 7.0 datasets.

| Wavenumber  (cm^-1^) | γ-PGA | | | γ-PGA/Pb^2+^ complexation |
| --- | --- | --- | --- | --- |
|  | pH 3.0 | pH 5.0 | pH 7.0 |  |
| 2000.231 | 0.98957 | 0.9904 | 0.99915 | 0.99988 |
| 1998.8 | 0.98904 | 0.99007 | 0.9986 | 0.99988 |
| 1997.369 | 0.9885 | 0.98976 | 0.99814 | 0.99986 |
| 1995.938 | 0.98796 | 0.98947 | 0.99781 | 0.99982 |
| 1994.508 | 0.98748 | 0.98919 | 0.99768 | 0.99978 |
| 1993.077 | 0.98711 | 0.98899 | 0.99782 | 0.99975 |
| 1991.646 | 0.98686 | 0.98888 | 0.99822 | 0.99972 |
| 1990.215 | 0.98669 | 0.98884 | 0.99878 | 0.99969 |
| 1988.784 | 0.98655 | 0.9888 | 0.99935 | 0.99976 |
| 1987.354 | 0.9864 | 0.98872 | 0.99977 | 0.99984 |
| 1985.923 | 0.98618 | 0.98856 | 0.99999 | 0.99989 |
| 1984.492 | 0.98589 | 0.98833 | 1 | 0.99992 |
| 1983.061 | 0.98554 | 0.98805 | 0.99976 | 0.99992 |
| 1981.631 | 0.98517 | 0.98774 | 0.99934 | 0.99997 |
| 1980.2 | 0.9848 | 0.98742 | 0.99884 | 1 |
| 1978.769 | 0.98446 | 0.98712 | 0.99835 | 1.00003 |
| 1977.338 | 0.98417 | 0.98685 | 0.99798 | 1.00006 |
| 1975.907 | 0.98397 | 0.98663 | 0.99781 | 1.00008 |
| 1974.477 | 0.98385 | 0.9865 | 0.99789 | 1.00011 |
| 1973.046 | 0.9838 | 0.98644 | 0.9982 | 1.00013 |
| 1971.615 | 0.98381 | 0.98641 | 0.99866 | 1.00016 |
| 1970.184 | 0.98384 | 0.98637 | 0.99915 | 1.0002 |
| 1968.754 | 0.98389 | 0.98627 | 0.99959 | 1.00025 |
| 1967.323 | 0.98393 | 0.98614 | 0.99994 | 1.00029 |
| 1965.892 | 0.98394 | 0.98597 | 1.00012 | 1.00029 |
| 1964.461 | 0.98391 | 0.98572 | 1.00008 | 1.00027 |
| 1963.03 | 0.98385 | 0.98541 | 0.99979 | 1.00028 |
| 1961.6 | 0.98377 | 0.98508 | 0.99933 | 1.0003 |
| 1960.169 | 0.98373 | 0.98477 | 0.99882 | 1.00031 |
| 1958.738 | 0.98375 | 0.98455 | 0.9984 | 1.00029 |
| 1957.307 | 0.98386 | 0.98443 | 0.99814 | 1.00026 |
| 1955.877 | 0.98408 | 0.98442 | 0.99809 | 1.00021 |
| 1954.446 | 0.98438 | 0.98448 | 0.99828 | 1.00013 |
| 1953.015 | 0.98476 | 0.98458 | 0.99864 | 1.00004 |
| 1951.584 | 0.98517 | 0.98464 | 0.99912 | 0.99994 |
| 1950.153 | 0.98557 | 0.98462 | 0.9996 | 0.99986 |
| 1948.723 | 0.98593 | 0.9845 | 0.99997 | 0.9998 |
| 1947.292 | 0.98621 | 0.98427 | 1.00015 | 0.99978 |
| 1945.861 | 0.98641 | 0.98392 | 1.00007 | 0.99977 |
| 1944.43 | 0.98657 | 0.98353 | 0.99977 | 0.99974 |
| 1942.999 | 0.98675 | 0.98319 | 0.99935 | 0.99976 |
| 1941.569 | 0.98698 | 0.98297 | 0.99893 | 0.9997 |
| 1940.138 | 0.98728 | 0.98289 | 0.99857 | 0.99959 |
| 1938.707 | 0.98768 | 0.98289 | 0.99827 | 0.99948 |
| 1937.276 | 0.9882 | 0.98295 | 0.9981 | 0.99939 |
| 1935.846 | 0.98881 | 0.98305 | 0.99809 | 0.9993 |
| 1934.415 | 0.98947 | 0.98314 | 0.99827 | 0.99919 |
| 1932.984 | 0.9901 | 0.98319 | 0.99859 | 0.99923 |
| 1931.553 | 0.99064 | 0.98314 | 0.99899 | 0.99927 |
| 1930.122 | 0.99106 | 0.98299 | 0.99936 | 0.99932 |
| 1928.692 | 0.99134 | 0.98273 | 0.9996 | 0.99937 |
| 1927.261 | 0.99153 | 0.98236 | 0.99964 | 0.99944 |
| 1925.83 | 0.99171 | 0.98194 | 0.99956 | 0.99951 |
| 1924.399 | 0.99198 | 0.98156 | 0.99932 | 0.99954 |
| 1922.969 | 0.99236 | 0.98129 | 0.99901 | 0.99956 |
| 1921.538 | 0.99287 | 0.98117 | 0.9987 | 0.99952 |
| 1920.107 | 0.99348 | 0.98116 | 0.99845 | 0.99949 |
| 1918.676 | 0.99416 | 0.98125 | 0.99832 | 0.99942 |
| 1917.245 | 0.99483 | 0.98137 | 0.99835 | 0.99929 |
| 1915.815 | 0.99536 | 0.98144 | 0.99852 | 0.99912 |
| 1914.384 | 0.9957 | 0.98138 | 0.99875 | 0.99898 |
| 1912.953 | 0.99594 | 0.98118 | 0.99902 | 0.99891 |
| 1911.522 | 0.99615 | 0.98089 | 0.99931 | 0.99889 |
| 1910.092 | 0.99638 | 0.98055 | 0.9996 | 0.99883 |
| 1908.661 | 0.99664 | 0.98021 | 0.99984 | 0.99902 |
| 1907.23 | 0.99693 | 0.97989 | 0.99997 | 0.99915 |
| 1905.799 | 0.99727 | 0.9796 | 0.99996 | 0.99925 |
| 1904.368 | 0.99765 | 0.97936 | 0.99981 | 0.99935 |
| 1902.938 | 0.99805 | 0.97919 | 0.99955 | 0.99944 |
| 1901.507 | 0.99843 | 0.97906 | 0.99927 | 0.99951 |
| 1900.076 | 0.99877 | 0.97895 | 0.99902 | 0.99957 |
| 1898.645 | 0.99905 | 0.97883 | 0.99884 | 0.99964 |
| 1897.215 | 0.99925 | 0.97869 | 0.99877 | 0.99972 |
| 1895.784 | 0.99937 | 0.9785 | 0.99881 | 0.99975 |
| 1894.353 | 0.99942 | 0.97825 | 0.99896 | 0.99976 |
| 1892.922 | 0.99945 | 0.97792 | 0.99916 | 0.99976 |
| 1891.491 | 0.99953 | 0.97752 | 0.99939 | 0.99977 |
| 1890.061 | 0.99968 | 0.97711 | 0.99966 | 0.99978 |
| 1888.63 | 0.99983 | 0.97673 | 0.99992 | 0.99968 |
| 1887.199 | 0.99991 | 0.97635 | 1.00007 | 0.99952 |
| 1885.768 | 0.99994 | 0.97596 | 1.00009 | 0.99938 |
| 1884.337 | 1 | 0.97559 | 0.99999 | 0.99926 |
| 1882.907 | 1.00006 | 0.97525 | 0.99981 | 0.99914 |
| 1881.476 | 1.00008 | 0.97496 | 0.99958 | 0.99898 |
| 1880.045 | 1.00008 | 0.9747 | 0.99935 | 0.99879 |
| 1878.614 | 1.00006 | 0.97446 | 0.99919 | 0.99856 |
| 1877.184 | 1 | 0.9742 | 0.99911 | 0.99829 |
| 1875.753 | 0.99993 | 0.97387 | 0.99911 | 0.99836 |
| 1874.322 | 0.99978 | 0.9734 | 0.99912 | 0.99844 |
| 1872.891 | 0.99958 | 0.97279 | 0.99913 | 0.99858 |
| 1871.46 | 0.99939 | 0.97212 | 0.99921 | 0.9988 |
| 1870.03 | 0.99922 | 0.97149 | 0.99942 | 0.99899 |
| 1868.599 | 0.99899 | 0.97095 | 0.99972 | 0.99916 |
| 1867.168 | 0.99858 | 0.97044 | 0.99997 | 0.99921 |
| 1865.737 | 0.99799 | 0.96991 | 1 | 0.99923 |
| 1864.307 | 0.99731 | 0.96933 | 0.9999 | 0.99926 |
| 1862.876 | 0.99663 | 0.96876 | 0.99968 | 0.99934 |
| 1861.445 | 0.99598 | 0.96827 | 0.99944 | 0.99941 |
| 1860.014 | 0.99528 | 0.96782 | 0.99924 | 0.99942 |
| 1858.583 | 0.99451 | 0.96737 | 0.9991 | 0.99945 |
| 1857.153 | 0.99363 | 0.96686 | 0.99905 | 0.99951 |
| 1855.722 | 0.99261 | 0.96625 | 0.99907 | 0.99956 |
| 1854.291 | 0.99144 | 0.9655 | 0.99915 | 0.9996 |
| 1852.86 | 0.99015 | 0.96461 | 0.99926 | 0.99965 |
| 1851.43 | 0.98877 | 0.9636 | 0.99936 | 0.99971 |
| 1849.999 | 0.98736 | 0.96252 | 0.99942 | 0.9998 |
| 1848.568 | 0.98596 | 0.96146 | 0.99946 | 0.99991 |
| 1847.137 | 0.98459 | 0.96053 | 0.99953 | 1 |
| 1845.706 | 0.98319 | 0.95979 | 0.99964 | 1.00006 |
| 1844.276 | 0.98163 | 0.9592 | 0.99975 | 0.99981 |
| 1842.845 | 0.97982 | 0.95864 | 0.99974 | 0.9993 |
| 1841.414 | 0.97779 | 0.958 | 0.99955 | 0.99878 |
| 1839.983 | 0.9756 | 0.95723 | 0.99926 | 0.99837 |
| 1838.553 | 0.97326 | 0.95634 | 0.99894 | 0.99804 |
| 1837.122 | 0.97078 | 0.95531 | 0.99866 | 0.9977 |
| 1835.691 | 0.96822 | 0.95416 | 0.99844 | 0.99735 |
| 1834.26 | 0.96566 | 0.95293 | 0.9983 | 0.9971 |
| 1832.829 | 0.96315 | 0.95168 | 0.99828 | 0.99695 |
| 1831.399 | 0.96062 | 0.95048 | 0.99843 | 0.99675 |
| 1829.968 | 0.95793 | 0.94932 | 0.99868 | 0.9963 |
| 1828.537 | 0.95497 | 0.94814 | 0.99886 | 0.99553 |
| 1827.106 | 0.95177 | 0.94696 | 0.99886 | 0.99458 |
| 1825.676 | 0.94833 | 0.94583 | 0.99872 | 0.99354 |
| 1824.245 | 0.94467 | 0.94477 | 0.99851 | 0.99243 |
| 1822.814 | 0.94083 | 0.94373 | 0.99823 | 0.99131 |
| 1821.383 | 0.93685 | 0.94262 | 0.99786 | 0.99022 |
| 1819.952 | 0.93274 | 0.94137 | 0.99739 | 0.98914 |
| 1818.522 | 0.92852 | 0.93996 | 0.9969 | 0.98803 |
| 1817.091 | 0.92423 | 0.93844 | 0.99647 | 0.98691 |
| 1815.66 | 0.91992 | 0.93684 | 0.99613 | 0.98582 |
| 1814.229 | 0.91558 | 0.9352 | 0.99589 | 0.98475 |
| 1812.798 | 0.91109 | 0.93355 | 0.99578 | 0.98359 |
| 1811.368 | 0.90631 | 0.93195 | 0.99581 | 0.98222 |
| 1809.937 | 0.90118 | 0.93041 | 0.99592 | 0.98061 |
| 1808.506 | 0.89575 | 0.92893 | 0.99601 | 0.97885 |
| 1807.075 | 0.89009 | 0.92747 | 0.99597 | 0.97705 |
| 1805.645 | 0.88422 | 0.92597 | 0.99574 | 0.97523 |
| 1804.214 | 0.87813 | 0.92441 | 0.99533 | 0.97337 |
| 1802.783 | 0.87177 | 0.9228 | 0.99483 | 0.97142 |
| 1801.352 | 0.86516 | 0.92114 | 0.99431 | 0.96936 |
| 1799.921 | 0.85837 | 0.91941 | 0.99378 | 0.96722 |
| 1798.491 | 0.8515 | 0.91752 | 0.99317 | 0.96508 |
| 1797.06 | 0.84461 | 0.91548 | 0.9925 | 0.96301 |
| 1795.629 | 0.83755 | 0.91337 | 0.9919 | 0.96101 |
| 1794.198 | 0.82992 | 0.91126 | 0.99152 | 0.95886 |
| 1792.768 | 0.82134 | 0.90916 | 0.99138 | 0.95627 |
| 1791.337 | 0.81193 | 0.90709 | 0.99138 | 0.95322 |
| 1789.906 | 0.80228 | 0.90509 | 0.99136 | 0.95005 |
| 1788.475 | 0.79261 | 0.90315 | 0.9912 | 0.94699 |
| 1787.044 | 0.78263 | 0.90119 | 0.99091 | 0.944 |
| 1785.614 | 0.772 | 0.89916 | 0.9905 | 0.94092 |
| 1784.183 | 0.76068 | 0.89704 | 0.98999 | 0.93774 |
| 1782.752 | 0.74876 | 0.89481 | 0.98939 | 0.9345 |
| 1781.321 | 0.73618 | 0.89241 | 0.98865 | 0.93114 |
| 1779.891 | 0.72291 | 0.88984 | 0.98781 | 0.92761 |
| 1778.46 | 0.70921 | 0.88715 | 0.98692 | 0.92403 |
| 1777.029 | 0.69533 | 0.88448 | 0.9861 | 0.92062 |
| 1775.598 | 0.68087 | 0.88189 | 0.9855 | 0.91731 |
| 1774.167 | 0.66483 | 0.87938 | 0.9852 | 0.91373 |
| 1772.737 | 0.64657 | 0.87681 | 0.98507 | 0.90942 |
| 1771.306 | 0.62675 | 0.8741 | 0.98484 | 0.90435 |
| 1769.875 | 0.6068 | 0.87127 | 0.98436 | 0.89892 |
| 1768.444 | 0.58765 | 0.86845 | 0.98373 | 0.89355 |
| 1767.014 | 0.56949 | 0.86571 | 0.9831 | 0.88847 |
| 1765.583 | 0.55226 | 0.86302 | 0.98244 | 0.88364 |
| 1764.152 | 0.53545 | 0.86026 | 0.98164 | 0.87878 |
| 1762.721 | 0.51839 | 0.85731 | 0.98061 | 0.87348 |
| 1761.29 | 0.50119 | 0.85422 | 0.97938 | 0.86768 |
| 1759.86 | 0.48457 | 0.85112 | 0.97804 | 0.86167 |
| 1758.429 | 0.4688 | 0.84814 | 0.97669 | 0.85563 |
| 1756.998 | 0.45371 | 0.8453 | 0.97543 | 0.8495 |
| 1755.567 | 0.43956 | 0.84263 | 0.97427 | 0.84336 |
| 1754.136 | 0.42667 | 0.84011 | 0.9732 | 0.8373 |
| 1752.706 | 0.41478 | 0.83773 | 0.97226 | 0.8311 |
| 1751.275 | 0.40347 | 0.83549 | 0.97155 | 0.82437 |
| 1749.844 | 0.39285 | 0.83344 | 0.97105 | 0.81708 |
| 1748.413 | 0.38341 | 0.83153 | 0.97054 | 0.8096 |
| 1746.983 | 0.37539 | 0.82965 | 0.96975 | 0.80231 |
| 1745.552 | 0.36861 | 0.82777 | 0.96862 | 0.79532 |
| 1744.121 | 0.36279 | 0.826 | 0.96728 | 0.78859 |
| 1742.69 | 0.35775 | 0.82445 | 0.96589 | 0.78199 |
| 1741.259 | 0.35338 | 0.82314 | 0.96447 | 0.77538 |
| 1739.829 | 0.34962 | 0.82194 | 0.96291 | 0.76881 |
| 1738.398 | 0.34652 | 0.82078 | 0.96121 | 0.7626 |
| 1736.967 | 0.34415 | 0.81971 | 0.95951 | 0.75677 |
| 1735.536 | 0.34245 | 0.81887 | 0.95802 | 0.75066 |
| 1734.106 | 0.34137 | 0.81833 | 0.95684 | 0.74364 |
| 1732.675 | 0.34089 | 0.818 | 0.95591 | 0.73613 |
| 1731.244 | 0.34094 | 0.81775 | 0.95509 | 0.72903 |
| 1729.813 | 0.34148 | 0.81752 | 0.95426 | 0.72248 |
| 1728.382 | 0.34254 | 0.81734 | 0.95333 | 0.71616 |
| 1726.952 | 0.34414 | 0.81722 | 0.9522 | 0.70986 |
| 1725.521 | 0.34631 | 0.81715 | 0.95082 | 0.70356 |
| 1724.09 | 0.34904 | 0.81711 | 0.94918 | 0.69732 |
| 1722.659 | 0.35221 | 0.8171 | 0.94738 | 0.69138 |
| 1721.229 | 0.35584 | 0.81713 | 0.94553 | 0.68581 |
| 1719.798 | 0.36036 | 0.8173 | 0.94372 | 0.68019 |
| 1718.367 | 0.36631 | 0.81771 | 0.94199 | 0.67377 |
| 1716.936 | 0.37359 | 0.81826 | 0.94036 | 0.66637 |
| 1715.505 | 0.38137 | 0.81872 | 0.93885 | 0.65865 |
| 1714.075 | 0.38894 | 0.81894 | 0.93747 | 0.65128 |
| 1712.644 | 0.39629 | 0.81893 | 0.93622 | 0.64438 |
| 1711.213 | 0.40379 | 0.81879 | 0.935 | 0.63769 |
| 1709.782 | 0.41182 | 0.81855 | 0.93376 | 0.63099 |
| 1708.352 | 0.4206 | 0.81823 | 0.93243 | 0.62401 |
| 1706.921 | 0.43013 | 0.81774 | 0.93088 | 0.61658 |
| 1705.49 | 0.43987 | 0.817 | 0.92905 | 0.60889 |
| 1704.059 | 0.4491 | 0.81595 | 0.92697 | 0.60135 |
| 1702.628 | 0.45788 | 0.81462 | 0.92474 | 0.59381 |
| 1701.198 | 0.46692 | 0.81301 | 0.92242 | 0.58548 |
| 1699.767 | 0.47599 | 0.81108 | 0.92001 | 0.57608 |
| 1698.336 | 0.48367 | 0.80882 | 0.91756 | 0.56624 |
| 1696.905 | 0.48902 | 0.8062 | 0.91514 | 0.55628 |
| 1695.475 | 0.49214 | 0.80324 | 0.91273 | 0.5461 |
| 1694.044 | 0.49319 | 0.80006 | 0.91034 | 0.53613 |
| 1692.613 | 0.49219 | 0.79679 | 0.90798 | 0.52686 |
| 1691.182 | 0.48926 | 0.79341 | 0.90559 | 0.51804 |
| 1689.751 | 0.48467 | 0.78992 | 0.90311 | 0.50933 |
| 1688.321 | 0.47867 | 0.78645 | 0.90057 | 0.50083 |
| 1686.89 | 0.47091 | 0.78301 | 0.89792 | 0.49233 |
| 1685.459 | 0.46049 | 0.77937 | 0.89493 | 0.48296 |
| 1684.028 | 0.44744 | 0.77542 | 0.89141 | 0.47244 |
| 1682.597 | 0.43369 | 0.77148 | 0.88758 | 0.46206 |
| 1681.167 | 0.42113 | 0.76796 | 0.8839 | 0.45312 |
| 1679.736 | 0.4099 | 0.76495 | 0.88059 | 0.44558 |
| 1678.305 | 0.39911 | 0.7623 | 0.87765 | 0.43863 |
| 1676.874 | 0.388 | 0.75985 | 0.87497 | 0.43154 |
| 1675.444 | 0.37658 | 0.75749 | 0.87246 | 0.42415 |
| 1674.013 | 0.36562 | 0.75523 | 0.8701 | 0.41693 |
| 1672.582 | 0.35578 | 0.75309 | 0.86789 | 0.4103 |
| 1671.151 | 0.34682 | 0.75102 | 0.86572 | 0.40405 |
| 1669.72 | 0.33824 | 0.74891 | 0.86344 | 0.39778 |
| 1668.29 | 0.33028 | 0.74678 | 0.86099 | 0.3917 |
| 1666.859 | 0.32351 | 0.74476 | 0.85849 | 0.38636 |
| 1665.428 | 0.31787 | 0.74295 | 0.85603 | 0.38179 |
| 1663.997 | 0.31281 | 0.74133 | 0.85358 | 0.37753 |
| 1662.567 | 0.30804 | 0.73983 | 0.85106 | 0.3733 |
| 1661.136 | 0.30372 | 0.73841 | 0.84852 | 0.36923 |
| 1659.705 | 0.30004 | 0.73707 | 0.84606 | 0.36564 |
| 1658.274 | 0.29702 | 0.73585 | 0.84381 | 0.36268 |
| 1656.843 | 0.29454 | 0.73482 | 0.8419 | 0.36025 |
| 1655.413 | 0.29239 | 0.73404 | 0.84045 | 0.35797 |
| 1653.982 | 0.29045 | 0.73348 | 0.83939 | 0.35538 |
| 1652.551 | 0.28873 | 0.73286 | 0.83832 | 0.35252 |
| 1651.12 | 0.28737 | 0.73196 | 0.83693 | 0.34993 |
| 1649.69 | 0.28641 | 0.73087 | 0.83528 | 0.34794 |
| 1648.259 | 0.28585 | 0.72979 | 0.83358 | 0.34626 |
| 1646.828 | 0.28567 | 0.7288 | 0.83182 | 0.34454 |
| 1645.397 | 0.28578 | 0.72787 | 0.8299 | 0.34276 |
| 1643.966 | 0.28609 | 0.72699 | 0.82786 | 0.34116 |
| 1642.536 | 0.28658 | 0.72625 | 0.82586 | 0.3399 |
| 1641.105 | 0.28733 | 0.72573 | 0.82407 | 0.33899 |
| 1639.674 | 0.28846 | 0.72556 | 0.82265 | 0.33844 |
| 1638.243 | 0.2902 | 0.72585 | 0.82178 | 0.33822 |
| 1636.813 | 0.29282 | 0.72663 | 0.8215 | 0.33826 |
| 1635.382 | 0.29622 | 0.72769 | 0.82161 | 0.33842 |
| 1633.951 | 0.29983 | 0.72862 | 0.82174 | 0.3386 |
| 1632.52 | 0.30324 | 0.72927 | 0.8218 | 0.33887 |
| 1631.089 | 0.30671 | 0.72989 | 0.822 | 0.33944 |
| 1629.659 | 0.31077 | 0.73076 | 0.82255 | 0.34047 |
| 1628.228 | 0.31558 | 0.73194 | 0.82342 | 0.34196 |
| 1626.797 | 0.32102 | 0.73334 | 0.82443 | 0.34379 |
| 1625.366 | 0.32702 | 0.73496 | 0.82553 | 0.34593 |
| 1623.936 | 0.33361 | 0.73684 | 0.82675 | 0.3484 |
| 1622.505 | 0.34051 | 0.73885 | 0.828 | 0.35103 |
| 1621.074 | 0.34723 | 0.74076 | 0.82918 | 0.35364 |
| 1619.643 | 0.35392 | 0.74259 | 0.83043 | 0.35628 |
| 1618.212 | 0.36147 | 0.7446 | 0.83211 | 0.35925 |
| 1616.782 | 0.37034 | 0.74682 | 0.83428 | 0.36258 |
| 1615.351 | 0.37969 | 0.74884 | 0.83648 | 0.36583 |
| 1613.92 | 0.38846 | 0.75031 | 0.83827 | 0.36855 |
| 1612.489 | 0.39667 | 0.75128 | 0.83969 | 0.3708 |
| 1611.058 | 0.40506 | 0.75201 | 0.84093 | 0.37282 |
| 1609.628 | 0.41406 | 0.7526 | 0.84204 | 0.37465 |
| 1608.197 | 0.42348 | 0.75309 | 0.84291 | 0.37617 |
| 1606.766 | 0.43295 | 0.7536 | 0.84353 | 0.37727 |
| 1605.335 | 0.44228 | 0.7543 | 0.84404 | 0.37794 |
| 1603.905 | 0.45145 | 0.75531 | 0.84457 | 0.37821 |
| 1602.474 | 0.46042 | 0.75658 | 0.8451 | 0.37801 |
| 1601.043 | 0.46901 | 0.75793 | 0.84557 | 0.3773 |
| 1599.612 | 0.47702 | 0.75923 | 0.84598 | 0.37606 |
| 1598.181 | 0.4843 | 0.76045 | 0.84642 | 0.37435 |
| 1596.751 | 0.49076 | 0.76163 | 0.84702 | 0.37222 |
| 1595.32 | 0.49627 | 0.76281 | 0.84785 | 0.36971 |
| 1593.889 | 0.5006 | 0.76402 | 0.84886 | 0.3669 |
| 1592.458 | 0.50357 | 0.7653 | 0.84995 | 0.36386 |
| 1591.028 | 0.50508 | 0.76671 | 0.85104 | 0.36067 |
| 1589.597 | 0.50511 | 0.76832 | 0.8521 | 0.3574 |
| 1588.166 | 0.50367 | 0.77018 | 0.85307 | 0.35409 |
| 1586.735 | 0.50079 | 0.7723 | 0.85392 | 0.35075 |
| 1585.304 | 0.49654 | 0.77462 | 0.85466 | 0.34742 |
| 1583.874 | 0.49107 | 0.7771 | 0.85534 | 0.34413 |
| 1582.443 | 0.48465 | 0.77961 | 0.85604 | 0.34095 |
| 1581.012 | 0.47761 | 0.78203 | 0.85684 | 0.33797 |
| 1579.581 | 0.47 | 0.78436 | 0.85796 | 0.33521 |
| 1578.151 | 0.46143 | 0.78673 | 0.85971 | 0.33247 |
| 1576.72 | 0.45158 | 0.78912 | 0.86218 | 0.32958 |
| 1575.289 | 0.44118 | 0.79115 | 0.8649 | 0.32661 |
| 1573.858 | 0.43146 | 0.79245 | 0.86725 | 0.3239 |
| 1572.427 | 0.42269 | 0.79315 | 0.86926 | 0.32156 |
| 1570.997 | 0.41404 | 0.79367 | 0.87134 | 0.3194 |
| 1569.566 | 0.405 | 0.79419 | 0.8736 | 0.31723 |
| 1568.135 | 0.39606 | 0.79458 | 0.87563 | 0.31506 |
| 1566.704 | 0.38791 | 0.7947 | 0.87709 | 0.31305 |
| 1565.274 | 0.38073 | 0.79455 | 0.87798 | 0.31127 |
| 1563.843 | 0.37443 | 0.79423 | 0.87848 | 0.30976 |
| 1562.412 | 0.36881 | 0.79385 | 0.87891 | 0.30853 |
| 1560.981 | 0.36333 | 0.79354 | 0.87985 | 0.3075 |
| 1559.55 | 0.3574 | 0.7933 | 0.88168 | 0.30644 |
| 1558.12 | 0.35139 | 0.79288 | 0.88391 | 0.30517 |
| 1556.689 | 0.34627 | 0.79202 | 0.88562 | 0.30384 |
| 1555.258 | 0.34233 | 0.79084 | 0.88665 | 0.30264 |
| 1553.827 | 0.33918 | 0.78962 | 0.88743 | 0.30165 |
| 1552.396 | 0.33652 | 0.78857 | 0.88826 | 0.30087 |
| 1550.966 | 0.33428 | 0.78781 | 0.88912 | 0.30026 |
| 1549.535 | 0.33247 | 0.78733 | 0.88993 | 0.2998 |
| 1548.104 | 0.33109 | 0.78707 | 0.89052 | 0.29948 |
| 1546.673 | 0.33013 | 0.78695 | 0.89084 | 0.29933 |
| 1545.243 | 0.32963 | 0.78701 | 0.8911 | 0.2994 |
| 1543.812 | 0.32969 | 0.78738 | 0.89167 | 0.29971 |
| 1542.381 | 0.33052 | 0.78811 | 0.89289 | 0.3003 |
| 1540.95 | 0.33226 | 0.789 | 0.89467 | 0.30111 |
| 1539.519 | 0.33464 | 0.78962 | 0.89642 | 0.30191 |
| 1538.089 | 0.33703 | 0.78966 | 0.89757 | 0.30252 |
| 1536.658 | 0.33935 | 0.78936 | 0.89822 | 0.30305 |
| 1535.227 | 0.34222 | 0.78923 | 0.89896 | 0.30383 |
| 1533.796 | 0.34621 | 0.78959 | 0.90014 | 0.30502 |
| 1532.366 | 0.3511 | 0.79035 | 0.90155 | 0.30648 |
| 1530.935 | 0.35636 | 0.79127 | 0.90272 | 0.30799 |
| 1529.504 | 0.36194 | 0.79227 | 0.9035 | 0.30954 |
| 1528.073 | 0.36831 | 0.79346 | 0.9041 | 0.31126 |
| 1526.642 | 0.37576 | 0.79493 | 0.90472 | 0.31324 |
| 1525.212 | 0.38418 | 0.79658 | 0.90544 | 0.3154 |
| 1523.781 | 0.39373 | 0.79836 | 0.90629 | 0.31777 |
| 1522.35 | 0.40494 | 0.80026 | 0.90733 | 0.32044 |
| 1520.919 | 0.41774 | 0.80224 | 0.90855 | 0.32333 |
| 1519.489 | 0.43133 | 0.80422 | 0.90988 | 0.32623 |
| 1518.058 | 0.44547 | 0.80625 | 0.91131 | 0.32908 |
| 1516.627 | 0.46049 | 0.80851 | 0.91286 | 0.33197 |
| 1515.196 | 0.47627 | 0.811 | 0.91443 | 0.33491 |
| 1513.765 | 0.49225 | 0.81358 | 0.9158 | 0.33782 |
| 1512.335 | 0.5082 | 0.8162 | 0.91683 | 0.34071 |
| 1510.904 | 0.52468 | 0.81901 | 0.91766 | 0.34377 |
| 1509.473 | 0.54351 | 0.82262 | 0.91877 | 0.34741 |
| 1508.042 | 0.56725 | 0.82781 | 0.92068 | 0.35215 |
| 1506.612 | 0.59573 | 0.83444 | 0.92328 | 0.35785 |
| 1505.181 | 0.6238 | 0.84098 | 0.92561 | 0.36339 |
| 1503.75 | 0.6469 | 0.84617 | 0.92702 | 0.36796 |
| 1502.319 | 0.66607 | 0.85043 | 0.92789 | 0.37197 |
| 1500.888 | 0.68511 | 0.85498 | 0.92899 | 0.37638 |
| 1499.458 | 0.70653 | 0.86066 | 0.93081 | 0.38184 |
| 1498.027 | 0.73037 | 0.86763 | 0.93335 | 0.38842 |
| 1496.596 | 0.75465 | 0.87533 | 0.9362 | 0.39563 |
| 1495.165 | 0.77673 | 0.88285 | 0.93881 | 0.40271 |
| 1493.735 | 0.79546 | 0.88975 | 0.9409 | 0.40937 |
| 1492.304 | 0.81216 | 0.89651 | 0.94267 | 0.41618 |
| 1490.873 | 0.82884 | 0.90398 | 0.94458 | 0.42402 |
| 1489.442 | 0.84583 | 0.91235 | 0.94679 | 0.43304 |
| 1488.011 | 0.86155 | 0.92083 | 0.94905 | 0.44252 |
| 1486.581 | 0.87453 | 0.92854 | 0.95097 | 0.45171 |
| 1485.15 | 0.88465 | 0.93518 | 0.9525 | 0.46051 |
| 1483.719 | 0.89261 | 0.94101 | 0.95383 | 0.46937 |
| 1482.288 | 0.89896 | 0.94634 | 0.95518 | 0.47868 |
| 1480.857 | 0.90377 | 0.95118 | 0.95659 | 0.48842 |
| 1479.427 | 0.90686 | 0.95537 | 0.95797 | 0.49832 |
| 1477.996 | 0.90805 | 0.95877 | 0.9592 | 0.50828 |
| 1476.565 | 0.90716 | 0.96142 | 0.96029 | 0.51854 |
| 1475.134 | 0.90368 | 0.96332 | 0.96128 | 0.52943 |
| 1473.704 | 0.8968 | 0.96433 | 0.96215 | 0.54102 |
| 1472.273 | 0.88628 | 0.96418 | 0.96268 | 0.55259 |
| 1470.842 | 0.87291 | 0.96268 | 0.96259 | 0.56282 |
| 1469.411 | 0.85745 | 0.95977 | 0.96176 | 0.571 |
| 1467.98 | 0.83924 | 0.95537 | 0.96027 | 0.57752 |
| 1466.55 | 0.81666 | 0.94922 | 0.95819 | 0.5829 |
| 1465.119 | 0.78946 | 0.94138 | 0.95557 | 0.58691 |
| 1463.688 | 0.76032 | 0.93258 | 0.95255 | 0.58888 |
| 1462.257 | 0.73266 | 0.92384 | 0.94935 | 0.58854 |
| 1460.827 | 0.70736 | 0.91555 | 0.94618 | 0.58595 |
| 1459.396 | 0.68258 | 0.90732 | 0.94313 | 0.5809 |
| 1457.965 | 0.65709 | 0.8989 | 0.94029 | 0.57309 |
| 1456.534 | 0.63321 | 0.89104 | 0.93787 | 0.56345 |
| 1455.103 | 0.61488 | 0.88492 | 0.93601 | 0.55415 |
| 1453.673 | 0.60325 | 0.88087 | 0.93472 | 0.54665 |
| 1452.242 | 0.5966 | 0.87832 | 0.93385 | 0.5409 |
| 1450.811 | 0.59302 | 0.87665 | 0.9333 | 0.53647 |
| 1449.38 | 0.5916 | 0.87559 | 0.93307 | 0.53319 |
| 1447.95 | 0.59187 | 0.87505 | 0.93312 | 0.5311 |
| 1446.519 | 0.59334 | 0.87489 | 0.93339 | 0.53021 |
| 1445.088 | 0.59547 | 0.87496 | 0.93377 | 0.53044 |
| 1443.657 | 0.5979 | 0.87516 | 0.9342 | 0.53164 |
| 1442.226 | 0.60044 | 0.87541 | 0.93464 | 0.53361 |
| 1440.796 | 0.60294 | 0.87564 | 0.93505 | 0.53621 |
| 1439.365 | 0.60537 | 0.87582 | 0.93549 | 0.53941 |
| 1437.934 | 0.60768 | 0.87591 | 0.93604 | 0.54322 |
| 1436.503 | 0.60961 | 0.87577 | 0.9366 | 0.54722 |
| 1435.073 | 0.61066 | 0.87513 | 0.93686 | 0.55059 |
| 1433.642 | 0.61046 | 0.87371 | 0.93644 | 0.55252 |
| 1432.211 | 0.60882 | 0.87133 | 0.93519 | 0.55263 |
| 1430.78 | 0.60563 | 0.86793 | 0.93309 | 0.5506 |
| 1429.349 | 0.6008 | 0.86362 | 0.9302 | 0.54618 |
| 1427.919 | 0.59451 | 0.85866 | 0.92667 | 0.53943 |
| 1426.488 | 0.5872 | 0.85333 | 0.92267 | 0.53062 |
| 1425.057 | 0.57937 | 0.84783 | 0.91837 | 0.52017 |
| 1423.626 | 0.57161 | 0.84239 | 0.91402 | 0.5087 |
| 1422.195 | 0.5644 | 0.83716 | 0.90976 | 0.4968 |
| 1420.765 | 0.55792 | 0.83209 | 0.90561 | 0.48449 |
| 1419.334 | 0.55217 | 0.8271 | 0.90158 | 0.47172 |
| 1417.903 | 0.54731 | 0.82239 | 0.89785 | 0.45927 |
| 1416.472 | 0.5435 | 0.81823 | 0.89457 | 0.44825 |
| 1415.042 | 0.54057 | 0.81457 | 0.89162 | 0.4389 |
| 1413.611 | 0.53812 | 0.81116 | 0.88878 | 0.4307 |
| 1412.18 | 0.53581 | 0.80783 | 0.88591 | 0.42317 |
| 1410.749 | 0.53349 | 0.80464 | 0.88306 | 0.41626 |
| 1409.318 | 0.5311 | 0.80171 | 0.88034 | 0.4101 |
| 1407.888 | 0.52864 | 0.79917 | 0.87786 | 0.40476 |
| 1406.457 | 0.52612 | 0.79709 | 0.87574 | 0.4002 |
| 1405.026 | 0.52362 | 0.79558 | 0.87412 | 0.39633 |
| 1403.595 | 0.52128 | 0.79472 | 0.87313 | 0.39324 |
| 1402.165 | 0.51921 | 0.79456 | 0.87282 | 0.39097 |
| 1400.734 | 0.51743 | 0.79513 | 0.87325 | 0.38951 |
| 1399.303 | 0.51587 | 0.79648 | 0.87452 | 0.38879 |
| 1397.872 | 0.51437 | 0.79859 | 0.87665 | 0.38877 |
| 1396.441 | 0.51261 | 0.80139 | 0.8796 | 0.38941 |
| 1395.011 | 0.51013 | 0.80474 | 0.8832 | 0.39064 |
| 1393.58 | 0.50622 | 0.80825 | 0.88699 | 0.39228 |
| 1392.149 | 0.49932 | 0.81138 | 0.89031 | 0.39403 |
| 1390.718 | 0.48624 | 0.81365 | 0.8926 | 0.39566 |
| 1389.288 | 0.46294 | 0.81483 | 0.8936 | 0.39702 |
| 1387.857 | 0.42952 | 0.815 | 0.89341 | 0.39808 |
| 1386.426 | 0.39533 | 0.8149 | 0.8929 | 0.39913 |
| 1384.995 | 0.37496 | 0.81579 | 0.8935 | 0.40082 |
| 1383.564 | 0.37795 | 0.81868 | 0.8964 | 0.40376 |
| 1382.134 | 0.40324 | 0.82353 | 0.90152 | 0.40795 |
| 1380.703 | 0.44042 | 0.82927 | 0.90769 | 0.41276 |
| 1379.272 | 0.47613 | 0.83464 | 0.91351 | 0.41742 |
| 1377.841 | 0.50256 | 0.83898 | 0.9183 | 0.42159 |
| 1376.411 | 0.52015 | 0.8424 | 0.92221 | 0.42545 |
| 1374.98 | 0.53299 | 0.84529 | 0.92566 | 0.42933 |
| 1373.549 | 0.54388 | 0.84789 | 0.92886 | 0.43336 |
| 1372.118 | 0.55344 | 0.85019 | 0.93169 | 0.43746 |
| 1370.687 | 0.56151 | 0.85215 | 0.9341 | 0.44162 |
| 1369.257 | 0.56812 | 0.85383 | 0.93612 | 0.44591 |
| 1367.826 | 0.5734 | 0.8553 | 0.93785 | 0.45033 |
| 1366.395 | 0.57742 | 0.8566 | 0.93931 | 0.45486 |
| 1364.964 | 0.58032 | 0.85779 | 0.94061 | 0.45955 |
| 1363.534 | 0.58237 | 0.859 | 0.94196 | 0.46451 |
| 1362.103 | 0.5837 | 0.86031 | 0.94351 | 0.46967 |
| 1360.672 | 0.58432 | 0.86158 | 0.94518 | 0.47473 |
| 1359.241 | 0.58422 | 0.86263 | 0.94672 | 0.47945 |
| 1357.81 | 0.58349 | 0.86332 | 0.94799 | 0.48382 |
| 1356.38 | 0.58229 | 0.86368 | 0.94895 | 0.48788 |
| 1354.949 | 0.58086 | 0.86379 | 0.9496 | 0.49157 |
| 1353.518 | 0.57948 | 0.86371 | 0.94992 | 0.49475 |
| 1352.087 | 0.57848 | 0.86354 | 0.94991 | 0.49733 |
| 1350.656 | 0.5781 | 0.86334 | 0.94961 | 0.49928 |
| 1349.226 | 0.57845 | 0.86322 | 0.94912 | 0.50064 |
| 1347.795 | 0.5795 | 0.86325 | 0.94856 | 0.50151 |
| 1346.364 | 0.58116 | 0.8635 | 0.94808 | 0.50205 |
| 1344.933 | 0.5833 | 0.86397 | 0.94776 | 0.50245 |
| 1343.503 | 0.58583 | 0.86462 | 0.94764 | 0.50291 |
| 1342.072 | 0.58874 | 0.86545 | 0.94777 | 0.50365 |
| 1340.641 | 0.59205 | 0.86655 | 0.94828 | 0.50482 |
| 1339.21 | 0.59573 | 0.868 | 0.94927 | 0.50653 |
| 1337.779 | 0.59961 | 0.86975 | 0.95067 | 0.50875 |
| 1336.349 | 0.60354 | 0.87162 | 0.95215 | 0.51137 |
| 1334.918 | 0.60748 | 0.8734 | 0.95337 | 0.51428 |
| 1333.487 | 0.61151 | 0.87507 | 0.95416 | 0.5174 |
| 1332.056 | 0.61568 | 0.87668 | 0.95451 | 0.52061 |
| 1330.626 | 0.61996 | 0.87831 | 0.95451 | 0.52375 |
| 1329.195 | 0.62428 | 0.88001 | 0.95424 | 0.52662 |
| 1327.764 | 0.62857 | 0.88173 | 0.95378 | 0.52904 |
| 1326.333 | 0.63273 | 0.88344 | 0.95321 | 0.53088 |
| 1324.902 | 0.63671 | 0.8851 | 0.95262 | 0.53208 |
| 1323.472 | 0.64045 | 0.88665 | 0.95208 | 0.53267 |
| 1322.041 | 0.6439 | 0.88804 | 0.95168 | 0.53268 |
| 1320.61 | 0.64702 | 0.88926 | 0.95147 | 0.53219 |
| 1319.179 | 0.64974 | 0.89033 | 0.95149 | 0.53132 |
| 1317.749 | 0.65191 | 0.89124 | 0.9517 | 0.53019 |
| 1316.318 | 0.65337 | 0.89199 | 0.95201 | 0.52895 |
| 1314.887 | 0.654 | 0.89263 | 0.95233 | 0.52776 |
| 1313.456 | 0.65371 | 0.8932 | 0.95261 | 0.52673 |
| 1312.025 | 0.65247 | 0.89378 | 0.95288 | 0.52596 |
| 1310.595 | 0.6503 | 0.89437 | 0.95313 | 0.52554 |
| 1309.164 | 0.64727 | 0.89499 | 0.95334 | 0.5255 |
| 1307.733 | 0.64351 | 0.89561 | 0.95355 | 0.52586 |
| 1306.302 | 0.6392 | 0.8962 | 0.95384 | 0.5266 |
| 1304.872 | 0.63455 | 0.8967 | 0.95428 | 0.52766 |
| 1303.441 | 0.62979 | 0.89703 | 0.9549 | 0.52893 |
| 1302.01 | 0.62511 | 0.89712 | 0.95569 | 0.53032 |
| 1300.579 | 0.62064 | 0.89694 | 0.9566 | 0.53172 |
| 1299.148 | 0.61651 | 0.89651 | 0.95758 | 0.53308 |
| 1297.718 | 0.61281 | 0.89589 | 0.95853 | 0.53433 |
| 1296.287 | 0.6096 | 0.89517 | 0.95937 | 0.53543 |
| 1294.856 | 0.60692 | 0.89446 | 0.96001 | 0.53635 |
| 1293.425 | 0.60478 | 0.89385 | 0.96038 | 0.53708 |
| 1291.995 | 0.60315 | 0.89344 | 0.96045 | 0.53765 |
| 1290.564 | 0.60194 | 0.89326 | 0.96026 | 0.53808 |
| 1289.133 | 0.60104 | 0.89333 | 0.95994 | 0.5384 |
| 1287.702 | 0.60032 | 0.8936 | 0.95961 | 0.53861 |
| 1286.271 | 0.59964 | 0.89395 | 0.95939 | 0.53872 |
| 1284.841 | 0.59886 | 0.89426 | 0.9593 | 0.53874 |
| 1283.41 | 0.59791 | 0.89442 | 0.9594 | 0.53871 |
| 1281.979 | 0.59676 | 0.89438 | 0.95969 | 0.53869 |
| 1280.548 | 0.59541 | 0.89413 | 0.96016 | 0.5387 |
| 1279.117 | 0.59392 | 0.89371 | 0.96071 | 0.53875 |
| 1277.687 | 0.59232 | 0.89317 | 0.96124 | 0.53882 |
| 1276.256 | 0.59062 | 0.89259 | 0.96162 | 0.53888 |
| 1274.825 | 0.58887 | 0.89205 | 0.96173 | 0.53892 |
| 1273.394 | 0.58709 | 0.89162 | 0.96156 | 0.53894 |
| 1271.964 | 0.58528 | 0.89132 | 0.96113 | 0.53893 |
| 1270.533 | 0.58344 | 0.89114 | 0.96056 | 0.5389 |
| 1269.102 | 0.58152 | 0.89101 | 0.95998 | 0.53883 |
| 1267.671 | 0.57947 | 0.89085 | 0.95954 | 0.53869 |
| 1266.24 | 0.57722 | 0.8906 | 0.95936 | 0.53849 |
| 1264.81 | 0.57472 | 0.89019 | 0.95951 | 0.53822 |
| 1263.379 | 0.57192 | 0.8896 | 0.96006 | 0.53796 |
| 1261.948 | 0.56879 | 0.88888 | 0.96102 | 0.53789 |
| 1260.517 | 0.56531 | 0.88808 | 0.96237 | 0.53825 |
| 1259.087 | 0.56149 | 0.88731 | 0.96398 | 0.53927 |
| 1257.656 | 0.55733 | 0.88661 | 0.96564 | 0.54106 |
| 1256.225 | 0.55285 | 0.88599 | 0.96716 | 0.5436 |
| 1254.794 | 0.54809 | 0.88543 | 0.96839 | 0.54678 |
| 1253.363 | 0.54306 | 0.88492 | 0.96926 | 0.55044 |
| 1251.933 | 0.53783 | 0.88443 | 0.96979 | 0.55443 |
| 1250.502 | 0.53248 | 0.88391 | 0.97006 | 0.5586 |
| 1249.071 | 0.52711 | 0.88331 | 0.97019 | 0.56284 |
| 1247.64 | 0.52181 | 0.88257 | 0.97033 | 0.56707 |
| 1246.21 | 0.51665 | 0.88165 | 0.97057 | 0.57122 |
| 1244.779 | 0.51169 | 0.88055 | 0.97097 | 0.57523 |
| 1243.348 | 0.50695 | 0.87928 | 0.97152 | 0.57905 |
| 1241.917 | 0.50248 | 0.87792 | 0.97221 | 0.58263 |
| 1240.486 | 0.49825 | 0.87655 | 0.97295 | 0.58593 |
| 1239.056 | 0.49423 | 0.87526 | 0.97362 | 0.58894 |
| 1237.625 | 0.49041 | 0.87409 | 0.97411 | 0.59169 |
| 1236.194 | 0.48677 | 0.87308 | 0.97433 | 0.5942 |
| 1234.763 | 0.48332 | 0.87223 | 0.97423 | 0.59651 |
| 1233.333 | 0.48011 | 0.87156 | 0.97386 | 0.59867 |
| 1231.902 | 0.4772 | 0.87104 | 0.97332 | 0.60074 |
| 1230.471 | 0.47468 | 0.87063 | 0.97275 | 0.60278 |
| 1229.04 | 0.47259 | 0.87026 | 0.97231 | 0.60485 |
| 1227.609 | 0.471 | 0.8699 | 0.97214 | 0.607 |
| 1226.179 | 0.46994 | 0.86955 | 0.97234 | 0.6093 |
| 1224.748 | 0.46945 | 0.86925 | 0.97295 | 0.6118 |
| 1223.317 | 0.46951 | 0.86905 | 0.97393 | 0.6145 |
| 1221.886 | 0.4701 | 0.86899 | 0.97517 | 0.61737 |
| 1220.455 | 0.47118 | 0.86911 | 0.97655 | 0.62038 |
| 1219.025 | 0.47273 | 0.86946 | 0.9779 | 0.6235 |
| 1217.594 | 0.47472 | 0.87007 | 0.97906 | 0.62667 |
| 1216.163 | 0.47716 | 0.87092 | 0.9799 | 0.62979 |
| 1214.732 | 0.48002 | 0.872 | 0.98036 | 0.6328 |
| 1213.302 | 0.48329 | 0.87324 | 0.98048 | 0.63565 |
| 1211.871 | 0.48698 | 0.87458 | 0.98038 | 0.63835 |
| 1210.44 | 0.49113 | 0.87594 | 0.98022 | 0.64099 |
| 1209.009 | 0.49578 | 0.8773 | 0.98019 | 0.64366 |
| 1207.578 | 0.50097 | 0.8787 | 0.98044 | 0.64649 |
| 1206.148 | 0.50669 | 0.8802 | 0.98105 | 0.64958 |
| 1204.717 | 0.51287 | 0.88185 | 0.98202 | 0.65297 |
| 1203.286 | 0.51943 | 0.88365 | 0.98328 | 0.65663 |
| 1201.855 | 0.52622 | 0.88562 | 0.98472 | 0.6605 |
| 1200.425 | 0.53302 | 0.8877 | 0.98618 | 0.66442 |
| 1198.994 | 0.53961 | 0.88984 | 0.98749 | 0.66823 |
| 1197.563 | 0.5458 | 0.89196 | 0.98849 | 0.67176 |
| 1196.132 | 0.55145 | 0.89399 | 0.98904 | 0.67486 |
| 1194.701 | 0.5565 | 0.89583 | 0.98911 | 0.67743 |
| 1193.271 | 0.56095 | 0.89741 | 0.98879 | 0.67941 |
| 1191.84 | 0.56483 | 0.89869 | 0.9882 | 0.68079 |
| 1190.409 | 0.56829 | 0.89962 | 0.98751 | 0.68163 |
| 1188.978 | 0.57147 | 0.90027 | 0.9869 | 0.68202 |
| 1187.548 | 0.57454 | 0.9007 | 0.98647 | 0.68208 |
| 1186.117 | 0.57768 | 0.90104 | 0.98632 | 0.68197 |
| 1184.686 | 0.58105 | 0.90136 | 0.98645 | 0.68179 |
| 1183.255 | 0.58477 | 0.90175 | 0.98682 | 0.68165 |
| 1181.824 | 0.58897 | 0.90226 | 0.98733 | 0.68165 |
| 1180.394 | 0.59373 | 0.90293 | 0.98788 | 0.68184 |
| 1178.963 | 0.59912 | 0.90381 | 0.98835 | 0.68227 |
| 1177.532 | 0.60517 | 0.9049 | 0.98865 | 0.68297 |
| 1176.101 | 0.61196 | 0.9062 | 0.98871 | 0.68399 |
| 1174.671 | 0.61958 | 0.90768 | 0.98854 | 0.68535 |
| 1173.24 | 0.62813 | 0.90929 | 0.98819 | 0.68708 |
| 1171.809 | 0.6377 | 0.91101 | 0.98778 | 0.6892 |
| 1170.378 | 0.64838 | 0.91285 | 0.98745 | 0.69178 |
| 1168.947 | 0.66023 | 0.91485 | 0.98734 | 0.6949 |
| 1167.517 | 0.67332 | 0.91708 | 0.98759 | 0.69866 |
| 1166.086 | 0.68768 | 0.91961 | 0.98827 | 0.70315 |
| 1164.655 | 0.70328 | 0.9225 | 0.98937 | 0.70847 |
| 1163.224 | 0.72002 | 0.92578 | 0.99084 | 0.71465 |
| 1161.794 | 0.73773 | 0.92947 | 0.99256 | 0.72168 |
| 1160.363 | 0.75614 | 0.93354 | 0.99439 | 0.72945 |
| 1158.932 | 0.77494 | 0.9379 | 0.99614 | 0.73781 |
| 1157.501 | 0.79368 | 0.94242 | 0.99768 | 0.74652 |
| 1156.07 | 0.8118 | 0.94694 | 0.99889 | 0.75534 |
| 1154.64 | 0.82864 | 0.95123 | 0.99967 | 0.76401 |
| 1153.209 | 0.84345 | 0.95506 | 1 | 0.77226 |
| 1151.778 | 0.85542 | 0.95821 | 1 | 0.7798 |
| 1150.347 | 0.8638 | 0.96048 | 0.99975 | 0.78633 |
| 1148.916 | 0.86798 | 0.96173 | 0.99932 | 0.79159 |
| 1147.486 | 0.86763 | 0.96192 | 0.99872 | 0.79535 |
| 1146.055 | 0.86275 | 0.96108 | 0.99792 | 0.79738 |
| 1144.624 | 0.85378 | 0.95934 | 0.99691 | 0.79756 |
| 1143.193 | 0.84151 | 0.95687 | 0.99567 | 0.79585 |
| 1141.763 | 0.82706 | 0.95385 | 0.9942 | 0.79237 |
| 1140.332 | 0.8117 | 0.95045 | 0.99243 | 0.78734 |
| 1138.901 | 0.79659 | 0.94684 | 0.99031 | 0.78112 |
| 1137.47 | 0.78267 | 0.94314 | 0.9878 | 0.77409 |
| 1136.039 | 0.77064 | 0.93942 | 0.98497 | 0.76664 |
| 1134.609 | 0.76091 | 0.93575 | 0.98188 | 0.75913 |
| 1133.178 | 0.75365 | 0.93219 | 0.97864 | 0.75188 |
| 1131.747 | 0.74881 | 0.92876 | 0.97537 | 0.74514 |
| 1130.316 | 0.74626 | 0.92554 | 0.97229 | 0.73917 |
| 1128.886 | 0.74579 | 0.92268 | 0.96964 | 0.73419 |
| 1127.455 | 0.74709 | 0.92026 | 0.96753 | 0.73027 |
| 1126.024 | 0.7498 | 0.91826 | 0.9659 | 0.72731 |
| 1124.593 | 0.75354 | 0.91662 | 0.96462 | 0.72514 |
| 1123.162 | 0.75804 | 0.91528 | 0.96358 | 0.72366 |
| 1121.732 | 0.76315 | 0.91423 | 0.96273 | 0.72283 |
| 1120.301 | 0.76885 | 0.91346 | 0.96199 | 0.7226 |
| 1118.87 | 0.77514 | 0.91293 | 0.96124 | 0.72291 |
| 1117.439 | 0.78207 | 0.91262 | 0.96042 | 0.72371 |
| 1116.009 | 0.78973 | 0.91255 | 0.95954 | 0.725 |
| 1114.578 | 0.79828 | 0.9128 | 0.95873 | 0.72683 |
| 1113.147 | 0.80787 | 0.91344 | 0.95812 | 0.72929 |
| 1111.716 | 0.81851 | 0.91457 | 0.95784 | 0.73238 |
| 1110.285 | 0.83015 | 0.91621 | 0.95798 | 0.73608 |
| 1108.855 | 0.84262 | 0.91836 | 0.95861 | 0.7403 |
| 1107.424 | 0.85568 | 0.92101 | 0.95976 | 0.74499 |
| 1105.993 | 0.86904 | 0.92408 | 0.96141 | 0.75013 |
| 1104.562 | 0.88234 | 0.92746 | 0.96344 | 0.75563 |
| 1103.132 | 0.89514 | 0.93097 | 0.96566 | 0.76137 |
| 1101.701 | 0.90697 | 0.93438 | 0.96786 | 0.7672 |
| 1100.27 | 0.91737 | 0.93745 | 0.96982 | 0.77294 |
| 1098.839 | 0.92591 | 0.94002 | 0.97134 | 0.77834 |
| 1097.408 | 0.93231 | 0.94199 | 0.97232 | 0.78319 |
| 1095.978 | 0.93648 | 0.94337 | 0.97273 | 0.78733 |
| 1094.547 | 0.93853 | 0.9442 | 0.97263 | 0.79065 |
| 1093.116 | 0.93879 | 0.94458 | 0.97215 | 0.79314 |
| 1091.685 | 0.93768 | 0.94467 | 0.97148 | 0.79486 |
| 1090.254 | 0.9357 | 0.94462 | 0.97082 | 0.79593 |
| 1088.824 | 0.93329 | 0.94453 | 0.97033 | 0.79652 |
| 1087.393 | 0.93082 | 0.94443 | 0.97011 | 0.79679 |
| 1085.962 | 0.92854 | 0.94428 | 0.97017 | 0.79686 |
| 1084.531 | 0.92657 | 0.94404 | 0.97046 | 0.79681 |
| 1083.101 | 0.92504 | 0.94371 | 0.97093 | 0.7967 |
| 1081.67 | 0.92402 | 0.94327 | 0.97144 | 0.79657 |
| 1080.239 | 0.92357 | 0.94268 | 0.97184 | 0.79645 |
| 1078.808 | 0.92364 | 0.94191 | 0.97195 | 0.79636 |
| 1077.377 | 0.92423 | 0.94101 | 0.97165 | 0.79635 |
| 1075.947 | 0.92533 | 0.94008 | 0.97091 | 0.7965 |
| 1074.516 | 0.92689 | 0.93923 | 0.96979 | 0.79685 |
| 1073.085 | 0.92885 | 0.93852 | 0.96842 | 0.79744 |
| 1071.654 | 0.93106 | 0.93798 | 0.96701 | 0.79826 |
| 1070.224 | 0.93335 | 0.93758 | 0.96571 | 0.79931 |
| 1068.793 | 0.93553 | 0.93725 | 0.96461 | 0.80058 |
| 1067.362 | 0.93743 | 0.93692 | 0.96373 | 0.80203 |
| 1065.931 | 0.93892 | 0.9365 | 0.96307 | 0.80363 |
| 1064.5 | 0.93987 | 0.93591 | 0.9626 | 0.80532 |
| 1063.07 | 0.94018 | 0.93507 | 0.96219 | 0.80706 |
| 1061.639 | 0.93975 | 0.93392 | 0.96166 | 0.80885 |
| 1060.208 | 0.93855 | 0.93248 | 0.96084 | 0.81069 |
| 1058.777 | 0.93658 | 0.93079 | 0.95958 | 0.81258 |
| 1057.347 | 0.93383 | 0.92892 | 0.95783 | 0.81446 |
| 1055.916 | 0.93026 | 0.92694 | 0.9556 | 0.81622 |
| 1054.485 | 0.92591 | 0.92493 | 0.95303 | 0.81779 |
| 1053.054 | 0.92087 | 0.92296 | 0.95035 | 0.81916 |
| 1051.623 | 0.91529 | 0.92104 | 0.94776 | 0.82033 |
| 1050.193 | 0.90939 | 0.9192 | 0.94547 | 0.82126 |
| 1048.762 | 0.90339 | 0.91748 | 0.94364 | 0.82189 |
| 1047.331 | 0.89751 | 0.91592 | 0.94239 | 0.82214 |
| 1045.9 | 0.89194 | 0.91453 | 0.94176 | 0.82203 |
| 1044.47 | 0.88683 | 0.91335 | 0.94172 | 0.82158 |
| 1043.039 | 0.88229 | 0.9124 | 0.94214 | 0.82085 |
| 1041.608 | 0.87838 | 0.91173 | 0.94285 | 0.81991 |
| 1040.177 | 0.87507 | 0.91136 | 0.94365 | 0.81887 |
| 1038.746 | 0.87232 | 0.91128 | 0.94438 | 0.81784 |
| 1037.316 | 0.87006 | 0.91148 | 0.94497 | 0.8169 |
| 1035.885 | 0.86829 | 0.91194 | 0.94545 | 0.81611 |
| 1034.454 | 0.86698 | 0.91265 | 0.94589 | 0.81553 |
| 1033.023 | 0.86616 | 0.91361 | 0.94644 | 0.81519 |
| 1031.593 | 0.86582 | 0.91481 | 0.94728 | 0.81514 |
| 1030.162 | 0.86602 | 0.91626 | 0.94855 | 0.81545 |
| 1028.731 | 0.86682 | 0.91796 | 0.95039 | 0.81619 |
| 1027.3 | 0.86839 | 0.91993 | 0.95286 | 0.81746 |
| 1025.869 | 0.87089 | 0.92217 | 0.95591 | 0.81935 |
| 1024.439 | 0.87453 | 0.92468 | 0.95943 | 0.82196 |
| 1023.008 | 0.87944 | 0.92748 | 0.96323 | 0.82538 |
| 1021.577 | 0.88571 | 0.93054 | 0.96709 | 0.82963 |
| 1020.146 | 0.89334 | 0.93381 | 0.97077 | 0.83474 |
| 1018.715 | 0.90217 | 0.93722 | 0.97404 | 0.84068 |
| 1017.285 | 0.91192 | 0.94074 | 0.97674 | 0.84737 |
| 1015.854 | 0.92217 | 0.9443 | 0.97882 | 0.85464 |
| 1014.423 | 0.93242 | 0.94784 | 0.98034 | 0.86224 |
| 1012.992 | 0.94211 | 0.95128 | 0.98146 | 0.86989 |
| 1011.562 | 0.95074 | 0.95449 | 0.98234 | 0.87736 |
| 1010.131 | 0.95786 | 0.95737 | 0.98314 | 0.8844 |
| 1008.7 | 0.96325 | 0.95979 | 0.98395 | 0.89078 |
| 1007.269 | 0.96678 | 0.96162 | 0.9848 | 0.89633 |
| 1005.838 | 0.96846 | 0.96271 | 0.98561 | 0.90097 |
| 1004.408 | 0.96834 | 0.96302 | 0.98626 | 0.90465 |
| 1002.977 | 0.96665 | 0.96255 | 0.98659 | 0.90734 |
| 1001.546 | 0.96369 | 0.9614 | 0.98643 | 0.90901 |
| 1000.115 | 0.9598 | 0.95972 | 0.98566 | 0.90973 |

(b) ATR-FTIR absorbance of γ-PGA, γ-PGA/Ca^2+^ complexation at different Ca^2+^:Glutamate ratios datasets.

| Wavenumber  (cm^-1^) | γ-PGA | Ca^2+^:Glutamate | | |
| --- | --- | --- | --- | --- |
|  |  | 1:2 | 1:1 | 2:1 |
| 2000.201 | -0.00095 | -0.00193 | -0.00258 | -0.00446 |
| 1998.77 | -0.00095 | -0.00192 | -0.00259 | -0.00444 |
| 1997.34 | -0.00093 | -0.00191 | -0.0026 | -0.00442 |
| 1995.909 | -0.00089 | -0.00189 | -0.00259 | -0.00439 |
| 1994.478 | -0.00084 | -0.00183 | -0.00254 | -0.00434 |
| 1993.047 | -0.00079 | -0.00177 | -0.0025 | -0.00428 |
| 1991.616 | -0.00078 | -0.00175 | -0.00251 | -0.00425 |
| 1990.186 | -0.00081 | -0.00178 | -0.00256 | -0.00427 |
| 1988.755 | -0.00087 | -0.00182 | -0.00261 | -0.00432 |
| 1987.324 | -0.00092 | -0.00186 | -0.00264 | -0.00437 |
| 1985.893 | -0.00096 | -0.00189 | -0.00265 | -0.00442 |
| 1984.463 | -0.00097 | -0.00191 | -0.00266 | -0.00444 |
| 1983.032 | -0.00095 | -0.00191 | -0.00266 | -0.00443 |
| 1981.601 | -0.00094 | -0.00188 | -0.00264 | -0.00441 |
| 1980.17 | -0.00093 | -0.00186 | -0.00261 | -0.0044 |
| 1978.74 | -0.00092 | -0.00187 | -0.00259 | -0.00439 |
| 1977.309 | -0.00091 | -0.00189 | -0.0026 | -0.0044 |
| 1975.878 | -0.00093 | -0.00193 | -0.00261 | -0.00441 |
| 1974.447 | -0.00096 | -0.00196 | -0.00264 | -0.00444 |
| 1973.017 | -0.00098 | -0.00198 | -0.00266 | -0.00446 |
| 1971.586 | -0.00099 | -0.00198 | -0.00265 | -0.00446 |
| 1970.155 | -0.00096 | -0.00194 | -0.0026 | -0.00443 |
| 1968.724 | -0.00089 | -0.00186 | -0.00252 | -0.00438 |
| 1967.294 | -0.00084 | -0.00179 | -0.00248 | -0.00435 |
| 1965.863 | -0.00083 | -0.00178 | -0.00248 | -0.00436 |
| 1964.432 | -0.00085 | -0.0018 | -0.00252 | -0.00439 |
| 1963.001 | -0.00088 | -0.00182 | -0.00254 | -0.00441 |
| 1961.571 | -0.0009 | -0.00185 | -0.00256 | -0.00441 |
| 1960.14 | -0.00094 | -0.00189 | -0.0026 | -0.00443 |
| 1958.709 | -0.00097 | -0.00193 | -0.00263 | -0.00445 |
| 1957.278 | -0.00097 | -0.00195 | -0.00265 | -0.00445 |
| 1955.847 | -0.00095 | -0.00194 | -0.00265 | -0.00446 |
| 1954.417 | -0.00095 | -0.00194 | -0.00268 | -0.00447 |
| 1952.986 | -0.00097 | -0.00197 | -0.00272 | -0.00449 |
| 1951.555 | -0.00099 | -0.00198 | -0.00273 | -0.0045 |
| 1950.124 | -0.00097 | -0.00196 | -0.00269 | -0.00448 |
| 1948.694 | -0.00093 | -0.00192 | -0.00263 | -0.00444 |
| 1947.263 | -0.00087 | -0.00188 | -0.00257 | -0.00439 |
| 1945.832 | -0.00079 | -0.00181 | -0.00248 | -0.00431 |
| 1944.401 | -0.0007 | -0.00172 | -0.00237 | -0.00423 |
| 1942.971 | -0.00064 | -0.00166 | -0.00229 | -0.0042 |
| 1941.54 | -0.00068 | -0.00169 | -0.00233 | -0.00427 |
| 1940.109 | -0.0008 | -0.00179 | -0.00248 | -0.0044 |
| 1938.678 | -0.00091 | -0.00188 | -0.00263 | -0.00451 |
| 1937.248 | -0.00097 | -0.00192 | -0.00272 | -0.00456 |
| 1935.817 | -0.00098 | -0.00193 | -0.00276 | -0.00457 |
| 1934.386 | -0.00097 | -0.00194 | -0.00275 | -0.00458 |
| 1932.955 | -0.00098 | -0.00195 | -0.00273 | -0.00457 |
| 1931.525 | -0.00098 | -0.00194 | -0.00272 | -0.00457 |
| 1930.094 | -0.00099 | -0.00195 | -0.00272 | -0.00456 |
| 1928.663 | -0.00102 | -0.00197 | -0.00272 | -0.00457 |
| 1927.232 | -0.00103 | -0.00198 | -0.00271 | -0.00458 |
| 1925.802 | -0.00097 | -0.00195 | -0.00263 | -0.00454 |
| 1924.371 | -0.00085 | -0.00186 | -0.0025 | -0.00447 |
| 1922.94 | -0.00076 | -0.00177 | -0.00241 | -0.0044 |
| 1921.509 | -0.00074 | -0.00175 | -0.0024 | -0.00439 |
| 1920.079 | -0.00077 | -0.00175 | -0.00241 | -0.00442 |
| 1918.648 | -0.00079 | -0.00175 | -0.00242 | -0.00443 |
| 1917.217 | -0.00081 | -0.00178 | -0.00246 | -0.00445 |
| 1915.786 | -0.00086 | -0.00185 | -0.00256 | -0.0045 |
| 1914.355 | -0.00093 | -0.00193 | -0.00266 | -0.00456 |
| 1912.925 | -0.00096 | -0.00196 | -0.00269 | -0.00457 |
| 1911.494 | -0.00094 | -0.00193 | -0.00265 | -0.00452 |
| 1910.063 | -0.0009 | -0.00188 | -0.0026 | -0.00446 |
| 1908.632 | -0.00091 | -0.00187 | -0.00261 | -0.00446 |
| 1907.202 | -0.00097 | -0.00192 | -0.00267 | -0.00453 |
| 1905.771 | -0.00104 | -0.00199 | -0.00272 | -0.00462 |
| 1904.34 | -0.00107 | -0.00202 | -0.00272 | -0.00469 |
| 1902.909 | -0.00106 | -0.00202 | -0.0027 | -0.00473 |
| 1901.479 | -0.00105 | -0.00202 | -0.0027 | -0.00474 |
| 1900.048 | -0.00105 | -0.00203 | -0.00272 | -0.00473 |
| 1898.617 | -0.00104 | -0.00201 | -0.00272 | -0.00469 |
| 1897.186 | -0.00101 | -0.00198 | -0.00268 | -0.00463 |
| 1895.756 | -0.00099 | -0.00196 | -0.00264 | -0.00459 |
| 1894.325 | -0.00099 | -0.00197 | -0.00265 | -0.0046 |
| 1892.894 | -0.00099 | -0.00197 | -0.00269 | -0.00462 |
| 1891.463 | -0.00096 | -0.00193 | -0.00269 | -0.00461 |
| 1890.033 | -0.00091 | -0.00187 | -0.00266 | -0.0046 |
| 1888.602 | -0.00092 | -0.00188 | -0.00266 | -0.00463 |
| 1887.171 | -0.001 | -0.00196 | -0.00273 | -0.00471 |
| 1885.74 | -0.00109 | -0.00204 | -0.0028 | -0.00479 |
| 1884.31 | -0.00115 | -0.0021 | -0.00283 | -0.00483 |
| 1882.879 | -0.00118 | -0.00213 | -0.00285 | -0.00485 |
| 1881.448 | -0.00119 | -0.00214 | -0.00285 | -0.00486 |
| 1880.017 | -0.0012 | -0.00215 | -0.00286 | -0.00485 |
| 1878.587 | -0.0012 | -0.00215 | -0.00287 | -0.00484 |
| 1877.156 | -0.00119 | -0.00214 | -0.00289 | -0.00483 |
| 1875.725 | -0.00119 | -0.00213 | -0.0029 | -0.00483 |
| 1874.294 | -0.00116 | -0.00211 | -0.00289 | -0.00483 |
| 1872.863 | -0.00109 | -0.00203 | -0.00281 | -0.00478 |
| 1871.433 | -0.00093 | -0.00187 | -0.00265 | -0.00463 |
| 1870.002 | -0.00075 | -0.00165 | -0.00244 | -0.00443 |
| 1868.571 | -0.00066 | -0.00152 | -0.00232 | -0.00434 |
| 1867.14 | -0.00077 | -0.00162 | -0.00239 | -0.00445 |
| 1865.71 | -0.00098 | -0.00184 | -0.00258 | -0.00466 |
| 1864.279 | -0.00113 | -0.00202 | -0.00276 | -0.00482 |
| 1862.848 | -0.00118 | -0.0021 | -0.00285 | -0.00487 |
| 1861.417 | -0.00116 | -0.0021 | -0.00287 | -0.00486 |
| 1859.987 | -0.00113 | -0.00208 | -0.00285 | -0.00485 |
| 1858.556 | -0.00112 | -0.00209 | -0.00285 | -0.00485 |
| 1857.125 | -0.00113 | -0.00212 | -0.00287 | -0.00485 |
| 1855.694 | -0.00117 | -0.00216 | -0.00292 | -0.00487 |
| 1854.264 | -0.00121 | -0.0022 | -0.00298 | -0.00492 |
| 1852.833 | -0.00124 | -0.00223 | -0.00302 | -0.00495 |
| 1851.402 | -0.00123 | -0.0022 | -0.00299 | -0.00496 |
| 1849.971 | -0.00117 | -0.00212 | -0.0029 | -0.0049 |
| 1848.541 | -0.00106 | -0.00198 | -0.00277 | -0.00479 |
| 1847.11 | -0.00093 | -0.00181 | -0.00264 | -0.00466 |
| 1845.679 | -0.0008 | -0.00166 | -0.00252 | -0.00454 |
| 1844.248 | -0.00073 | -0.00159 | -0.00247 | -0.0045 |
| 1842.818 | -0.0008 | -0.00168 | -0.00257 | -0.00459 |
| 1841.387 | -0.00096 | -0.00185 | -0.00275 | -0.00474 |
| 1839.956 | -0.00108 | -0.00199 | -0.00289 | -0.00485 |
| 1838.525 | -0.00114 | -0.00205 | -0.00294 | -0.00489 |
| 1837.095 | -0.00115 | -0.00207 | -0.00293 | -0.00488 |
| 1835.664 | -0.00118 | -0.00208 | -0.00292 | -0.00486 |
| 1834.233 | -0.00122 | -0.00209 | -0.00291 | -0.00485 |
| 1832.802 | -0.00119 | -0.00202 | -0.00284 | -0.0048 |
| 1831.371 | -0.0011 | -0.00187 | -0.00272 | -0.00469 |
| 1829.941 | -0.001 | -0.00174 | -0.00261 | -0.00461 |
| 1828.51 | -0.00098 | -0.00174 | -0.00258 | -0.00461 |
| 1827.079 | -0.00099 | -0.0018 | -0.00261 | -0.00467 |
| 1825.648 | -0.00101 | -0.00187 | -0.00267 | -0.00473 |
| 1824.218 | -0.00107 | -0.00194 | -0.00276 | -0.00481 |
| 1822.787 | -0.00121 | -0.00206 | -0.00289 | -0.00495 |
| 1821.356 | -0.00133 | -0.00218 | -0.00299 | -0.00507 |
| 1819.925 | -0.00138 | -0.00223 | -0.00303 | -0.00511 |
| 1818.495 | -0.00136 | -0.00223 | -0.00303 | -0.00508 |
| 1817.064 | -0.00134 | -0.00222 | -0.00304 | -0.00504 |
| 1815.633 | -0.00132 | -0.00222 | -0.00305 | -0.00502 |
| 1814.202 | -0.00128 | -0.00219 | -0.003 | -0.00497 |
| 1812.772 | -0.00119 | -0.0021 | -0.00289 | -0.00489 |
| 1811.341 | -0.00111 | -0.002 | -0.00279 | -0.0048 |
| 1809.91 | -0.0011 | -0.00196 | -0.00278 | -0.0048 |
| 1808.479 | -0.00118 | -0.00201 | -0.00286 | -0.00489 |
| 1807.049 | -0.00127 | -0.0021 | -0.00294 | -0.005 |
| 1805.618 | -0.0013 | -0.00213 | -0.00295 | -0.00505 |
| 1804.187 | -0.00122 | -0.00204 | -0.00285 | -0.00498 |
| 1802.756 | -0.00106 | -0.00187 | -0.00269 | -0.00483 |
| 1801.326 | -0.00094 | -0.00173 | -0.00259 | -0.00473 |
| 1799.895 | -0.00094 | -0.00172 | -0.00261 | -0.00474 |
| 1798.464 | -0.00101 | -0.0018 | -0.00269 | -0.0048 |
| 1797.033 | -0.00105 | -0.00186 | -0.00275 | -0.00482 |
| 1795.602 | -0.00105 | -0.00185 | -0.00275 | -0.00479 |
| 1794.172 | -0.001 | -0.00175 | -0.00266 | -0.00469 |
| 1792.741 | -0.00093 | -0.00161 | -0.00254 | -0.00457 |
| 1791.31 | -0.00093 | -0.0016 | -0.00252 | -0.00458 |
| 1789.879 | -0.00106 | -0.00179 | -0.00267 | -0.00475 |
| 1788.449 | -0.0012 | -0.00201 | -0.00287 | -0.00495 |
| 1787.018 | -0.00125 | -0.00212 | -0.00297 | -0.00507 |
| 1785.587 | -0.00122 | -0.00212 | -0.00295 | -0.00511 |
| 1784.156 | -0.00119 | -0.00208 | -0.0029 | -0.00511 |
| 1782.726 | -0.00118 | -0.00205 | -0.00287 | -0.00511 |
| 1781.295 | -0.00118 | -0.00202 | -0.00287 | -0.00509 |
| 1779.864 | -0.00119 | -0.00201 | -0.00292 | -0.00508 |
| 1778.433 | -0.00124 | -0.00203 | -0.00299 | -0.0051 |
| 1777.003 | -0.00128 | -0.00204 | -0.00301 | -0.00511 |
| 1775.572 | -0.00126 | -0.00197 | -0.00293 | -0.00506 |
| 1774.141 | -0.00119 | -0.0018 | -0.00278 | -0.00495 |
| 1772.71 | -0.00111 | -0.00162 | -0.00264 | -0.00484 |
| 1771.28 | -0.00104 | -0.00156 | -0.0026 | -0.00477 |
| 1769.849 | -0.00101 | -0.00165 | -0.00265 | -0.0048 |
| 1768.418 | -0.00105 | -0.00181 | -0.00277 | -0.00492 |
| 1766.987 | -0.00118 | -0.002 | -0.00294 | -0.0051 |
| 1765.557 | -0.00132 | -0.00213 | -0.00308 | -0.00525 |
| 1764.126 | -0.00136 | -0.00215 | -0.00312 | -0.00528 |
| 1762.695 | -0.0013 | -0.00206 | -0.00306 | -0.0052 |
| 1761.264 | -0.00123 | -0.00199 | -0.003 | -0.00513 |
| 1759.834 | -0.00122 | -0.002 | -0.00301 | -0.00512 |
| 1758.403 | -0.00125 | -0.00204 | -0.00305 | -0.00514 |
| 1756.972 | -0.0013 | -0.00208 | -0.00311 | -0.00517 |
| 1755.541 | -0.00136 | -0.00211 | -0.00316 | -0.0052 |
| 1754.11 | -0.00136 | -0.00206 | -0.00312 | -0.00518 |
| 1752.68 | -0.0012 | -0.00188 | -0.0029 | -0.00503 |
| 1751.249 | -0.00094 | -0.00161 | -0.00261 | -0.00482 |
| 1749.818 | -0.00077 | -0.00143 | -0.00247 | -0.00474 |
| 1748.387 | -0.00081 | -0.00146 | -0.00258 | -0.00483 |
| 1746.957 | -0.00095 | -0.00163 | -0.00278 | -0.00499 |
| 1745.526 | -0.00108 | -0.00179 | -0.00292 | -0.00511 |
| 1744.095 | -0.0012 | -0.00191 | -0.00301 | -0.0052 |
| 1742.664 | -0.00133 | -0.002 | -0.00312 | -0.00529 |
| 1741.234 | -0.00147 | -0.00209 | -0.00324 | -0.0054 |
| 1739.803 | -0.00158 | -0.00216 | -0.00334 | -0.0055 |
| 1738.372 | -0.00163 | -0.0022 | -0.00336 | -0.00554 |
| 1736.941 | -0.00157 | -0.00214 | -0.00327 | -0.00548 |
| 1735.511 | -0.00135 | -0.00192 | -0.00305 | -0.00528 |
| 1734.08 | -0.00105 | -0.00167 | -0.00284 | -0.00505 |
| 1732.649 | -0.00094 | -0.00169 | -0.00288 | -0.00505 |
| 1731.218 | -0.00109 | -0.00196 | -0.00312 | -0.00527 |
| 1729.788 | -0.00135 | -0.00225 | -0.00339 | -0.00552 |
| 1728.357 | -0.00161 | -0.00248 | -0.00362 | -0.00573 |
| 1726.926 | -0.00181 | -0.00263 | -0.00379 | -0.00587 |
| 1725.495 | -0.0019 | -0.00267 | -0.00384 | -0.00592 |
| 1724.065 | -0.00191 | -0.00265 | -0.0038 | -0.00589 |
| 1722.634 | -0.0019 | -0.00259 | -0.00372 | -0.00584 |
| 1721.203 | -0.00185 | -0.00249 | -0.00359 | -0.00576 |
| 1719.772 | -0.00169 | -0.00226 | -0.00334 | -0.00558 |
| 1718.342 | -0.00139 | -0.00188 | -0.00295 | -0.00525 |
| 1716.911 | -0.00111 | -0.00155 | -0.00266 | -0.00493 |
| 1715.48 | -0.00112 | -0.00159 | -0.00273 | -0.00488 |
| 1714.049 | -0.00137 | -0.0019 | -0.00307 | -0.00508 |
| 1712.618 | -0.00168 | -0.00221 | -0.0034 | -0.0053 |
| 1711.188 | -0.00192 | -0.00238 | -0.00362 | -0.0054 |
| 1709.757 | -0.00203 | -0.00237 | -0.00367 | -0.00535 |
| 1708.326 | -0.00198 | -0.00223 | -0.00357 | -0.00518 |
| 1706.895 | -0.00179 | -0.00199 | -0.0033 | -0.00494 |
| 1705.465 | -0.00156 | -0.00175 | -0.00299 | -0.00468 |
| 1704.034 | -0.00144 | -0.00158 | -0.00274 | -0.00446 |
| 1702.603 | -0.00144 | -0.00142 | -0.00254 | -0.0043 |
| 1701.172 | -0.00143 | -0.0012 | -0.00233 | -0.0041 |
| 1699.742 | -0.00133 | -0.00099 | -0.00214 | -0.00382 |
| 1698.311 | -0.00121 | -0.00094 | -0.0021 | -0.00355 |
| 1696.88 | -0.0012 | -0.00105 | -0.00221 | -0.00341 |
| 1695.449 | -0.00136 | -0.00125 | -0.00236 | -0.00343 |
| 1694.019 | -0.00154 | -0.00143 | -0.00241 | -0.00349 |
| 1692.588 | -0.00158 | -0.00148 | -0.00229 | -0.00343 |
| 1691.157 | -0.00145 | -0.00137 | -0.00201 | -0.00319 |
| 1689.726 | -0.00123 | -0.00115 | -0.00169 | -0.00283 |
| 1688.296 | -0.00103 | -0.0009 | -0.00146 | -0.0025 |
| 1686.865 | -0.00085 | -0.00061 | -0.00126 | -0.00223 |
| 1685.434 | -0.00058 | -0.00015 | -0.0009 | -0.00182 |
| 1684.003 | -0.00015 | 0.00044 | -0.0004 | -0.00113 |
| 1682.573 | 0.00022 | 0.00076 | -0.00015 | -0.00044 |
| 1681.142 | 0.00038 | 0.00074 | -0.00015 | -0.00001 |
| 1679.711 | 0.00044 | 0.00065 | -0.00008 | 0.00028 |
| 1678.28 | 0.00048 | 0.00069 | 0.00015 | 0.00054 |
| 1676.849 | 0.0006 | 0.00094 | 0.00051 | 0.00086 |
| 1675.419 | 0.00088 | 0.00141 | 0.00092 | 0.0013 |
| 1673.988 | 0.00127 | 0.00192 | 0.00128 | 0.00184 |
| 1672.557 | 0.00166 | 0.00235 | 0.0016 | 0.0024 |
| 1671.126 | 0.00212 | 0.00281 | 0.00204 | 0.00296 |
| 1669.696 | 0.00276 | 0.00338 | 0.00272 | 0.00358 |
| 1668.265 | 0.00342 | 0.00384 | 0.00345 | 0.00417 |
| 1666.834 | 0.00387 | 0.00401 | 0.00391 | 0.00462 |
| 1665.403 | 0.00421 | 0.0042 | 0.00421 | 0.00507 |
| 1663.973 | 0.00469 | 0.00475 | 0.00467 | 0.00578 |
| 1662.542 | 0.00546 | 0.00569 | 0.00548 | 0.00689 |
| 1661.111 | 0.00642 | 0.00659 | 0.00638 | 0.00818 |
| 1659.68 | 0.00727 | 0.00712 | 0.00696 | 0.00933 |
| 1658.25 | 0.00788 | 0.00741 | 0.00719 | 0.01023 |
| 1656.819 | 0.00834 | 0.00765 | 0.00738 | 0.01086 |
| 1655.388 | 0.00883 | 0.00798 | 0.00787 | 0.01126 |
| 1653.957 | 0.00959 | 0.00857 | 0.00896 | 0.01152 |
| 1652.527 | 0.01053 | 0.00965 | 0.01033 | 0.01189 |
| 1651.096 | 0.01133 | 0.01091 | 0.0112 | 0.0125 |
| 1649.665 | 0.01215 | 0.01204 | 0.01188 | 0.01327 |
| 1648.234 | 0.0132 | 0.01305 | 0.01293 | 0.01429 |
| 1646.804 | 0.01425 | 0.01375 | 0.01434 | 0.01566 |
| 1645.373 | 0.01486 | 0.01404 | 0.01538 | 0.01702 |
| 1643.942 | 0.01512 | 0.01425 | 0.01576 | 0.01802 |
| 1642.511 | 0.01543 | 0.01464 | 0.01604 | 0.0188 |
| 1641.081 | 0.01599 | 0.0152 | 0.01668 | 0.01952 |
| 1639.65 | 0.01679 | 0.01583 | 0.01772 | 0.02013 |
| 1638.219 | 0.01777 | 0.01648 | 0.01893 | 0.02072 |
| 1636.788 | 0.01888 | 0.01733 | 0.01997 | 0.02155 |
| 1635.357 | 0.01978 | 0.01846 | 0.02043 | 0.02263 |
| 1633.927 | 0.02015 | 0.01935 | 0.02039 | 0.02331 |
| 1632.496 | 0.02017 | 0.01966 | 0.02038 | 0.02338 |
| 1631.065 | 0.02019 | 0.01958 | 0.02054 | 0.02325 |
| 1629.634 | 0.02038 | 0.01948 | 0.02076 | 0.02334 |
| 1628.204 | 0.02079 | 0.01966 | 0.02094 | 0.02376 |
| 1626.773 | 0.02126 | 0.02006 | 0.02104 | 0.02419 |
| 1625.342 | 0.0216 | 0.02043 | 0.02108 | 0.02434 |
| 1623.911 | 0.0218 | 0.02059 | 0.02117 | 0.02433 |
| 1622.481 | 0.0219 | 0.0205 | 0.02127 | 0.02434 |
| 1621.05 | 0.02187 | 0.02025 | 0.02119 | 0.02429 |
| 1619.619 | 0.0217 | 0.01994 | 0.02091 | 0.02409 |
| 1618.188 | 0.02143 | 0.01972 | 0.02059 | 0.02375 |
| 1616.758 | 0.02113 | 0.01967 | 0.0205 | 0.02333 |
| 1615.327 | 0.02088 | 0.01977 | 0.02056 | 0.02287 |
| 1613.896 | 0.02066 | 0.01978 | 0.02047 | 0.02238 |
| 1612.465 | 0.02046 | 0.01962 | 0.02017 | 0.02189 |
| 1611.035 | 0.02035 | 0.01942 | 0.01996 | 0.02154 |
| 1609.604 | 0.02045 | 0.0194 | 0.02004 | 0.0215 |
| 1608.173 | 0.02068 | 0.01961 | 0.02029 | 0.02171 |
| 1606.742 | 0.02093 | 0.01992 | 0.02046 | 0.02199 |
| 1605.312 | 0.02119 | 0.02029 | 0.02061 | 0.02226 |
| 1603.881 | 0.02157 | 0.02076 | 0.02095 | 0.02259 |
| 1602.45 | 0.02208 | 0.02133 | 0.02149 | 0.02298 |
| 1601.019 | 0.02267 | 0.02194 | 0.02209 | 0.02336 |
| 1599.589 | 0.0233 | 0.02259 | 0.02267 | 0.02373 |
| 1598.158 | 0.02399 | 0.02332 | 0.02329 | 0.02416 |
| 1596.727 | 0.02476 | 0.02415 | 0.02405 | 0.02481 |
| 1595.296 | 0.02562 | 0.02509 | 0.02496 | 0.02569 |
| 1593.865 | 0.02654 | 0.02601 | 0.02593 | 0.02665 |
| 1592.435 | 0.02751 | 0.02684 | 0.02685 | 0.02751 |
| 1591.004 | 0.02852 | 0.02761 | 0.02771 | 0.02826 |
| 1589.573 | 0.02949 | 0.02842 | 0.02856 | 0.02893 |
| 1588.142 | 0.03036 | 0.02927 | 0.02938 | 0.02958 |
| 1586.712 | 0.03109 | 0.03006 | 0.03013 | 0.03024 |
| 1585.281 | 0.03165 | 0.03067 | 0.03073 | 0.0309 |
| 1583.85 | 0.03202 | 0.03106 | 0.03111 | 0.03143 |
| 1582.419 | 0.03219 | 0.03129 | 0.03132 | 0.03175 |
| 1580.989 | 0.03223 | 0.03143 | 0.03144 | 0.03185 |
| 1579.558 | 0.03221 | 0.03156 | 0.03154 | 0.03182 |
| 1578.127 | 0.03218 | 0.03164 | 0.03159 | 0.03168 |
| 1576.696 | 0.03204 | 0.03154 | 0.03139 | 0.03135 |
| 1575.266 | 0.0317 | 0.03118 | 0.03089 | 0.03079 |
| 1573.835 | 0.03132 | 0.03076 | 0.03036 | 0.03024 |
| 1572.404 | 0.03099 | 0.03044 | 0.03 | 0.02978 |
| 1570.973 | 0.03065 | 0.03013 | 0.0297 | 0.0293 |
| 1569.543 | 0.03017 | 0.02963 | 0.02925 | 0.02866 |
| 1568.112 | 0.02958 | 0.02893 | 0.02858 | 0.02797 |
| 1566.681 | 0.02901 | 0.02823 | 0.02784 | 0.02732 |
| 1565.25 | 0.02855 | 0.02764 | 0.0272 | 0.02675 |
| 1563.82 | 0.02822 | 0.02722 | 0.02675 | 0.02626 |
| 1562.389 | 0.02801 | 0.02701 | 0.0265 | 0.02593 |
| 1560.958 | 0.02788 | 0.02702 | 0.02638 | 0.02575 |
| 1559.527 | 0.02765 | 0.02701 | 0.02611 | 0.02544 |
| 1558.097 | 0.02717 | 0.02646 | 0.02545 | 0.02466 |
| 1556.666 | 0.02667 | 0.02559 | 0.02472 | 0.02382 |
| 1555.235 | 0.02627 | 0.02486 | 0.02413 | 0.02322 |
| 1553.804 | 0.02593 | 0.02429 | 0.02354 | 0.02271 |
| 1552.373 | 0.02563 | 0.02381 | 0.02296 | 0.02218 |
| 1550.943 | 0.02528 | 0.02336 | 0.02242 | 0.02156 |
| 1549.512 | 0.02482 | 0.02284 | 0.02188 | 0.02085 |
| 1548.081 | 0.02433 | 0.02232 | 0.0214 | 0.02018 |
| 1546.65 | 0.02397 | 0.02194 | 0.02109 | 0.01972 |
| 1545.22 | 0.02373 | 0.02174 | 0.02089 | 0.01941 |
| 1543.789 | 0.02342 | 0.02162 | 0.02068 | 0.01911 |
| 1542.358 | 0.02292 | 0.0215 | 0.02036 | 0.01873 |
| 1540.927 | 0.02215 | 0.02114 | 0.01972 | 0.01808 |
| 1539.497 | 0.02115 | 0.02015 | 0.01857 | 0.01701 |
| 1538.066 | 0.02026 | 0.01894 | 0.01742 | 0.01591 |
| 1536.635 | 0.01966 | 0.01804 | 0.01666 | 0.01513 |
| 1535.204 | 0.01919 | 0.01742 | 0.01617 | 0.01454 |
| 1533.774 | 0.01866 | 0.01681 | 0.01563 | 0.01388 |
| 1532.343 | 0.01798 | 0.01608 | 0.01488 | 0.0131 |
| 1530.912 | 0.01729 | 0.01535 | 0.01411 | 0.01237 |
| 1529.481 | 0.01674 | 0.01478 | 0.01354 | 0.01182 |
| 1528.051 | 0.01629 | 0.01434 | 0.01315 | 0.0114 |
| 1526.62 | 0.0158 | 0.0139 | 0.01279 | 0.01099 |
| 1525.189 | 0.01522 | 0.0134 | 0.01235 | 0.01054 |
| 1523.758 | 0.01462 | 0.0129 | 0.01184 | 0.01005 |
| 1522.328 | 0.01401 | 0.01238 | 0.01121 | 0.00947 |
| 1520.897 | 0.01334 | 0.01178 | 0.01049 | 0.00879 |
| 1519.466 | 0.0127 | 0.01122 | 0.00987 | 0.00812 |
| 1518.035 | 0.01211 | 0.01071 | 0.00938 | 0.0075 |
| 1516.604 | 0.0115 | 0.01012 | 0.00881 | 0.00685 |
| 1515.174 | 0.01084 | 0.0094 | 0.00808 | 0.00615 |
| 1513.743 | 0.01022 | 0.00872 | 0.00739 | 0.00554 |
| 1512.312 | 0.00979 | 0.00828 | 0.00692 | 0.00512 |
| 1510.881 | 0.00958 | 0.00814 | 0.00676 | 0.00496 |
| 1509.451 | 0.00952 | 0.00824 | 0.00683 | 0.00496 |
| 1508.02 | 0.00922 | 0.00819 | 0.00672 | 0.00475 |
| 1506.589 | 0.00812 | 0.00733 | 0.00583 | 0.0038 |
| 1505.158 | 0.00673 | 0.00599 | 0.00451 | 0.00255 |
| 1503.728 | 0.00585 | 0.00499 | 0.00366 | 0.00177 |
| 1502.297 | 0.0054 | 0.00441 | 0.00325 | 0.00139 |
| 1500.866 | 0.00513 | 0.00406 | 0.00302 | 0.00118 |
| 1499.435 | 0.0049 | 0.00383 | 0.00284 | 0.00099 |
| 1498.005 | 0.00463 | 0.00367 | 0.00262 | 0.00076 |
| 1496.574 | 0.00428 | 0.00344 | 0.00231 | 0.00041 |
| 1495.143 | 0.00389 | 0.00312 | 0.00192 | 0 |
| 1493.712 | 0.00362 | 0.00286 | 0.00161 | -0.0003 |
| 1492.282 | 0.00358 | 0.00281 | 0.00151 | -0.00037 |
| 1490.851 | 0.00368 | 0.00295 | 0.00159 | -0.00026 |
| 1489.42 | 0.00366 | 0.00303 | 0.00162 | -0.00021 |
| 1487.989 | 0.00334 | 0.00278 | 0.00138 | -0.00044 |
| 1486.559 | 0.00286 | 0.00234 | 0.00097 | -0.00086 |
| 1485.128 | 0.00246 | 0.00192 | 0.00058 | -0.00123 |
| 1483.697 | 0.0022 | 0.00162 | 0.0003 | -0.00147 |
| 1482.266 | 0.00203 | 0.00143 | 0.00014 | -0.00162 |
| 1480.836 | 0.00192 | 0.00133 | 0.00006 | -0.00174 |
| 1479.405 | 0.00189 | 0.00131 | 0.00005 | -0.00182 |
| 1477.974 | 0.00197 | 0.00143 | 0.00013 | -0.00179 |
| 1476.543 | 0.00216 | 0.00166 | 0.0003 | -0.00162 |
| 1475.112 | 0.00238 | 0.00195 | 0.00054 | -0.0014 |
| 1473.682 | 0.00246 | 0.00212 | 0.00069 | -0.00131 |
| 1472.251 | 0.00225 | 0.00195 | 0.00054 | -0.00149 |
| 1470.82 | 0.00185 | 0.00154 | 0.00014 | -0.00184 |
| 1469.389 | 0.00156 | 0.00119 | -0.00018 | -0.0021 |
| 1467.959 | 0.00151 | 0.00109 | -0.00023 | -0.00215 |
| 1466.528 | 0.00169 | 0.00126 | -0.00002 | -0.00194 |
| 1465.097 | 0.00206 | 0.00163 | 0.00031 | -0.00153 |
| 1463.666 | 0.0025 | 0.00203 | 0.00064 | -0.00104 |
| 1462.236 | 0.00298 | 0.00243 | 0.00101 | -0.00056 |
| 1460.805 | 0.00357 | 0.00297 | 0.00157 | -0.00001 |
| 1459.374 | 0.00435 | 0.00377 | 0.00241 | 0.00073 |
| 1457.943 | 0.00533 | 0.00477 | 0.00343 | 0.00163 |
| 1456.513 | 0.00601 | 0.00539 | 0.00408 | 0.00224 |
| 1455.082 | 0.00604 | 0.00532 | 0.00405 | 0.00226 |
| 1453.651 | 0.00575 | 0.00499 | 0.00376 | 0.00199 |
| 1452.22 | 0.0054 | 0.00471 | 0.00352 | 0.00173 |
| 1450.79 | 0.00509 | 0.00451 | 0.00337 | 0.00157 |
| 1449.359 | 0.00479 | 0.00434 | 0.00325 | 0.00148 |
| 1447.928 | 0.00444 | 0.0041 | 0.00303 | 0.00136 |
| 1446.497 | 0.00403 | 0.00374 | 0.00269 | 0.00112 |
| 1445.067 | 0.00363 | 0.00337 | 0.00236 | 0.00084 |
| 1443.636 | 0.00335 | 0.00308 | 0.00215 | 0.00062 |
| 1442.205 | 0.00318 | 0.00292 | 0.00208 | 0.00049 |
| 1440.774 | 0.0031 | 0.00285 | 0.00209 | 0.00044 |
| 1439.344 | 0.0031 | 0.0029 | 0.00214 | 0.00046 |
| 1437.913 | 0.00317 | 0.00304 | 0.00224 | 0.00056 |
| 1436.482 | 0.00325 | 0.00315 | 0.00234 | 0.00068 |
| 1435.051 | 0.00322 | 0.00311 | 0.00235 | 0.0007 |
| 1433.62 | 0.00312 | 0.00301 | 0.00229 | 0.00068 |
| 1432.19 | 0.00307 | 0.00304 | 0.00232 | 0.00075 |
| 1430.759 | 0.00314 | 0.00327 | 0.0025 | 0.00098 |
| 1429.328 | 0.00332 | 0.00358 | 0.00283 | 0.00133 |
| 1427.897 | 0.00358 | 0.00389 | 0.00324 | 0.00173 |
| 1426.467 | 0.00389 | 0.00421 | 0.00369 | 0.0022 |
| 1425.036 | 0.00426 | 0.00461 | 0.00418 | 0.00276 |
| 1423.605 | 0.00466 | 0.0051 | 0.00469 | 0.00338 |
| 1422.174 | 0.00511 | 0.00565 | 0.00527 | 0.00401 |
| 1420.744 | 0.00572 | 0.00635 | 0.00606 | 0.00476 |
| 1419.313 | 0.00661 | 0.00726 | 0.00706 | 0.00571 |
| 1417.882 | 0.00754 | 0.00816 | 0.00794 | 0.0066 |
| 1416.451 | 0.00826 | 0.00879 | 0.00847 | 0.00718 |
| 1415.021 | 0.0089 | 0.00932 | 0.00891 | 0.00765 |
| 1413.59 | 0.00971 | 0.00998 | 0.00952 | 0.00825 |
| 1412.159 | 0.01074 | 0.01083 | 0.01033 | 0.00904 |
| 1410.728 | 0.01187 | 0.01175 | 0.01118 | 0.00993 |
| 1409.298 | 0.01296 | 0.0126 | 0.01192 | 0.01076 |
| 1407.867 | 0.01392 | 0.01335 | 0.01256 | 0.01147 |
| 1406.436 | 0.01479 | 0.01399 | 0.01317 | 0.01205 |
| 1405.005 | 0.0155 | 0.01446 | 0.01367 | 0.01242 |
| 1403.575 | 0.01594 | 0.01467 | 0.01389 | 0.01249 |
| 1402.144 | 0.01613 | 0.01469 | 0.01385 | 0.01235 |
| 1400.713 | 0.01614 | 0.01461 | 0.01367 | 0.01211 |
| 1399.282 | 0.01598 | 0.01438 | 0.01338 | 0.01177 |
| 1397.852 | 0.01557 | 0.01389 | 0.01291 | 0.01124 |
| 1396.421 | 0.01486 | 0.01312 | 0.01218 | 0.01045 |
| 1394.99 | 0.01387 | 0.01212 | 0.01117 | 0.00941 |
| 1393.559 | 0.01273 | 0.01104 | 0.01002 | 0.00826 |
| 1392.128 | 0.01169 | 0.0101 | 0.00903 | 0.00725 |
| 1390.698 | 0.01088 | 0.00937 | 0.00832 | 0.00649 |
| 1389.267 | 0.01021 | 0.00878 | 0.00778 | 0.0059 |
| 1387.836 | 0.00955 | 0.00817 | 0.00721 | 0.00533 |
| 1386.405 | 0.00881 | 0.00747 | 0.00652 | 0.00469 |
| 1384.975 | 0.00808 | 0.00676 | 0.00582 | 0.00403 |
| 1383.544 | 0.00748 | 0.00616 | 0.00525 | 0.00347 |
| 1382.113 | 0.00706 | 0.00573 | 0.00483 | 0.00306 |
| 1380.682 | 0.00675 | 0.00542 | 0.00449 | 0.00275 |
| 1379.252 | 0.00646 | 0.00515 | 0.00417 | 0.00246 |
| 1377.821 | 0.00614 | 0.0049 | 0.00388 | 0.00219 |
| 1376.39 | 0.00583 | 0.00469 | 0.00366 | 0.00192 |
| 1374.959 | 0.00555 | 0.00451 | 0.00349 | 0.00167 |
| 1373.529 | 0.00524 | 0.00426 | 0.00324 | 0.00137 |
| 1372.098 | 0.00492 | 0.00392 | 0.00288 | 0.00107 |
| 1370.667 | 0.00466 | 0.00361 | 0.00256 | 0.00084 |
| 1369.236 | 0.00446 | 0.00338 | 0.00235 | 0.00071 |
| 1367.806 | 0.00426 | 0.00321 | 0.00221 | 0.00062 |
| 1366.375 | 0.00411 | 0.0031 | 0.00213 | 0.00057 |
| 1364.944 | 0.00407 | 0.00311 | 0.00214 | 0.0006 |
| 1363.513 | 0.0041 | 0.00318 | 0.0022 | 0.00065 |
| 1362.083 | 0.00404 | 0.00316 | 0.00215 | 0.00055 |
| 1360.652 | 0.00382 | 0.00295 | 0.00197 | 0.00028 |
| 1359.221 | 0.00355 | 0.00269 | 0.00175 | -0.00002 |
| 1357.79 | 0.00336 | 0.00252 | 0.00161 | -0.0002 |
| 1356.359 | 0.00328 | 0.00248 | 0.00155 | -0.00026 |
| 1354.929 | 0.00326 | 0.0025 | 0.00153 | -0.00024 |
| 1353.498 | 0.00328 | 0.00254 | 0.00155 | -0.00019 |
| 1352.067 | 0.0033 | 0.00256 | 0.00158 | -0.00012 |
| 1350.636 | 0.00334 | 0.00259 | 0.00163 | -0.00005 |
| 1349.206 | 0.0034 | 0.00263 | 0.00165 | 0.00002 |
| 1347.775 | 0.00349 | 0.00268 | 0.00165 | 0.00008 |
| 1346.344 | 0.00358 | 0.00275 | 0.00166 | 0.00014 |
| 1344.913 | 0.00367 | 0.00282 | 0.00169 | 0.00021 |
| 1343.483 | 0.0038 | 0.0029 | 0.00179 | 0.00032 |
| 1342.052 | 0.00403 | 0.00304 | 0.00202 | 0.00051 |
| 1340.621 | 0.00434 | 0.00325 | 0.00233 | 0.00077 |
| 1339.19 | 0.00455 | 0.00339 | 0.00257 | 0.00094 |
| 1337.76 | 0.00451 | 0.00335 | 0.00256 | 0.00086 |
| 1336.329 | 0.00429 | 0.00319 | 0.00238 | 0.00062 |
| 1334.898 | 0.00411 | 0.00306 | 0.00221 | 0.00042 |
| 1333.467 | 0.00406 | 0.00305 | 0.00217 | 0.00036 |
| 1332.037 | 0.00413 | 0.00312 | 0.00225 | 0.00043 |
| 1330.606 | 0.00425 | 0.0032 | 0.00235 | 0.00057 |
| 1329.175 | 0.00436 | 0.00327 | 0.00244 | 0.00073 |
| 1327.744 | 0.00449 | 0.00336 | 0.00253 | 0.00093 |
| 1326.314 | 0.00464 | 0.00349 | 0.00264 | 0.00113 |
| 1324.883 | 0.0048 | 0.00361 | 0.00276 | 0.00129 |
| 1323.452 | 0.00493 | 0.00372 | 0.00287 | 0.00138 |
| 1322.021 | 0.00503 | 0.00384 | 0.003 | 0.00146 |
| 1320.591 | 0.00507 | 0.00398 | 0.00314 | 0.00156 |
| 1319.16 | 0.00508 | 0.00411 | 0.00323 | 0.00168 |
| 1317.729 | 0.00506 | 0.00415 | 0.00324 | 0.00173 |
| 1316.298 | 0.00503 | 0.00413 | 0.00321 | 0.0017 |
| 1314.867 | 0.005 | 0.00407 | 0.00318 | 0.00163 |
| 1313.437 | 0.00495 | 0.00402 | 0.00315 | 0.00156 |
| 1312.006 | 0.00483 | 0.00393 | 0.00303 | 0.00145 |
| 1310.575 | 0.00464 | 0.00377 | 0.00281 | 0.00129 |
| 1309.144 | 0.00444 | 0.00358 | 0.00257 | 0.00111 |
| 1307.714 | 0.00427 | 0.0034 | 0.00237 | 0.00096 |
| 1306.283 | 0.00411 | 0.00326 | 0.00221 | 0.00083 |
| 1304.852 | 0.00396 | 0.00312 | 0.00209 | 0.00074 |
| 1303.421 | 0.00384 | 0.00298 | 0.002 | 0.00068 |
| 1301.991 | 0.00378 | 0.00288 | 0.00198 | 0.00064 |
| 1300.56 | 0.00374 | 0.00282 | 0.00199 | 0.00058 |
| 1299.129 | 0.00371 | 0.00275 | 0.002 | 0.00047 |
| 1297.698 | 0.00366 | 0.00266 | 0.00196 | 0.00032 |
| 1296.268 | 0.0036 | 0.00258 | 0.00188 | 0.00019 |
| 1294.837 | 0.00356 | 0.00256 | 0.00182 | 0.00012 |
| 1293.406 | 0.00352 | 0.00258 | 0.00181 | 0.00015 |
| 1291.975 | 0.00347 | 0.00261 | 0.00185 | 0.00024 |
| 1290.545 | 0.0034 | 0.00265 | 0.0019 | 0.00034 |
| 1289.114 | 0.00333 | 0.00266 | 0.0019 | 0.00044 |
| 1287.683 | 0.00328 | 0.00264 | 0.00186 | 0.00049 |
| 1286.252 | 0.00322 | 0.00258 | 0.0018 | 0.00048 |
| 1284.822 | 0.00317 | 0.00251 | 0.00178 | 0.00043 |
| 1283.391 | 0.00315 | 0.00249 | 0.00182 | 0.0004 |
| 1281.96 | 0.00318 | 0.00253 | 0.00188 | 0.00042 |
| 1280.529 | 0.00327 | 0.0026 | 0.00193 | 0.00049 |
| 1279.099 | 0.00336 | 0.00268 | 0.00194 | 0.00055 |
| 1277.668 | 0.00341 | 0.00273 | 0.00197 | 0.00057 |
| 1276.237 | 0.00342 | 0.00276 | 0.00204 | 0.00057 |
| 1274.806 | 0.00344 | 0.00279 | 0.00213 | 0.00061 |
| 1273.375 | 0.0035 | 0.00279 | 0.00219 | 0.0007 |
| 1271.945 | 0.00357 | 0.00278 | 0.00218 | 0.00077 |
| 1270.514 | 0.00359 | 0.00273 | 0.0021 | 0.00076 |
| 1269.083 | 0.00352 | 0.00266 | 0.00202 | 0.00068 |
| 1267.652 | 0.0034 | 0.00257 | 0.00192 | 0.00056 |
| 1266.222 | 0.00324 | 0.00246 | 0.00179 | 0.00041 |
| 1264.791 | 0.00304 | 0.00233 | 0.0016 | 0.00025 |
| 1263.36 | 0.00284 | 0.00219 | 0.00139 | 0.00008 |
| 1261.929 | 0.00269 | 0.00206 | 0.00121 | -0.00006 |
| 1260.499 | 0.00257 | 0.00192 | 0.00107 | -0.00018 |
| 1259.068 | 0.00246 | 0.00176 | 0.00092 | -0.00032 |
| 1257.637 | 0.00234 | 0.0016 | 0.00074 | -0.00049 |
| 1256.206 | 0.00223 | 0.00148 | 0.00059 | -0.00068 |
| 1254.776 | 0.00213 | 0.00137 | 0.0005 | -0.00085 |
| 1253.345 | 0.00205 | 0.00125 | 0.00046 | -0.00097 |
| 1251.914 | 0.00198 | 0.00114 | 0.00043 | -0.00102 |
| 1250.483 | 0.00191 | 0.00106 | 0.00038 | -0.00102 |
| 1249.053 | 0.00188 | 0.00101 | 0.00035 | -0.00099 |
| 1247.622 | 0.00186 | 0.00101 | 0.00035 | -0.00095 |
| 1246.191 | 0.00184 | 0.001 | 0.00038 | -0.00095 |
| 1244.76 | 0.00182 | 0.00098 | 0.00039 | -0.001 |
| 1243.33 | 0.00179 | 0.00096 | 0.00036 | -0.00108 |
| 1241.899 | 0.00177 | 0.00095 | 0.00032 | -0.00114 |
| 1240.468 | 0.00177 | 0.00097 | 0.00028 | -0.00117 |
| 1239.037 | 0.00177 | 0.00098 | 0.00025 | -0.00119 |
| 1237.606 | 0.00177 | 0.00093 | 0.00019 | -0.0012 |
| 1236.176 | 0.00179 | 0.00086 | 0.00015 | -0.00121 |
| 1234.745 | 0.00184 | 0.00083 | 0.00017 | -0.0012 |
| 1233.314 | 0.00189 | 0.00083 | 0.00023 | -0.00121 |
| 1231.883 | 0.00188 | 0.00082 | 0.00027 | -0.00125 |
| 1230.453 | 0.0018 | 0.00075 | 0.00025 | -0.0013 |
| 1229.022 | 0.00168 | 0.00065 | 0.00016 | -0.00133 |
| 1227.591 | 0.00156 | 0.00058 | 0.00004 | -0.00135 |
| 1226.16 | 0.00146 | 0.00053 | -0.00007 | -0.00138 |
| 1224.73 | 0.00138 | 0.00049 | -0.00015 | -0.00143 |
| 1223.299 | 0.00128 | 0.00044 | -0.00019 | -0.0015 |
| 1221.868 | 0.00116 | 0.0004 | -0.00021 | -0.00158 |
| 1220.437 | 0.00104 | 0.00036 | -0.00024 | -0.00167 |
| 1219.007 | 0.00096 | 0.00031 | -0.00028 | -0.0018 |
| 1217.576 | 0.0009 | 0.00023 | -0.00033 | -0.00193 |
| 1216.145 | 0.00088 | 0.00017 | -0.00035 | -0.002 |
| 1214.714 | 0.00088 | 0.00016 | -0.00035 | -0.00197 |
| 1213.284 | 0.00085 | 0.00017 | -0.00037 | -0.00189 |
| 1211.853 | 0.00078 | 0.00016 | -0.00041 | -0.00182 |
| 1210.422 | 0.0007 | 0.00014 | -0.00044 | -0.00178 |
| 1208.991 | 0.00066 | 0.00013 | -0.00043 | -0.00175 |
| 1207.561 | 0.00066 | 0.00012 | -0.00041 | -0.00171 |
| 1206.13 | 0.00068 | 0.0001 | -0.00041 | -0.0017 |
| 1204.699 | 0.00067 | 0.00008 | -0.00046 | -0.00173 |
| 1203.268 | 0.00062 | 0.00006 | -0.00053 | -0.0018 |
| 1201.838 | 0.00057 | 0.00005 | -0.00058 | -0.00186 |
| 1200.407 | 0.00055 | 0.00002 | -0.00058 | -0.00191 |
| 1198.976 | 0.00056 | -0.00003 | -0.00056 | -0.00195 |
| 1197.545 | 0.00057 | -0.00009 | -0.00054 | -0.00199 |
| 1196.114 | 0.00057 | -0.00012 | -0.00056 | -0.00198 |
| 1194.684 | 0.00058 | -0.00011 | -0.00056 | -0.00194 |
| 1193.253 | 0.00062 | -0.00009 | -0.00054 | -0.00188 |
| 1191.822 | 0.00069 | -0.00006 | -0.00049 | -0.00183 |
| 1190.391 | 0.00074 | 0 | -0.00046 | -0.00177 |
| 1188.961 | 0.00077 | 0.00006 | -0.00045 | -0.00172 |
| 1187.53 | 0.00079 | 0.00009 | -0.00046 | -0.0017 |
| 1186.099 | 0.00081 | 0.00008 | -0.00045 | -0.00172 |
| 1184.668 | 0.0008 | 0.00003 | -0.00045 | -0.00176 |
| 1183.238 | 0.00077 | -0.00004 | -0.00049 | -0.00183 |
| 1181.807 | 0.00072 | -0.00011 | -0.00057 | -0.0019 |
| 1180.376 | 0.00067 | -0.00016 | -0.00065 | -0.00195 |
| 1178.945 | 0.00062 | -0.00016 | -0.0007 | -0.002 |
| 1177.515 | 0.00056 | -0.00015 | -0.0007 | -0.00206 |
| 1176.084 | 0.00053 | -0.00014 | -0.00072 | -0.00213 |
| 1174.653 | 0.00048 | -0.00019 | -0.00078 | -0.0022 |
| 1173.222 | 0.00038 | -0.00028 | -0.00086 | -0.00226 |
| 1171.792 | 0.00026 | -0.00038 | -0.00093 | -0.00229 |
| 1170.361 | 0.00015 | -0.00044 | -0.00097 | -0.00228 |
| 1168.93 | 0.00008 | -0.00049 | -0.00101 | -0.00226 |
| 1167.499 | 0.00002 | -0.00052 | -0.0011 | -0.00227 |
| 1166.069 | -0.00001 | -0.00054 | -0.00121 | -0.00232 |
| 1164.638 | -0.00002 | -0.00057 | -0.00131 | -0.00241 |
| 1163.207 | -0.00006 | -0.00063 | -0.00136 | -0.00255 |
| 1161.776 | -0.00016 | -0.00069 | -0.00139 | -0.00272 |
| 1160.346 | -0.0003 | -0.00073 | -0.00145 | -0.00289 |
| 1158.915 | -0.00043 | -0.00078 | -0.00157 | -0.00299 |
| 1157.484 | -0.00053 | -0.00085 | -0.00167 | -0.00301 |
| 1156.053 | -0.00057 | -0.00093 | -0.00165 | -0.00298 |
| 1154.622 | -0.00058 | -0.00098 | -0.00155 | -0.00295 |
| 1153.192 | -0.00058 | -0.00101 | -0.00148 | -0.00295 |
| 1151.761 | -0.00056 | -0.00107 | -0.00153 | -0.00296 |
| 1150.33 | -0.00049 | -0.00113 | -0.00162 | -0.00294 |
| 1148.899 | -0.0004 | -0.00113 | -0.00164 | -0.00289 |
| 1147.469 | -0.00031 | -0.00104 | -0.00154 | -0.00281 |
| 1146.038 | -0.00023 | -0.0009 | -0.00141 | -0.00272 |
| 1144.607 | -0.00012 | -0.00078 | -0.00131 | -0.00265 |
| 1143.176 | 0.00001 | -0.00068 | -0.00123 | -0.00262 |
| 1141.746 | 0.00014 | -0.00059 | -0.00112 | -0.00257 |
| 1140.315 | 0.00026 | -0.00048 | -0.00094 | -0.00244 |
| 1138.884 | 0.00039 | -0.00036 | -0.00075 | -0.00225 |
| 1137.453 | 0.00052 | -0.00024 | -0.00062 | -0.00204 |
| 1136.023 | 0.00065 | -0.00013 | -0.00053 | -0.00184 |
| 1134.592 | 0.00077 | -0.00001 | -0.0004 | -0.00166 |
| 1133.161 | 0.00085 | 0.00008 | -0.00022 | -0.00151 |
| 1131.73 | 0.00085 | 0.00011 | -0.0001 | -0.00143 |
| 1130.3 | 0.00081 | 0.00006 | -0.00012 | -0.00145 |
| 1128.869 | 0.00077 | -0.00004 | -0.00026 | -0.00151 |
| 1127.438 | 0.00074 | -0.00012 | -0.00039 | -0.00152 |
| 1126.007 | 0.00071 | -0.00016 | -0.00042 | -0.00149 |
| 1124.577 | 0.00064 | -0.00019 | -0.0004 | -0.00153 |
| 1123.146 | 0.00051 | -0.00022 | -0.00044 | -0.00167 |
| 1121.715 | 0.00036 | -0.00027 | -0.0006 | -0.00188 |
| 1120.284 | 0.00021 | -0.00036 | -0.0008 | -0.00206 |
| 1118.854 | 0.00012 | -0.00046 | -0.00092 | -0.00217 |
| 1117.423 | 0.00003 | -0.00058 | -0.00094 | -0.00225 |
| 1115.992 | -0.00006 | -0.00069 | -0.00095 | -0.00231 |
| 1114.561 | -0.00014 | -0.00078 | -0.00101 | -0.00233 |
| 1113.13 | -0.00018 | -0.00084 | -0.00108 | -0.00234 |
| 1111.7 | -0.00022 | -0.00086 | -0.00113 | -0.00237 |
| 1110.269 | -0.0003 | -0.00091 | -0.00119 | -0.00244 |
| 1108.838 | -0.00041 | -0.00103 | -0.0013 | -0.00254 |
| 1107.407 | -0.00048 | -0.00113 | -0.00147 | -0.00262 |
| 1105.977 | -0.0005 | -0.00114 | -0.00163 | -0.00265 |
| 1104.546 | -0.00052 | -0.00108 | -0.0017 | -0.0027 |
| 1103.115 | -0.0006 | -0.00107 | -0.00168 | -0.00281 |
| 1101.684 | -0.0007 | -0.00112 | -0.00165 | -0.00292 |
| 1100.254 | -0.00078 | -0.00117 | -0.0017 | -0.00299 |
| 1098.823 | -0.00082 | -0.00118 | -0.00182 | -0.00302 |
| 1097.392 | -0.00084 | -0.00117 | -0.00191 | -0.00305 |
| 1095.961 | -0.00082 | -0.00115 | -0.00188 | -0.00303 |
| 1094.531 | -0.00071 | -0.00106 | -0.00175 | -0.00288 |
| 1093.1 | -0.00057 | -0.00093 | -0.00161 | -0.00264 |
| 1091.669 | -0.00046 | -0.00085 | -0.00154 | -0.00244 |
| 1090.238 | -0.00044 | -0.00089 | -0.00154 | -0.00238 |
| 1088.808 | -0.00047 | -0.00102 | -0.00153 | -0.00244 |
| 1087.377 | -0.00046 | -0.00112 | -0.00146 | -0.00252 |
| 1085.946 | -0.00041 | -0.00115 | -0.00138 | -0.00261 |
| 1084.515 | -0.00034 | -0.00115 | -0.00136 | -0.0027 |
| 1083.085 | -0.0003 | -0.00115 | -0.0014 | -0.00278 |
| 1081.654 | -0.00029 | -0.00115 | -0.00143 | -0.00284 |
| 1080.223 | -0.00032 | -0.00115 | -0.00141 | -0.00287 |
| 1078.792 | -0.00041 | -0.00114 | -0.00137 | -0.00287 |
| 1077.361 | -0.00055 | -0.00114 | -0.00137 | -0.00285 |
| 1075.931 | -0.00067 | -0.00117 | -0.00141 | -0.0028 |
| 1074.5 | -0.00069 | -0.00122 | -0.00143 | -0.00272 |
| 1073.069 | -0.00062 | -0.00125 | -0.00137 | -0.00259 |
| 1071.638 | -0.00054 | -0.00123 | -0.00127 | -0.0025 |
| 1070.208 | -0.00052 | -0.00123 | -0.00125 | -0.0025 |
| 1068.777 | -0.00054 | -0.00127 | -0.00136 | -0.00258 |
| 1067.346 | -0.00056 | -0.00129 | -0.0015 | -0.00268 |
| 1065.915 | -0.00057 | -0.00123 | -0.00157 | -0.0028 |
| 1064.485 | -0.00057 | -0.00113 | -0.00156 | -0.00291 |
| 1063.054 | -0.00056 | -0.00108 | -0.00155 | -0.003 |
| 1061.623 | -0.00056 | -0.00114 | -0.00162 | -0.00305 |
| 1060.192 | -0.00059 | -0.00126 | -0.00171 | -0.00309 |
| 1058.762 | -0.00063 | -0.00135 | -0.00173 | -0.00311 |
| 1057.331 | -0.00066 | -0.00135 | -0.00163 | -0.00305 |
| 1055.9 | -0.00065 | -0.0013 | -0.00151 | -0.00292 |
| 1054.469 | -0.00058 | -0.00122 | -0.00148 | -0.00275 |
| 1053.039 | -0.00047 | -0.00115 | -0.00151 | -0.00264 |
| 1051.608 | -0.00038 | -0.00109 | -0.0015 | -0.00261 |
| 1050.177 | -0.00035 | -0.00105 | -0.00142 | -0.00262 |
| 1048.746 | -0.00037 | -0.00101 | -0.00134 | -0.00263 |
| 1047.316 | -0.00042 | -0.00097 | -0.00133 | -0.00263 |
| 1045.885 | -0.00046 | -0.00096 | -0.00139 | -0.00267 |
| 1044.454 | -0.00047 | -0.001 | -0.00145 | -0.00278 |
| 1043.023 | -0.00044 | -0.00104 | -0.00145 | -0.00287 |
| 1041.593 | -0.00035 | -0.00103 | -0.00139 | -0.00285 |
| 1040.162 | -0.00029 | -0.00096 | -0.00137 | -0.00269 |
| 1038.731 | -0.00031 | -0.0009 | -0.00146 | -0.00253 |
| 1037.3 | -0.00041 | -0.00088 | -0.00157 | -0.00245 |
| 1035.869 | -0.00048 | -0.00091 | -0.00158 | -0.0024 |
| 1034.439 | -0.00044 | -0.00093 | -0.00142 | -0.00235 |
| 1033.008 | -0.00031 | -0.0009 | -0.00122 | -0.00231 |
| 1031.577 | -0.00015 | -0.0008 | -0.00112 | -0.00231 |
| 1030.146 | -0.00007 | -0.00068 | -0.00115 | -0.00235 |
| 1028.716 | -0.00013 | -0.00065 | -0.00122 | -0.00244 |
| 1027.285 | -0.00029 | -0.0007 | -0.00127 | -0.00254 |
| 1025.854 | -0.00045 | -0.0008 | -0.00133 | -0.00265 |
| 1024.423 | -0.00056 | -0.00089 | -0.00144 | -0.00276 |
| 1022.993 | -0.00061 | -0.00097 | -0.00158 | -0.00284 |
| 1021.562 | -0.00062 | -0.00099 | -0.00164 | -0.00283 |
| 1020.131 | -0.00061 | -0.00097 | -0.0016 | -0.00277 |
| 1018.7 | -0.00061 | -0.00097 | -0.00151 | -0.00276 |
| 1017.27 | -0.00062 | -0.00105 | -0.00151 | -0.00282 |
| 1015.839 | -0.00066 | -0.00117 | -0.00163 | -0.0029 |
| 1014.408 | -0.00073 | -0.00125 | -0.00181 | -0.00294 |
| 1012.977 | -0.00077 | -0.0013 | -0.00193 | -0.00296 |
| 1011.547 | -0.00077 | -0.00134 | -0.00193 | -0.00298 |
| 1010.116 | -0.00074 | -0.00135 | -0.00187 | -0.00302 |
| 1008.685 | -0.00073 | -0.00133 | -0.00181 | -0.00307 |
| 1007.254 | -0.00074 | -0.00132 | -0.00175 | -0.00313 |
| 1005.824 | -0.00077 | -0.00133 | -0.00169 | -0.00319 |
| 1004.393 | -0.00078 | -0.00134 | -0.00161 | -0.00322 |
| 1002.962 | -0.00075 | -0.0013 | -0.00153 | -0.0032 |
| 1001.531 | -0.00069 | -0.00122 | -0.00146 | -0.00314 |
| 1000.101 | -0.00061 | -0.00115 | -0.00141 | -0.00311 |

(c) ATR-FTIR absorbance of γ-PGA, γ-PGA/Mg^2+^ complexation at different Mg^2+^:Glutamate ratios datasets.

| Wavenumber  (cm^-1^) | γ-PGA | Mg^2+^:Glutamate | | |
| --- | --- | --- | --- | --- |
|  |  | 1:2 | 1:1 | 2:1 |
| 2000.201 | -0.00095 | -0.00147 | -0.00141 | -0.0003 |
| 1998.77 | -0.00095 | -0.00147 | -0.0014 | -0.00027 |
| 1997.34 | -0.00093 | -0.00146 | -0.00138 | -0.00024 |
| 1995.909 | -0.00089 | -0.00145 | -0.00135 | -0.0002 |
| 1994.478 | -0.00084 | -0.00147 | -0.00132 | -0.00017 |
| 1993.047 | -0.00079 | -0.0015 | -0.00129 | -0.00017 |
| 1991.616 | -0.00078 | -0.00152 | -0.00128 | -0.0002 |
| 1990.186 | -0.00081 | -0.00151 | -0.0013 | -0.00024 |
| 1988.755 | -0.00087 | -0.00149 | -0.00134 | -0.00027 |
| 1987.324 | -0.00092 | -0.00149 | -0.00139 | -0.00029 |
| 1985.893 | -0.00096 | -0.00151 | -0.00144 | -0.0003 |
| 1984.463 | -0.00097 | -0.00152 | -0.00146 | -0.00028 |
| 1983.032 | -0.00095 | -0.00151 | -0.00145 | -0.00025 |
| 1981.601 | -0.00094 | -0.00147 | -0.00141 | -0.00021 |
| 1980.17 | -0.00093 | -0.00143 | -0.00137 | -0.00019 |
| 1978.74 | -0.00092 | -0.00141 | -0.00134 | -0.0002 |
| 1977.309 | -0.00091 | -0.00143 | -0.00133 | -0.00024 |
| 1975.878 | -0.00093 | -0.00146 | -0.00134 | -0.00028 |
| 1974.447 | -0.00096 | -0.0015 | -0.00137 | -0.0003 |
| 1973.017 | -0.00098 | -0.00152 | -0.00141 | -0.00031 |
| 1971.586 | -0.00099 | -0.00153 | -0.00142 | -0.0003 |
| 1970.155 | -0.00096 | -0.00154 | -0.00139 | -0.00027 |
| 1968.724 | -0.00089 | -0.00153 | -0.00132 | -0.00024 |
| 1967.294 | -0.00084 | -0.00151 | -0.00126 | -0.00022 |
| 1965.863 | -0.00083 | -0.00149 | -0.00125 | -0.00021 |
| 1964.432 | -0.00085 | -0.00146 | -0.00128 | -0.0002 |
| 1963.001 | -0.00088 | -0.00144 | -0.00131 | -0.00018 |
| 1961.571 | -0.0009 | -0.00144 | -0.00133 | -0.00019 |
| 1960.14 | -0.00094 | -0.00145 | -0.00136 | -0.00022 |
| 1958.709 | -0.00097 | -0.00148 | -0.0014 | -0.00026 |
| 1957.278 | -0.00097 | -0.00151 | -0.00141 | -0.00028 |
| 1955.847 | -0.00095 | -0.00151 | -0.0014 | -0.00028 |
| 1954.417 | -0.00095 | -0.0015 | -0.00142 | -0.00029 |
| 1952.986 | -0.00097 | -0.0015 | -0.00144 | -0.00031 |
| 1951.555 | -0.00099 | -0.00149 | -0.00145 | -0.00031 |
| 1950.124 | -0.00097 | -0.00149 | -0.00143 | -0.00028 |
| 1948.694 | -0.00093 | -0.00148 | -0.00139 | -0.00024 |
| 1947.263 | -0.00087 | -0.00148 | -0.00134 | -0.0002 |
| 1945.832 | -0.00079 | -0.00148 | -0.00126 | -0.00017 |
| 1944.401 | -0.0007 | -0.00148 | -0.00118 | -0.00015 |
| 1942.971 | -0.00064 | -0.00148 | -0.00114 | -0.00014 |
| 1941.54 | -0.00068 | -0.00148 | -0.00118 | -0.00018 |
| 1940.109 | -0.0008 | -0.00148 | -0.00128 | -0.00023 |
| 1938.678 | -0.00091 | -0.00147 | -0.00137 | -0.00025 |
| 1937.248 | -0.00097 | -0.00147 | -0.00142 | -0.00025 |
| 1935.817 | -0.00098 | -0.00148 | -0.00144 | -0.00023 |
| 1934.386 | -0.00097 | -0.0015 | -0.00144 | -0.00022 |
| 1932.955 | -0.00098 | -0.00152 | -0.00143 | -0.00022 |
| 1931.525 | -0.00098 | -0.00151 | -0.00143 | -0.00022 |
| 1930.094 | -0.00099 | -0.0015 | -0.00142 | -0.00022 |
| 1928.663 | -0.00102 | -0.0015 | -0.00143 | -0.00024 |
| 1927.232 | -0.00103 | -0.0015 | -0.00143 | -0.00025 |
| 1925.802 | -0.00097 | -0.00152 | -0.0014 | -0.00024 |
| 1924.371 | -0.00085 | -0.00152 | -0.00133 | -0.0002 |
| 1922.94 | -0.00076 | -0.00151 | -0.00127 | -0.00015 |
| 1921.509 | -0.00074 | -0.00151 | -0.00126 | -0.00014 |
| 1920.079 | -0.00077 | -0.00153 | -0.00127 | -0.00017 |
| 1918.648 | -0.00079 | -0.00154 | -0.00127 | -0.00019 |
| 1917.217 | -0.00081 | -0.00153 | -0.00127 | -0.00022 |
| 1915.786 | -0.00086 | -0.0015 | -0.00132 | -0.00024 |
| 1914.355 | -0.00093 | -0.00149 | -0.00138 | -0.00026 |
| 1912.925 | -0.00096 | -0.00152 | -0.00142 | -0.00027 |
| 1911.494 | -0.00094 | -0.00154 | -0.00139 | -0.00026 |
| 1910.063 | -0.0009 | -0.00155 | -0.00135 | -0.00025 |
| 1908.632 | -0.00091 | -0.00155 | -0.00135 | -0.00026 |
| 1907.202 | -0.00097 | -0.00156 | -0.00141 | -0.00029 |
| 1905.771 | -0.00104 | -0.00157 | -0.00147 | -0.00031 |
| 1904.34 | -0.00107 | -0.00155 | -0.00149 | -0.00031 |
| 1902.909 | -0.00106 | -0.00152 | -0.00147 | -0.0003 |
| 1901.479 | -0.00105 | -0.00148 | -0.00146 | -0.0003 |
| 1900.048 | -0.00105 | -0.00147 | -0.00147 | -0.00031 |
| 1898.617 | -0.00104 | -0.00146 | -0.00146 | -0.00032 |
| 1897.186 | -0.00101 | -0.00148 | -0.00143 | -0.00032 |
| 1895.756 | -0.00099 | -0.00151 | -0.00142 | -0.00034 |
| 1894.325 | -0.00099 | -0.00154 | -0.00144 | -0.00036 |
| 1892.894 | -0.00099 | -0.00157 | -0.00147 | -0.00038 |
| 1891.463 | -0.00096 | -0.00159 | -0.00146 | -0.00037 |
| 1890.033 | -0.00091 | -0.00159 | -0.00142 | -0.00035 |
| 1888.602 | -0.00092 | -0.00158 | -0.00141 | -0.00036 |
| 1887.171 | -0.001 | -0.00156 | -0.00146 | -0.0004 |
| 1885.74 | -0.00109 | -0.00157 | -0.00151 | -0.00044 |
| 1884.31 | -0.00115 | -0.00159 | -0.00155 | -0.00045 |
| 1882.879 | -0.00118 | -0.0016 | -0.00158 | -0.00045 |
| 1881.448 | -0.00119 | -0.0016 | -0.00159 | -0.00043 |
| 1880.017 | -0.0012 | -0.0016 | -0.00158 | -0.00042 |
| 1878.587 | -0.0012 | -0.00159 | -0.00155 | -0.00041 |
| 1877.156 | -0.00119 | -0.00158 | -0.00152 | -0.00042 |
| 1875.725 | -0.00119 | -0.00157 | -0.00151 | -0.00043 |
| 1874.294 | -0.00116 | -0.00157 | -0.00151 | -0.00043 |
| 1872.863 | -0.00109 | -0.00158 | -0.00148 | -0.00043 |
| 1871.433 | -0.00093 | -0.00162 | -0.00137 | -0.00039 |
| 1870.002 | -0.00075 | -0.00166 | -0.00122 | -0.00033 |
| 1868.571 | -0.00066 | -0.00167 | -0.00113 | -0.00029 |
| 1867.14 | -0.00077 | -0.00166 | -0.00119 | -0.00031 |
| 1865.71 | -0.00098 | -0.00165 | -0.00134 | -0.00036 |
| 1864.279 | -0.00113 | -0.00165 | -0.00146 | -0.00041 |
| 1862.848 | -0.00118 | -0.00164 | -0.00151 | -0.00042 |
| 1861.417 | -0.00116 | -0.00163 | -0.00152 | -0.0004 |
| 1859.987 | -0.00113 | -0.00162 | -0.00151 | -0.00038 |
| 1858.556 | -0.00112 | -0.00159 | -0.00151 | -0.00036 |
| 1857.125 | -0.00113 | -0.00155 | -0.00153 | -0.00036 |
| 1855.694 | -0.00117 | -0.00153 | -0.00155 | -0.00039 |
| 1854.264 | -0.00121 | -0.00153 | -0.00158 | -0.00042 |
| 1852.833 | -0.00124 | -0.00156 | -0.00159 | -0.00044 |
| 1851.402 | -0.00123 | -0.0016 | -0.00159 | -0.00045 |
| 1849.971 | -0.00117 | -0.00164 | -0.00155 | -0.00043 |
| 1848.541 | -0.00106 | -0.00166 | -0.00146 | -0.00039 |
| 1847.11 | -0.00093 | -0.00168 | -0.00133 | -0.00035 |
| 1845.679 | -0.0008 | -0.00169 | -0.00118 | -0.00031 |
| 1844.248 | -0.00073 | -0.00167 | -0.00111 | -0.00029 |
| 1842.818 | -0.0008 | -0.00162 | -0.00118 | -0.00029 |
| 1841.387 | -0.00096 | -0.00159 | -0.00133 | -0.00032 |
| 1839.956 | -0.00108 | -0.00158 | -0.00145 | -0.00034 |
| 1838.525 | -0.00114 | -0.00159 | -0.0015 | -0.00036 |
| 1837.095 | -0.00115 | -0.00161 | -0.00152 | -0.00037 |
| 1835.664 | -0.00118 | -0.00162 | -0.00154 | -0.00038 |
| 1834.233 | -0.00122 | -0.00164 | -0.00154 | -0.00039 |
| 1832.802 | -0.00119 | -0.00165 | -0.00148 | -0.00037 |
| 1831.371 | -0.0011 | -0.00166 | -0.00135 | -0.00034 |
| 1829.941 | -0.001 | -0.00165 | -0.00122 | -0.00032 |
| 1828.51 | -0.00098 | -0.00163 | -0.0012 | -0.00032 |
| 1827.079 | -0.00099 | -0.00162 | -0.00124 | -0.00034 |
| 1825.648 | -0.00101 | -0.00163 | -0.00129 | -0.00037 |
| 1824.218 | -0.00107 | -0.00163 | -0.00135 | -0.0004 |
| 1822.787 | -0.00121 | -0.00162 | -0.00145 | -0.00044 |
| 1821.356 | -0.00133 | -0.00161 | -0.00155 | -0.00045 |
| 1819.925 | -0.00138 | -0.00161 | -0.00161 | -0.00042 |
| 1818.495 | -0.00136 | -0.00162 | -0.00162 | -0.00036 |
| 1817.064 | -0.00134 | -0.00163 | -0.00162 | -0.00031 |
| 1815.633 | -0.00132 | -0.00163 | -0.00161 | -0.00028 |
| 1814.202 | -0.00128 | -0.00163 | -0.00155 | -0.00026 |
| 1812.772 | -0.00119 | -0.00164 | -0.00145 | -0.00022 |
| 1811.341 | -0.00111 | -0.00164 | -0.00134 | -0.00018 |
| 1809.91 | -0.0011 | -0.00163 | -0.00131 | -0.00017 |
| 1808.479 | -0.00118 | -0.00162 | -0.00138 | -0.0002 |
| 1807.049 | -0.00127 | -0.00163 | -0.00146 | -0.00026 |
| 1805.618 | -0.0013 | -0.00164 | -0.00149 | -0.00028 |
| 1804.187 | -0.00122 | -0.00164 | -0.00142 | -0.00025 |
| 1802.756 | -0.00106 | -0.00164 | -0.00129 | -0.00019 |
| 1801.326 | -0.00094 | -0.00164 | -0.00119 | -0.00013 |
| 1799.895 | -0.00094 | -0.00163 | -0.00119 | -0.00013 |
| 1798.464 | -0.00101 | -0.00162 | -0.00123 | -0.00015 |
| 1797.033 | -0.00105 | -0.00165 | -0.00127 | -0.00017 |
| 1795.602 | -0.00105 | -0.00171 | -0.00125 | -0.00017 |
| 1794.172 | -0.001 | -0.00178 | -0.00117 | -0.00014 |
| 1792.741 | -0.00093 | -0.00177 | -0.00106 | -0.00009 |
| 1791.31 | -0.00093 | -0.00168 | -0.00104 | -0.00007 |
| 1789.879 | -0.00106 | -0.00158 | -0.00117 | -0.00011 |
| 1788.449 | -0.0012 | -0.00154 | -0.00132 | -0.00015 |
| 1787.018 | -0.00125 | -0.00154 | -0.0014 | -0.00017 |
| 1785.587 | -0.00122 | -0.00157 | -0.00139 | -0.00016 |
| 1784.156 | -0.00119 | -0.0016 | -0.00136 | -0.00014 |
| 1782.726 | -0.00118 | -0.00163 | -0.00132 | -0.00013 |
| 1781.295 | -0.00118 | -0.00162 | -0.00129 | -0.00013 |
| 1779.864 | -0.00119 | -0.0016 | -0.00128 | -0.00013 |
| 1778.433 | -0.00124 | -0.00158 | -0.0013 | -0.00014 |
| 1777.003 | -0.00128 | -0.0016 | -0.00131 | -0.00016 |
| 1775.572 | -0.00126 | -0.00166 | -0.00127 | -0.00017 |
| 1774.141 | -0.00119 | -0.00173 | -0.00116 | -0.00018 |
| 1772.71 | -0.00111 | -0.00175 | -0.00102 | -0.00017 |
| 1771.28 | -0.00104 | -0.00171 | -0.00092 | -0.0001 |
| 1769.849 | -0.00101 | -0.00163 | -0.00091 | 0 |
| 1768.418 | -0.00105 | -0.00158 | -0.001 | 0.00006 |
| 1766.987 | -0.00118 | -0.00157 | -0.00114 | 0.00005 |
| 1765.557 | -0.00132 | -0.0016 | -0.00128 | 0 |
| 1764.126 | -0.00136 | -0.00162 | -0.00131 | -0.00002 |
| 1762.695 | -0.0013 | -0.0016 | -0.00123 | 0.00001 |
| 1761.264 | -0.00123 | -0.00155 | -0.00115 | 0.00006 |
| 1759.834 | -0.00122 | -0.00152 | -0.00112 | 0.0001 |
| 1758.403 | -0.00125 | -0.00151 | -0.00113 | 0.00012 |
| 1756.972 | -0.0013 | -0.0015 | -0.00113 | 0.00013 |
| 1755.541 | -0.00136 | -0.00148 | -0.00113 | 0.00014 |
| 1754.11 | -0.00136 | -0.00148 | -0.00108 | 0.00019 |
| 1752.68 | -0.0012 | -0.00151 | -0.00093 | 0.00028 |
| 1751.249 | -0.00094 | -0.00156 | -0.0007 | 0.00036 |
| 1749.818 | -0.00077 | -0.0016 | -0.00055 | 0.00041 |
| 1748.387 | -0.00081 | -0.00159 | -0.00056 | 0.00042 |
| 1746.957 | -0.00095 | -0.00155 | -0.00064 | 0.00043 |
| 1745.526 | -0.00108 | -0.00155 | -0.00071 | 0.00045 |
| 1744.095 | -0.0012 | -0.00156 | -0.00077 | 0.00047 |
| 1742.664 | -0.00133 | -0.00157 | -0.00083 | 0.00048 |
| 1741.234 | -0.00147 | -0.00154 | -0.00087 | 0.00051 |
| 1739.803 | -0.00158 | -0.0015 | -0.00088 | 0.00055 |
| 1738.372 | -0.00163 | -0.0015 | -0.00087 | 0.00062 |
| 1736.941 | -0.00157 | -0.00153 | -0.00078 | 0.00071 |
| 1735.511 | -0.00135 | -0.00156 | -0.00054 | 0.00082 |
| 1734.08 | -0.00105 | -0.00154 | -0.00023 | 0.00097 |
| 1732.649 | -0.00094 | -0.00148 | -0.00014 | 0.00109 |
| 1731.218 | -0.00109 | -0.00143 | -0.00028 | 0.00118 |
| 1729.788 | -0.00135 | -0.00137 | -0.00047 | 0.00127 |
| 1728.357 | -0.00161 | -0.00129 | -0.0006 | 0.00137 |
| 1726.926 | -0.00181 | -0.00121 | -0.00067 | 0.00149 |
| 1725.495 | -0.0019 | -0.00114 | -0.00065 | 0.00165 |
| 1724.065 | -0.00191 | -0.00108 | -0.00058 | 0.00183 |
| 1722.634 | -0.0019 | -0.00104 | -0.00048 | 0.00198 |
| 1721.203 | -0.00185 | -0.00103 | -0.00036 | 0.0021 |
| 1719.772 | -0.00169 | -0.00107 | -0.00012 | 0.00224 |
| 1718.342 | -0.00139 | -0.0011 | 0.00028 | 0.0025 |
| 1716.911 | -0.00111 | -0.00104 | 0.00071 | 0.00288 |
| 1715.48 | -0.00112 | -0.00087 | 0.00086 | 0.0032 |
| 1714.049 | -0.00137 | -0.00069 | 0.00077 | 0.00339 |
| 1712.618 | -0.00168 | -0.00057 | 0.00064 | 0.00352 |
| 1711.188 | -0.00192 | -0.00051 | 0.00057 | 0.00368 |
| 1709.757 | -0.00203 | -0.0005 | 0.00063 | 0.00389 |
| 1708.326 | -0.00198 | -0.00047 | 0.00082 | 0.00414 |
| 1706.895 | -0.00179 | -0.00038 | 0.00117 | 0.00442 |
| 1705.465 | -0.00156 | -0.00021 | 0.00159 | 0.00472 |
| 1704.034 | -0.00144 | -0.00006 | 0.00192 | 0.00498 |
| 1702.603 | -0.00144 | -0.00001 | 0.00216 | 0.00516 |
| 1701.172 | -0.00143 | -0.00004 | 0.00238 | 0.00528 |
| 1699.742 | -0.00133 | -0.00003 | 0.00261 | 0.00541 |
| 1698.311 | -0.00121 | 0.00009 | 0.00272 | 0.00552 |
| 1696.88 | -0.0012 | 0.00026 | 0.00267 | 0.00562 |
| 1695.449 | -0.00136 | 0.00042 | 0.00255 | 0.00573 |
| 1694.019 | -0.00154 | 0.00057 | 0.00246 | 0.00589 |
| 1692.588 | -0.00158 | 0.00071 | 0.00246 | 0.00608 |
| 1691.157 | -0.00145 | 0.00082 | 0.00261 | 0.00627 |
| 1689.726 | -0.00123 | 0.00089 | 0.00284 | 0.0064 |
| 1688.296 | -0.00103 | 0.00088 | 0.00304 | 0.00643 |
| 1686.865 | -0.00085 | 0.00078 | 0.00319 | 0.00632 |
| 1685.434 | -0.00058 | 0.00069 | 0.00339 | 0.00617 |
| 1684.003 | -0.00015 | 0.00079 | 0.00372 | 0.00618 |
| 1682.573 | 0.00022 | 0.0011 | 0.00397 | 0.00641 |
| 1681.142 | 0.00038 | 0.00142 | 0.00402 | 0.00663 |
| 1679.711 | 0.00044 | 0.00166 | 0.00396 | 0.00674 |
| 1678.28 | 0.00048 | 0.00182 | 0.00392 | 0.00679 |
| 1676.849 | 0.0006 | 0.00192 | 0.004 | 0.00681 |
| 1675.419 | 0.00088 | 0.00204 | 0.00425 | 0.00685 |
| 1673.988 | 0.00127 | 0.00222 | 0.0046 | 0.00691 |
| 1672.557 | 0.00166 | 0.0024 | 0.00492 | 0.00697 |
| 1671.126 | 0.00212 | 0.00256 | 0.00521 | 0.00704 |
| 1669.696 | 0.00276 | 0.00287 | 0.00557 | 0.0072 |
| 1668.265 | 0.00342 | 0.00335 | 0.00588 | 0.00745 |
| 1666.834 | 0.00387 | 0.00378 | 0.00605 | 0.00766 |
| 1665.403 | 0.00421 | 0.00404 | 0.00621 | 0.00783 |
| 1663.973 | 0.00469 | 0.00427 | 0.00661 | 0.0081 |
| 1662.542 | 0.00546 | 0.00467 | 0.00737 | 0.00854 |
| 1661.111 | 0.00642 | 0.00525 | 0.00824 | 0.00903 |
| 1659.68 | 0.00727 | 0.00574 | 0.00881 | 0.00938 |
| 1658.25 | 0.00788 | 0.00592 | 0.00903 | 0.00953 |
| 1656.819 | 0.00834 | 0.0058 | 0.00915 | 0.00951 |
| 1655.388 | 0.00883 | 0.00559 | 0.0095 | 0.00939 |
| 1653.957 | 0.00959 | 0.00581 | 0.01048 | 0.00945 |
| 1652.527 | 0.01053 | 0.00687 | 0.01178 | 0.01001 |
| 1651.096 | 0.01133 | 0.00807 | 0.01242 | 0.01075 |
| 1649.665 | 0.01215 | 0.009 | 0.01273 | 0.01127 |
| 1648.234 | 0.0132 | 0.00991 | 0.0134 | 0.01176 |
| 1646.804 | 0.01425 | 0.01075 | 0.01456 | 0.01248 |
| 1645.373 | 0.01486 | 0.01117 | 0.01555 | 0.01339 |
| 1643.942 | 0.01512 | 0.01135 | 0.01593 | 0.01421 |
| 1642.511 | 0.01543 | 0.01171 | 0.01611 | 0.01489 |
| 1641.081 | 0.01599 | 0.01228 | 0.01654 | 0.01549 |
| 1639.65 | 0.01679 | 0.01285 | 0.01731 | 0.01599 |
| 1638.219 | 0.01777 | 0.01339 | 0.01832 | 0.01639 |
| 1636.788 | 0.01888 | 0.01418 | 0.01952 | 0.01693 |
| 1635.357 | 0.01978 | 0.01537 | 0.02062 | 0.01798 |
| 1633.927 | 0.02015 | 0.01627 | 0.02106 | 0.0192 |
| 1632.496 | 0.02017 | 0.01652 | 0.02097 | 0.02008 |
| 1631.065 | 0.02019 | 0.01646 | 0.02082 | 0.02068 |
| 1629.634 | 0.02038 | 0.01653 | 0.02095 | 0.0213 |
| 1628.204 | 0.02079 | 0.01694 | 0.02144 | 0.02201 |
| 1626.773 | 0.02126 | 0.01747 | 0.02211 | 0.02259 |
| 1625.342 | 0.0216 | 0.01778 | 0.02272 | 0.02286 |
| 1623.911 | 0.0218 | 0.01787 | 0.02323 | 0.02299 |
| 1622.481 | 0.0219 | 0.01798 | 0.02349 | 0.02316 |
| 1621.05 | 0.02187 | 0.01804 | 0.02335 | 0.02328 |
| 1619.619 | 0.0217 | 0.01785 | 0.02296 | 0.02318 |
| 1618.188 | 0.02143 | 0.01741 | 0.02259 | 0.02292 |
| 1616.758 | 0.02113 | 0.0171 | 0.02241 | 0.02276 |
| 1615.327 | 0.02088 | 0.01724 | 0.02241 | 0.0229 |
| 1613.896 | 0.02066 | 0.01758 | 0.02245 | 0.02311 |
| 1612.465 | 0.02046 | 0.01778 | 0.02243 | 0.0231 |
| 1611.035 | 0.02035 | 0.01783 | 0.02238 | 0.02293 |
| 1609.604 | 0.02045 | 0.0179 | 0.0224 | 0.02287 |
| 1608.173 | 0.02068 | 0.01806 | 0.02248 | 0.02306 |
| 1606.742 | 0.02093 | 0.01828 | 0.02261 | 0.02337 |
| 1605.312 | 0.02119 | 0.01855 | 0.02283 | 0.02368 |
| 1603.881 | 0.02157 | 0.01892 | 0.0232 | 0.02403 |
| 1602.45 | 0.02208 | 0.0194 | 0.02369 | 0.02452 |
| 1601.019 | 0.02267 | 0.01998 | 0.02419 | 0.02516 |
| 1599.589 | 0.0233 | 0.02062 | 0.02465 | 0.02583 |
| 1598.158 | 0.02399 | 0.02132 | 0.02515 | 0.02649 |
| 1596.727 | 0.02476 | 0.02209 | 0.02583 | 0.02714 |
| 1595.296 | 0.02562 | 0.02294 | 0.0267 | 0.02784 |
| 1593.865 | 0.02654 | 0.02383 | 0.02766 | 0.02861 |
| 1592.435 | 0.02751 | 0.02469 | 0.02858 | 0.0294 |
| 1591.004 | 0.02852 | 0.02549 | 0.02942 | 0.03017 |
| 1589.573 | 0.02949 | 0.02625 | 0.03019 | 0.03094 |
| 1588.142 | 0.03036 | 0.02696 | 0.03089 | 0.03169 |
| 1586.712 | 0.03109 | 0.02762 | 0.03152 | 0.03241 |
| 1585.281 | 0.03165 | 0.02818 | 0.03208 | 0.03304 |
| 1583.85 | 0.03202 | 0.02861 | 0.03255 | 0.03352 |
| 1582.419 | 0.03219 | 0.02888 | 0.03292 | 0.03384 |
| 1580.989 | 0.03223 | 0.029 | 0.03317 | 0.03398 |
| 1579.558 | 0.03221 | 0.02898 | 0.03331 | 0.03395 |
| 1578.127 | 0.03218 | 0.02883 | 0.03336 | 0.03379 |
| 1576.696 | 0.03204 | 0.02859 | 0.03322 | 0.03358 |
| 1575.266 | 0.0317 | 0.02839 | 0.03281 | 0.03342 |
| 1573.835 | 0.03132 | 0.02827 | 0.0323 | 0.0333 |
| 1572.404 | 0.03099 | 0.02811 | 0.03192 | 0.03315 |
| 1570.973 | 0.03065 | 0.02783 | 0.03164 | 0.03287 |
| 1569.543 | 0.03017 | 0.02738 | 0.03132 | 0.0324 |
| 1568.112 | 0.02958 | 0.02686 | 0.03082 | 0.03183 |
| 1566.681 | 0.02901 | 0.02634 | 0.0302 | 0.03122 |
| 1565.25 | 0.02855 | 0.02584 | 0.02959 | 0.03064 |
| 1563.82 | 0.02822 | 0.02537 | 0.02912 | 0.03013 |
| 1562.389 | 0.02801 | 0.02493 | 0.02887 | 0.02974 |
| 1560.958 | 0.02788 | 0.02444 | 0.02884 | 0.02937 |
| 1559.527 | 0.02765 | 0.02387 | 0.02876 | 0.02881 |
| 1558.097 | 0.02717 | 0.0234 | 0.02816 | 0.02809 |
| 1556.666 | 0.02667 | 0.02313 | 0.02732 | 0.02757 |
| 1555.235 | 0.02627 | 0.02287 | 0.02664 | 0.02725 |
| 1553.804 | 0.02593 | 0.02255 | 0.02609 | 0.02693 |
| 1552.373 | 0.02563 | 0.02218 | 0.0256 | 0.02656 |
| 1550.943 | 0.02528 | 0.02177 | 0.02512 | 0.02608 |
| 1549.512 | 0.02482 | 0.02133 | 0.02457 | 0.02551 |
| 1548.081 | 0.02433 | 0.0209 | 0.02402 | 0.02498 |
| 1546.65 | 0.02397 | 0.02043 | 0.02358 | 0.02455 |
| 1545.22 | 0.02373 | 0.01987 | 0.02325 | 0.02415 |
| 1543.789 | 0.02342 | 0.01919 | 0.02291 | 0.02362 |
| 1542.358 | 0.02292 | 0.01848 | 0.02245 | 0.02293 |
| 1540.927 | 0.02215 | 0.01787 | 0.02175 | 0.02205 |
| 1539.497 | 0.02115 | 0.0174 | 0.02075 | 0.0211 |
| 1538.066 | 0.02026 | 0.01705 | 0.01985 | 0.02044 |
| 1536.635 | 0.01966 | 0.01669 | 0.01925 | 0.02 |
| 1535.204 | 0.01919 | 0.01625 | 0.0188 | 0.01955 |
| 1533.774 | 0.01866 | 0.01571 | 0.01827 | 0.01892 |
| 1532.343 | 0.01798 | 0.01515 | 0.01758 | 0.01819 |
| 1530.912 | 0.01729 | 0.01464 | 0.01689 | 0.01756 |
| 1529.481 | 0.01674 | 0.01413 | 0.01633 | 0.01704 |
| 1528.051 | 0.01629 | 0.01357 | 0.01586 | 0.0165 |
| 1526.62 | 0.0158 | 0.01297 | 0.01534 | 0.01588 |
| 1525.189 | 0.01522 | 0.01235 | 0.01478 | 0.01523 |
| 1523.758 | 0.01462 | 0.0117 | 0.01425 | 0.01461 |
| 1522.328 | 0.01401 | 0.01103 | 0.01373 | 0.01398 |
| 1520.897 | 0.01334 | 0.01042 | 0.01311 | 0.01331 |
| 1519.466 | 0.0127 | 0.00995 | 0.01248 | 0.01272 |
| 1518.035 | 0.01211 | 0.00956 | 0.01191 | 0.01222 |
| 1516.604 | 0.0115 | 0.00918 | 0.01131 | 0.01172 |
| 1515.174 | 0.01084 | 0.00879 | 0.01063 | 0.01119 |
| 1513.743 | 0.01022 | 0.0084 | 0.00997 | 0.01069 |
| 1512.312 | 0.00979 | 0.00797 | 0.00949 | 0.01027 |
| 1510.881 | 0.00958 | 0.00753 | 0.00927 | 0.00993 |
| 1509.451 | 0.00952 | 0.00708 | 0.00929 | 0.00963 |
| 1508.02 | 0.00922 | 0.00658 | 0.00924 | 0.00917 |
| 1506.589 | 0.00812 | 0.00603 | 0.00849 | 0.00832 |
| 1505.158 | 0.00673 | 0.00559 | 0.00722 | 0.00751 |
| 1503.728 | 0.00585 | 0.0053 | 0.00626 | 0.00706 |
| 1502.297 | 0.0054 | 0.00505 | 0.00571 | 0.00683 |
| 1500.866 | 0.00513 | 0.00479 | 0.00542 | 0.00662 |
| 1499.435 | 0.0049 | 0.00453 | 0.00526 | 0.00638 |
| 1498.005 | 0.00463 | 0.00428 | 0.00511 | 0.0061 |
| 1496.574 | 0.00428 | 0.00409 | 0.00485 | 0.00579 |
| 1495.143 | 0.00389 | 0.00391 | 0.0045 | 0.00548 |
| 1493.712 | 0.00362 | 0.0037 | 0.00423 | 0.00524 |
| 1492.282 | 0.00358 | 0.00345 | 0.00414 | 0.00508 |
| 1490.851 | 0.00368 | 0.00318 | 0.00418 | 0.00497 |
| 1489.42 | 0.00366 | 0.003 | 0.00413 | 0.00483 |
| 1487.989 | 0.00334 | 0.00293 | 0.00386 | 0.00464 |
| 1486.559 | 0.00286 | 0.00291 | 0.00347 | 0.00445 |
| 1485.128 | 0.00246 | 0.00286 | 0.00314 | 0.00429 |
| 1483.697 | 0.0022 | 0.00278 | 0.00293 | 0.00415 |
| 1482.266 | 0.00203 | 0.00268 | 0.00279 | 0.00403 |
| 1480.836 | 0.00192 | 0.00258 | 0.00269 | 0.00393 |
| 1479.405 | 0.00189 | 0.00247 | 0.00262 | 0.00385 |
| 1477.974 | 0.00197 | 0.00233 | 0.00262 | 0.00379 |
| 1476.543 | 0.00216 | 0.00219 | 0.00273 | 0.00378 |
| 1475.112 | 0.00238 | 0.00208 | 0.00291 | 0.00382 |
| 1473.682 | 0.00246 | 0.00204 | 0.00303 | 0.00382 |
| 1472.251 | 0.00225 | 0.00207 | 0.00294 | 0.00372 |
| 1470.82 | 0.00185 | 0.00215 | 0.00266 | 0.00362 |
| 1469.389 | 0.00156 | 0.00224 | 0.00244 | 0.00361 |
| 1467.959 | 0.00151 | 0.00234 | 0.00241 | 0.0037 |
| 1466.528 | 0.00169 | 0.00249 | 0.00261 | 0.00388 |
| 1465.097 | 0.00206 | 0.00273 | 0.00294 | 0.00414 |
| 1463.666 | 0.0025 | 0.00304 | 0.00327 | 0.00446 |
| 1462.236 | 0.00298 | 0.00334 | 0.00358 | 0.00481 |
| 1460.805 | 0.00357 | 0.00361 | 0.00402 | 0.00518 |
| 1459.374 | 0.00435 | 0.00389 | 0.00472 | 0.00562 |
| 1457.943 | 0.00533 | 0.00431 | 0.00564 | 0.0062 |
| 1456.513 | 0.00601 | 0.00485 | 0.00626 | 0.00677 |
| 1455.082 | 0.00604 | 0.00521 | 0.00629 | 0.00706 |
| 1453.651 | 0.00575 | 0.00529 | 0.00604 | 0.00707 |
| 1452.22 | 0.0054 | 0.0052 | 0.00581 | 0.00695 |
| 1450.79 | 0.00509 | 0.00503 | 0.00563 | 0.00677 |
| 1449.359 | 0.00479 | 0.0048 | 0.00545 | 0.00656 |
| 1447.928 | 0.00444 | 0.00455 | 0.0052 | 0.00632 |
| 1446.497 | 0.00403 | 0.00433 | 0.00487 | 0.00607 |
| 1445.067 | 0.00363 | 0.00416 | 0.00454 | 0.00587 |
| 1443.636 | 0.00335 | 0.00405 | 0.0043 | 0.00572 |
| 1442.205 | 0.00318 | 0.00395 | 0.00416 | 0.00561 |
| 1440.774 | 0.0031 | 0.00383 | 0.00409 | 0.00551 |
| 1439.344 | 0.0031 | 0.00366 | 0.00407 | 0.00541 |
| 1437.913 | 0.00317 | 0.00348 | 0.00409 | 0.00532 |
| 1436.482 | 0.00325 | 0.00335 | 0.0041 | 0.00524 |
| 1435.051 | 0.00322 | 0.00332 | 0.00406 | 0.00522 |
| 1433.62 | 0.00312 | 0.00335 | 0.00401 | 0.00526 |
| 1432.19 | 0.00307 | 0.00343 | 0.00407 | 0.00538 |
| 1430.759 | 0.00314 | 0.00361 | 0.00429 | 0.00561 |
| 1429.328 | 0.00332 | 0.0039 | 0.00463 | 0.00596 |
| 1427.897 | 0.00358 | 0.00424 | 0.005 | 0.00635 |
| 1426.467 | 0.00389 | 0.00456 | 0.0054 | 0.00675 |
| 1425.036 | 0.00426 | 0.00488 | 0.00584 | 0.00719 |
| 1423.605 | 0.00466 | 0.00523 | 0.0063 | 0.00765 |
| 1422.174 | 0.00511 | 0.00561 | 0.00679 | 0.00814 |
| 1420.744 | 0.00572 | 0.00606 | 0.00744 | 0.00872 |
| 1419.313 | 0.00661 | 0.00672 | 0.00833 | 0.0095 |
| 1417.882 | 0.00754 | 0.00757 | 0.00926 | 0.01042 |
| 1416.451 | 0.00826 | 0.00843 | 0.00998 | 0.01124 |
| 1415.021 | 0.0089 | 0.00922 | 0.01061 | 0.01197 |
| 1413.59 | 0.00971 | 0.01 | 0.01134 | 0.01276 |
| 1412.159 | 0.01074 | 0.01082 | 0.01225 | 0.01365 |
| 1410.728 | 0.01187 | 0.0117 | 0.01321 | 0.01459 |
| 1409.298 | 0.01296 | 0.01254 | 0.0141 | 0.01548 |
| 1407.867 | 0.01392 | 0.01325 | 0.01487 | 0.01622 |
| 1406.436 | 0.01479 | 0.01382 | 0.01553 | 0.01681 |
| 1405.005 | 0.0155 | 0.01428 | 0.01604 | 0.01726 |
| 1403.575 | 0.01594 | 0.0146 | 0.01628 | 0.0175 |
| 1402.144 | 0.01613 | 0.0147 | 0.01631 | 0.01755 |
| 1400.713 | 0.01614 | 0.01455 | 0.0162 | 0.01742 |
| 1399.282 | 0.01598 | 0.01415 | 0.01595 | 0.01709 |
| 1397.852 | 0.01557 | 0.01356 | 0.01546 | 0.01654 |
| 1396.421 | 0.01486 | 0.01281 | 0.01471 | 0.0158 |
| 1394.99 | 0.01387 | 0.01196 | 0.01371 | 0.01489 |
| 1393.559 | 0.01273 | 0.01112 | 0.01261 | 0.01392 |
| 1392.128 | 0.01169 | 0.01039 | 0.01164 | 0.01302 |
| 1390.698 | 0.01088 | 0.00973 | 0.01087 | 0.01224 |
| 1389.267 | 0.01021 | 0.00909 | 0.01023 | 0.01155 |
| 1387.836 | 0.00955 | 0.00847 | 0.0096 | 0.01089 |
| 1386.405 | 0.00881 | 0.00793 | 0.00892 | 0.01027 |
| 1384.975 | 0.00808 | 0.00749 | 0.00824 | 0.00974 |
| 1383.544 | 0.00748 | 0.0071 | 0.00767 | 0.00929 |
| 1382.113 | 0.00706 | 0.00674 | 0.00723 | 0.00889 |
| 1380.682 | 0.00675 | 0.00637 | 0.00689 | 0.00851 |
| 1379.252 | 0.00646 | 0.00598 | 0.0066 | 0.00812 |
| 1377.821 | 0.00614 | 0.00559 | 0.00633 | 0.00774 |
| 1376.39 | 0.00583 | 0.00525 | 0.0061 | 0.00741 |
| 1374.959 | 0.00555 | 0.00499 | 0.00587 | 0.00714 |
| 1373.529 | 0.00524 | 0.00478 | 0.00558 | 0.00689 |
| 1372.098 | 0.00492 | 0.00459 | 0.00527 | 0.00664 |
| 1370.667 | 0.00466 | 0.00441 | 0.005 | 0.00644 |
| 1369.236 | 0.00446 | 0.00425 | 0.00481 | 0.00628 |
| 1367.806 | 0.00426 | 0.00412 | 0.00464 | 0.00613 |
| 1366.375 | 0.00411 | 0.004 | 0.0045 | 0.00598 |
| 1364.944 | 0.00407 | 0.00389 | 0.00446 | 0.00588 |
| 1363.513 | 0.0041 | 0.00377 | 0.00447 | 0.0058 |
| 1362.083 | 0.00404 | 0.00365 | 0.00441 | 0.00569 |
| 1360.652 | 0.00382 | 0.00355 | 0.00421 | 0.00553 |
| 1359.221 | 0.00355 | 0.00349 | 0.00398 | 0.00539 |
| 1357.79 | 0.00336 | 0.00344 | 0.00383 | 0.00531 |
| 1356.359 | 0.00328 | 0.00341 | 0.00379 | 0.00529 |
| 1354.929 | 0.00326 | 0.00341 | 0.00382 | 0.00533 |
| 1353.498 | 0.00328 | 0.00344 | 0.00388 | 0.0054 |
| 1352.067 | 0.0033 | 0.00349 | 0.00393 | 0.0055 |
| 1350.636 | 0.00334 | 0.00354 | 0.00396 | 0.00557 |
| 1349.206 | 0.0034 | 0.0036 | 0.00398 | 0.0056 |
| 1347.775 | 0.00349 | 0.00365 | 0.00402 | 0.00561 |
| 1346.344 | 0.00358 | 0.00371 | 0.00407 | 0.0056 |
| 1344.913 | 0.00367 | 0.00372 | 0.00413 | 0.00559 |
| 1343.483 | 0.0038 | 0.0037 | 0.00422 | 0.00559 |
| 1342.052 | 0.00403 | 0.00367 | 0.00435 | 0.00564 |
| 1340.621 | 0.00434 | 0.00368 | 0.00454 | 0.00573 |
| 1339.19 | 0.00455 | 0.00374 | 0.00468 | 0.00581 |
| 1337.76 | 0.00451 | 0.00382 | 0.00467 | 0.00582 |
| 1336.329 | 0.00429 | 0.00387 | 0.00454 | 0.00579 |
| 1334.898 | 0.00411 | 0.00392 | 0.00442 | 0.00578 |
| 1333.467 | 0.00406 | 0.00398 | 0.00442 | 0.00584 |
| 1332.037 | 0.00413 | 0.00407 | 0.00453 | 0.00597 |
| 1330.606 | 0.00425 | 0.00417 | 0.00464 | 0.00612 |
| 1329.175 | 0.00436 | 0.00426 | 0.00474 | 0.00626 |
| 1327.744 | 0.00449 | 0.00438 | 0.00483 | 0.00639 |
| 1326.314 | 0.00464 | 0.00449 | 0.00496 | 0.00651 |
| 1324.883 | 0.0048 | 0.00459 | 0.0051 | 0.00661 |
| 1323.452 | 0.00493 | 0.00465 | 0.00525 | 0.00669 |
| 1322.021 | 0.00503 | 0.0047 | 0.0054 | 0.00675 |
| 1320.591 | 0.00507 | 0.00474 | 0.00554 | 0.00681 |
| 1319.16 | 0.00508 | 0.00476 | 0.00563 | 0.00686 |
| 1317.729 | 0.00506 | 0.00475 | 0.00566 | 0.0069 |
| 1316.298 | 0.00503 | 0.00471 | 0.00563 | 0.00691 |
| 1314.867 | 0.005 | 0.00468 | 0.00556 | 0.00688 |
| 1313.437 | 0.00495 | 0.00463 | 0.00546 | 0.0068 |
| 1312.006 | 0.00483 | 0.00453 | 0.00533 | 0.00666 |
| 1310.575 | 0.00464 | 0.00439 | 0.00516 | 0.00651 |
| 1309.144 | 0.00444 | 0.00424 | 0.00499 | 0.00636 |
| 1307.714 | 0.00427 | 0.00411 | 0.00481 | 0.0062 |
| 1306.283 | 0.00411 | 0.00399 | 0.00464 | 0.00605 |
| 1304.852 | 0.00396 | 0.00387 | 0.00449 | 0.00593 |
| 1303.421 | 0.00384 | 0.00377 | 0.00437 | 0.00586 |
| 1301.991 | 0.00378 | 0.00372 | 0.00433 | 0.00583 |
| 1300.56 | 0.00374 | 0.0037 | 0.00433 | 0.00578 |
| 1299.129 | 0.00371 | 0.00367 | 0.0043 | 0.00568 |
| 1297.698 | 0.00366 | 0.00361 | 0.00423 | 0.00555 |
| 1296.268 | 0.0036 | 0.00354 | 0.00415 | 0.00545 |
| 1294.837 | 0.00356 | 0.0035 | 0.00412 | 0.00542 |
| 1293.406 | 0.00352 | 0.0035 | 0.00413 | 0.00545 |
| 1291.975 | 0.00347 | 0.00349 | 0.00417 | 0.00551 |
| 1290.545 | 0.0034 | 0.00343 | 0.00418 | 0.00555 |
| 1289.114 | 0.00333 | 0.00338 | 0.00417 | 0.00557 |
| 1287.683 | 0.00328 | 0.00336 | 0.00414 | 0.00559 |
| 1286.252 | 0.00322 | 0.00334 | 0.00411 | 0.00561 |
| 1284.822 | 0.00317 | 0.00331 | 0.00409 | 0.00561 |
| 1283.391 | 0.00315 | 0.00331 | 0.00409 | 0.00561 |
| 1281.96 | 0.00318 | 0.00336 | 0.00409 | 0.00563 |
| 1280.529 | 0.00327 | 0.00343 | 0.0041 | 0.00568 |
| 1279.099 | 0.00336 | 0.00348 | 0.00412 | 0.00574 |
| 1277.668 | 0.00341 | 0.00349 | 0.00414 | 0.0058 |
| 1276.237 | 0.00342 | 0.00346 | 0.00416 | 0.00586 |
| 1274.806 | 0.00344 | 0.00345 | 0.0042 | 0.00591 |
| 1273.375 | 0.0035 | 0.00348 | 0.00426 | 0.00593 |
| 1271.945 | 0.00357 | 0.00354 | 0.00431 | 0.00591 |
| 1270.514 | 0.00359 | 0.0036 | 0.00429 | 0.00585 |
| 1269.083 | 0.00352 | 0.0036 | 0.00422 | 0.00578 |
| 1267.652 | 0.0034 | 0.00352 | 0.00412 | 0.0057 |
| 1266.222 | 0.00324 | 0.00337 | 0.004 | 0.00559 |
| 1264.791 | 0.00304 | 0.00321 | 0.00387 | 0.00545 |
| 1263.36 | 0.00284 | 0.00308 | 0.00372 | 0.00529 |
| 1261.929 | 0.00269 | 0.00296 | 0.00358 | 0.00515 |
| 1260.499 | 0.00257 | 0.00281 | 0.00345 | 0.00505 |
| 1259.068 | 0.00246 | 0.00266 | 0.00332 | 0.00496 |
| 1257.637 | 0.00234 | 0.00253 | 0.00318 | 0.00486 |
| 1256.206 | 0.00223 | 0.00244 | 0.00305 | 0.00476 |
| 1254.776 | 0.00213 | 0.00237 | 0.00294 | 0.00461 |
| 1253.345 | 0.00205 | 0.00231 | 0.00283 | 0.00444 |
| 1251.914 | 0.00198 | 0.00224 | 0.00273 | 0.00429 |
| 1250.483 | 0.00191 | 0.00218 | 0.00263 | 0.0042 |
| 1249.053 | 0.00188 | 0.00213 | 0.00257 | 0.00417 |
| 1247.622 | 0.00186 | 0.00207 | 0.00254 | 0.00416 |
| 1246.191 | 0.00184 | 0.00201 | 0.00253 | 0.00415 |
| 1244.76 | 0.00182 | 0.00197 | 0.00253 | 0.00415 |
| 1243.33 | 0.00179 | 0.00196 | 0.00251 | 0.00416 |
| 1241.899 | 0.00177 | 0.00197 | 0.00248 | 0.00418 |
| 1240.468 | 0.00177 | 0.00196 | 0.00247 | 0.00419 |
| 1239.037 | 0.00177 | 0.00192 | 0.00246 | 0.00417 |
| 1237.606 | 0.00177 | 0.00187 | 0.00243 | 0.00409 |
| 1236.176 | 0.00179 | 0.00183 | 0.00241 | 0.00404 |
| 1234.745 | 0.00184 | 0.00186 | 0.00244 | 0.00405 |
| 1233.314 | 0.00189 | 0.00193 | 0.00252 | 0.0041 |
| 1231.883 | 0.00188 | 0.00197 | 0.00256 | 0.00412 |
| 1230.453 | 0.0018 | 0.00193 | 0.00251 | 0.00406 |
| 1229.022 | 0.00168 | 0.00182 | 0.00239 | 0.00394 |
| 1227.591 | 0.00156 | 0.00169 | 0.00226 | 0.00382 |
| 1226.16 | 0.00146 | 0.00158 | 0.00218 | 0.00374 |
| 1224.73 | 0.00138 | 0.0015 | 0.00213 | 0.00369 |
| 1223.299 | 0.00128 | 0.00143 | 0.00207 | 0.00367 |
| 1221.868 | 0.00116 | 0.0014 | 0.002 | 0.00364 |
| 1220.437 | 0.00104 | 0.00138 | 0.00193 | 0.00357 |
| 1219.007 | 0.00096 | 0.00134 | 0.00186 | 0.00348 |
| 1217.576 | 0.0009 | 0.00127 | 0.00179 | 0.00338 |
| 1216.145 | 0.00088 | 0.00119 | 0.00173 | 0.00332 |
| 1214.714 | 0.00088 | 0.00114 | 0.00173 | 0.00331 |
| 1213.284 | 0.00085 | 0.00112 | 0.00174 | 0.00335 |
| 1211.853 | 0.00078 | 0.0011 | 0.00175 | 0.00338 |
| 1210.422 | 0.0007 | 0.00108 | 0.00172 | 0.00341 |
| 1208.991 | 0.00066 | 0.00109 | 0.00169 | 0.00342 |
| 1207.561 | 0.00066 | 0.00111 | 0.00167 | 0.00342 |
| 1206.13 | 0.00068 | 0.00113 | 0.00167 | 0.00342 |
| 1204.699 | 0.00067 | 0.00113 | 0.00167 | 0.00342 |
| 1203.268 | 0.00062 | 0.00111 | 0.00164 | 0.00339 |
| 1201.838 | 0.00057 | 0.00108 | 0.00159 | 0.00334 |
| 1200.407 | 0.00055 | 0.00104 | 0.00155 | 0.00328 |
| 1198.976 | 0.00056 | 0.00102 | 0.00153 | 0.00324 |
| 1197.545 | 0.00057 | 0.001 | 0.00151 | 0.00322 |
| 1196.114 | 0.00057 | 0.001 | 0.00151 | 0.00323 |
| 1194.684 | 0.00058 | 0.00101 | 0.00154 | 0.00327 |
| 1193.253 | 0.00062 | 0.00103 | 0.00158 | 0.00333 |
| 1191.822 | 0.00069 | 0.00104 | 0.00164 | 0.00342 |
| 1190.391 | 0.00074 | 0.00104 | 0.00172 | 0.00351 |
| 1188.961 | 0.00077 | 0.00101 | 0.00179 | 0.00354 |
| 1187.53 | 0.00079 | 0.00099 | 0.00184 | 0.00351 |
| 1186.099 | 0.00081 | 0.00101 | 0.00184 | 0.00346 |
| 1184.668 | 0.0008 | 0.00103 | 0.00179 | 0.0034 |
| 1183.238 | 0.00077 | 0.00101 | 0.0017 | 0.00333 |
| 1181.807 | 0.00072 | 0.00095 | 0.0016 | 0.00326 |
| 1180.376 | 0.00067 | 0.00091 | 0.00155 | 0.00321 |
| 1178.945 | 0.00062 | 0.00089 | 0.00152 | 0.00318 |
| 1177.515 | 0.00056 | 0.00084 | 0.00149 | 0.00317 |
| 1176.084 | 0.00053 | 0.00077 | 0.00142 | 0.00315 |
| 1174.653 | 0.00048 | 0.00069 | 0.00131 | 0.00312 |
| 1173.222 | 0.00038 | 0.00062 | 0.00122 | 0.00303 |
| 1171.792 | 0.00026 | 0.00055 | 0.00116 | 0.00292 |
| 1170.361 | 0.00015 | 0.00048 | 0.00112 | 0.00283 |
| 1168.93 | 0.00008 | 0.00043 | 0.00109 | 0.00277 |
| 1167.499 | 0.00002 | 0.00042 | 0.00105 | 0.00274 |
| 1166.069 | -0.00001 | 0.00045 | 0.00103 | 0.00274 |
| 1164.638 | -0.00002 | 0.00044 | 0.00098 | 0.00272 |
| 1163.207 | -0.00006 | 0.00039 | 0.00089 | 0.00269 |
| 1161.776 | -0.00016 | 0.00033 | 0.00075 | 0.00261 |
| 1160.346 | -0.0003 | 0.00026 | 0.00062 | 0.00251 |
| 1158.915 | -0.00043 | 0.00015 | 0.00052 | 0.00239 |
| 1157.484 | -0.00053 | 0 | 0.00046 | 0.00228 |
| 1156.053 | -0.00057 | -0.00014 | 0.00043 | 0.0022 |
| 1154.622 | -0.00058 | -0.00018 | 0.00038 | 0.00213 |
| 1153.192 | -0.00058 | -0.00013 | 0.00032 | 0.00209 |
| 1151.761 | -0.00056 | -0.00005 | 0.00029 | 0.00208 |
| 1150.33 | -0.00049 | -0.00001 | 0.00034 | 0.00214 |
| 1148.899 | -0.0004 | 0.00001 | 0.00043 | 0.00224 |
| 1147.469 | -0.00031 | 0.00006 | 0.00053 | 0.00238 |
| 1146.038 | -0.00023 | 0.00016 | 0.00062 | 0.00251 |
| 1144.607 | -0.00012 | 0.00029 | 0.00071 | 0.00261 |
| 1143.176 | 0.00001 | 0.00042 | 0.00079 | 0.00266 |
| 1141.746 | 0.00014 | 0.00051 | 0.00087 | 0.00272 |
| 1140.315 | 0.00026 | 0.00057 | 0.00097 | 0.00284 |
| 1138.884 | 0.00039 | 0.00063 | 0.0011 | 0.003 |
| 1137.453 | 0.00052 | 0.00073 | 0.00122 | 0.00315 |
| 1136.023 | 0.00065 | 0.00085 | 0.00132 | 0.0033 |
| 1134.592 | 0.00077 | 0.001 | 0.0014 | 0.00345 |
| 1133.161 | 0.00085 | 0.00112 | 0.00149 | 0.00355 |
| 1131.73 | 0.00085 | 0.00119 | 0.00157 | 0.00358 |
| 1130.3 | 0.00081 | 0.00116 | 0.00161 | 0.00353 |
| 1128.869 | 0.00077 | 0.00108 | 0.00159 | 0.00345 |
| 1127.438 | 0.00074 | 0.00102 | 0.00155 | 0.00342 |
| 1126.007 | 0.00071 | 0.001 | 0.00151 | 0.00344 |
| 1124.577 | 0.00064 | 0.00098 | 0.00145 | 0.00346 |
| 1123.146 | 0.00051 | 0.00091 | 0.00138 | 0.00343 |
| 1121.715 | 0.00036 | 0.00077 | 0.00129 | 0.00335 |
| 1120.284 | 0.00021 | 0.00066 | 0.00122 | 0.00325 |
| 1118.854 | 0.00012 | 0.00062 | 0.00115 | 0.00314 |
| 1117.423 | 0.00003 | 0.00062 | 0.00109 | 0.00303 |
| 1115.992 | -0.00006 | 0.0006 | 0.00101 | 0.00292 |
| 1114.561 | -0.00014 | 0.00053 | 0.00092 | 0.00283 |
| 1113.13 | -0.00018 | 0.00044 | 0.00082 | 0.00277 |
| 1111.7 | -0.00022 | 0.00036 | 0.00073 | 0.00272 |
| 1110.269 | -0.0003 | 0.00026 | 0.00063 | 0.00267 |
| 1108.838 | -0.00041 | 0.00014 | 0.00054 | 0.0026 |
| 1107.407 | -0.00048 | 0.00004 | 0.00045 | 0.00254 |
| 1105.977 | -0.0005 | -0.00001 | 0.00037 | 0.00251 |
| 1104.546 | -0.00052 | -0.00004 | 0.0003 | 0.00249 |
| 1103.115 | -0.0006 | -0.00008 | 0.00025 | 0.00247 |
| 1101.684 | -0.0007 | -0.0001 | 0.00025 | 0.00243 |
| 1100.254 | -0.00078 | -0.00005 | 0.00027 | 0.00234 |
| 1098.823 | -0.00082 | 0 | 0.00027 | 0.00223 |
| 1097.392 | -0.00084 | 0.00001 | 0.00024 | 0.00214 |
| 1095.961 | -0.00082 | -0.00001 | 0.00024 | 0.0021 |
| 1094.531 | -0.00071 | 0.00001 | 0.0003 | 0.00216 |
| 1093.1 | -0.00057 | 0.00009 | 0.00039 | 0.0023 |
| 1091.669 | -0.00046 | 0.00017 | 0.00044 | 0.00246 |
| 1090.238 | -0.00044 | 0.00018 | 0.00044 | 0.00254 |
| 1088.808 | -0.00047 | 0.00012 | 0.00041 | 0.00251 |
| 1087.377 | -0.00046 | 0.00005 | 0.00042 | 0.00244 |
| 1085.946 | -0.00041 | 0.00001 | 0.00047 | 0.0024 |
| 1084.515 | -0.00034 | 0.00001 | 0.0005 | 0.00243 |
| 1083.085 | -0.0003 | 0.00005 | 0.00048 | 0.00248 |
| 1081.654 | -0.00029 | 0.0001 | 0.00041 | 0.00249 |
| 1080.223 | -0.00032 | 0.00013 | 0.00032 | 0.00245 |
| 1078.792 | -0.00041 | 0.00013 | 0.00027 | 0.00239 |
| 1077.361 | -0.00055 | 0.00009 | 0.00027 | 0.00233 |
| 1075.931 | -0.00067 | 0.00005 | 0.00031 | 0.0023 |
| 1074.5 | -0.00069 | 0.00003 | 0.00038 | 0.0023 |
| 1073.069 | -0.00062 | 0.00003 | 0.00046 | 0.00234 |
| 1071.638 | -0.00054 | 0.00005 | 0.00051 | 0.00236 |
| 1070.208 | -0.00052 | 0.00005 | 0.00051 | 0.00235 |
| 1068.777 | -0.00054 | 0.00002 | 0.00049 | 0.00232 |
| 1067.346 | -0.00056 | -0.00006 | 0.00048 | 0.0023 |
| 1065.915 | -0.00057 | -0.00016 | 0.00048 | 0.00224 |
| 1064.485 | -0.00057 | -0.0002 | 0.00049 | 0.00216 |
| 1063.054 | -0.00056 | -0.00015 | 0.00048 | 0.00212 |
| 1061.623 | -0.00056 | -0.00007 | 0.00043 | 0.00216 |
| 1060.192 | -0.00059 | -0.00003 | 0.00032 | 0.0022 |
| 1058.762 | -0.00063 | -0.00004 | 0.0002 | 0.00222 |
| 1057.331 | -0.00066 | -0.00004 | 0.00016 | 0.00221 |
| 1055.9 | -0.00065 | -0.00001 | 0.00023 | 0.0022 |
| 1054.469 | -0.00058 | 0.00005 | 0.00035 | 0.00221 |
| 1053.039 | -0.00047 | 0.00008 | 0.00045 | 0.00226 |
| 1051.608 | -0.00038 | 0.00007 | 0.00052 | 0.00235 |
| 1050.177 | -0.00035 | 0.00003 | 0.00058 | 0.00245 |
| 1048.746 | -0.00037 | 0 | 0.00063 | 0.00252 |
| 1047.316 | -0.00042 | 0.00001 | 0.00067 | 0.00254 |
| 1045.885 | -0.00046 | 0.00003 | 0.00065 | 0.00255 |
| 1044.454 | -0.00047 | 0 | 0.0006 | 0.00257 |
| 1043.023 | -0.00044 | -0.00004 | 0.00057 | 0.00265 |
| 1041.593 | -0.00035 | -0.00003 | 0.00063 | 0.00279 |
| 1040.162 | -0.00029 | 0.00004 | 0.00076 | 0.0029 |
| 1038.731 | -0.00031 | 0.00008 | 0.00082 | 0.00293 |
| 1037.3 | -0.00041 | 0.00006 | 0.00073 | 0.00287 |
| 1035.869 | -0.00048 | 0.00003 | 0.0006 | 0.0028 |
| 1034.439 | -0.00044 | 0.00006 | 0.00058 | 0.00279 |
| 1033.008 | -0.00031 | 0.00017 | 0.00072 | 0.00285 |
| 1031.577 | -0.00015 | 0.00028 | 0.00092 | 0.00294 |
| 1030.146 | -0.00007 | 0.00031 | 0.00106 | 0.00297 |
| 1028.716 | -0.00013 | 0.00026 | 0.00107 | 0.00291 |
| 1027.285 | -0.00029 | 0.00015 | 0.00098 | 0.00282 |
| 1025.854 | -0.00045 | 0.00003 | 0.00084 | 0.00272 |
| 1024.423 | -0.00056 | -0.00008 | 0.0007 | 0.00261 |
| 1022.993 | -0.00061 | -0.00015 | 0.00061 | 0.00251 |
| 1021.562 | -0.00062 | -0.00017 | 0.00059 | 0.00245 |
| 1020.131 | -0.00061 | -0.00014 | 0.00063 | 0.00247 |
| 1018.7 | -0.00061 | -0.00006 | 0.00069 | 0.00254 |
| 1017.27 | -0.00062 | 0.00001 | 0.00073 | 0.00258 |
| 1015.839 | -0.00066 | 0 | 0.00073 | 0.00253 |
| 1014.408 | -0.00073 | -0.00011 | 0.00063 | 0.00242 |
| 1012.977 | -0.00077 | -0.00025 | 0.00045 | 0.00231 |
| 1011.547 | -0.00077 | -0.00029 | 0.0003 | 0.00229 |
| 1010.116 | -0.00074 | -0.00024 | 0.00026 | 0.00233 |
| 1008.685 | -0.00073 | -0.0002 | 0.00033 | 0.00238 |
| 1007.254 | -0.00074 | -0.00026 | 0.00044 | 0.00239 |
| 1005.824 | -0.00077 | -0.00039 | 0.00053 | 0.00239 |
| 1004.393 | -0.00078 | -0.00047 | 0.00057 | 0.00243 |
| 1002.962 | -0.00075 | -0.00044 | 0.00057 | 0.0025 |
| 1001.531 | -0.00069 | -0.00029 | 0.00056 | 0.00256 |
| 1000.101 | -0.00061 | -0.00011 | 0.00056 | 0.0026 |
